# To mulch or not to mulch? Effects of gravel mulch toppings on plant establishment and development in ornamental prairie plantings

**DOI:** 10.1371/journal.pone.0171533

**Published:** 2017-02-06

**Authors:** Anja Schmithals, Norbert Kühn

**Affiliations:** Department of Landscape Architecture and Environmental Planning, Technische Universität Berlin, Berlin, Germany; Shandong University, CHINA

## Abstract

In recent years, North American prairie vegetation has served as a design model for highly attractive, low-cost and low-maintenance plantings in German urban green spaces. Where mixed-planting techniques, gravel mulch toppings and non-selective maintenance techniques such as mowing are used, prairie plantings are considered to be cost-effective alternative design concepts for public green space management. In this study, we investigated the establishment success of different mixtures of prairie species plantings on two sites with different soil conditions: topsoil and topsoil with graywacke gravel topping. We documented significantly higher average mortality rates on gravel mulch sites in the first year after establishment. Further development of mortality was not significantly different between sites. Weed species were always more numerous on topsoil sites and had an obvious effect on the visual impact of the plantings. The mulch created an effective barrier for wind-dispersed germinators. Soil temperatures down to 30 cm were significantly higher on gravel mulch sites throughout the year, stimulating more vital plant growth and a prolonged growing season. Our results emphasize the importance of considering these kinds of practical issues during the planning process as they are critical to the success or failure of the design.

## Introduction

The characteristic and beautiful prairie ecosystem became a source of inspiration for German landscape designers around the early 1990s. Pioneering projects included a prairie planting at the National Garden Show (BuGa) 1991 [1], the prairie lakeside terraces at the International Garden Show (IGA) 1993 [2] and the prairie garden in the Berggarten/Hannover, which was established in 1997 [3]. All of these designs were based on Hansen’s theory of perennials in their garden habitats [4], specifically on vegetation types of the open ground. At about the same time, a number of German botanical gardens established prairie plantings of a more naturalistic style (e.g. Bayreuth 1989, Frankfurt a. M. 1990).

Oehmichen [5], Rothmund [6] and de la Fleur [7] were the first to discuss the suitability of the prairie ecosystem as a model for German horticulture in the context of shrinking maintenance budgets for public green spaces. While acknowledging that climatic differences would have to inform species choices, they argued it should be possible to create easy to care for and highly ornamental plantings [6]. Self-regulating processes within the prairie plant community were expected to make it possible to maintain the plantings permanently by simply mowing once per year. High stability and invasion resistance were predicted for ornamental prairies: they would be “attractive, persistent, low-maintenance” [8]. To achieve a high visual impact, however, it was understood that the typical grass-to-forb ratios of natural prairie ecosystems would have to be adjusted. The inversion of the usual ratio (e.g. [9]) would yield the desired visual effect of plantings that were rich in flowers and low in grasses [7], but did not, however, conform to the original idea of profiting from the regulating processes of naturally balanced ecosystems. This contradiction, rather than being questioned, was widely ignored.

As a consequence of the promotion of prairie vegetation in German horticultural magazines for many years (e.g. [8, 10, 11]), forb-rich ornamental prairie plantings were created in many German municipalities, e.g. Berlin [12], Frankfurt a. M. and Worms [13], most commonly in traffic areas such as roundabouts and road verges, as well as in public parks.

Most of these plantings were established in combination with a gravel mulch top layer, which has recently become a state-of-the-art technique in contemporary German planting design [14–17]. Gravel mulch is regularly used to support design ideas (e.g. by enhancing color schemes or by offering rough or smooth textures through the choice of crushed or pebbled material), to reduce the number of weeds [18], to improve the establishment of target vegetation and to reduce ongoing maintenance [14, 15]. The precise choice of rock material is important for the actual effectiveness and long-term performance of the mulch layer (pH value, erosion and aggradation characteristics) [18]. It is widely assumed that gravel mulch layers result in increased soil temperatures during the summer months owing to intense surface warming and that this has positive effects on plant performance. The actual effect, however, has not been analyzed before; therefore the true impact on plant performance is speculative.

Existing public prairie plantings on gravel mulch sites have been neither scientifically monitored nor critically evaluated. As a consequence, there is no documented information on the establishment success and long-term development of such plantings in Germany, nor are any reliable data available on actual management requirements. It remains unproven whether mulched prairie plantings allow for low-maintenance, and at best self-sustaining, green space management. In addition, it is unknown whether the predicted stabilizing processes actually develop in artificial prairie plant communities when natural species diversity is significantly reduced and natural abundances of species have been strongly manipulated for aesthetic reasons.

The prairie landscapes at the botanical gardens of Bayreuth and Frankfurt, which were planted on in-situ topsoil, proved to be highly dynamic due to a massive invasion of German native weedy species and directional shifts in the dominance among the planted species. The plantings became highly grass-dominated. Species diversity continued to decrease over time and ultimately the plantings lacked any horticultural appeal (deconstruction and modified reestablishment took place in 2006 and 2002, respectively).

With these experiences in mind, and based on the knowledge that sustainable native prairies are dominated by grasses as well (e.g. [9]), it is of great economic interest as to whether gravel mulched, ornamental, forb-dominated prairie plantings might actually persist without significant maintenance, thereby adding to the portfolio of low-budget concepts for German public green spaces.

The aim of this research project was to investigate the performance and potential for optimization of a state-of-the-art prairie planting concept, as it is commonly realized in public German green spaces. We focused on the general effect of gravel mulch on the establishment and development of a community of prairie forbs and grasses in Germany over three years. Specific questions addressed in the study were as follows:

- What effects do gravel mulch toppings have on the establishment success of prairie plantings and individual prairie species?
- What effects do gravel mulch toppings have on soil temperatures?
- What effects do gravel mulch toppings have on the presence and abundance of weed species?
- What effects do gravel mulch toppings have on the vitality of prairie species?

## Materials and methods

### Ethics statement

N/A. No endangered or protected species were involved in this study. Field permission was granted by the Department of Landscape and Urban Planning, Chair of Vegetation Technology and Planting Design, of the Technische Universität Berlin.

### Experimental setup and preparation

**Fig 1:**
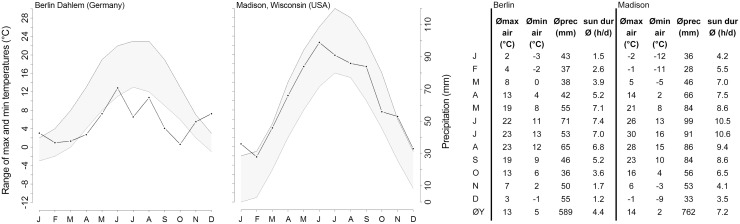
Climate data for Berlin (Germany) compared to Madison (Wisconsin, USA).

The experiment was undertaken in the southwestern part of Berlin (52°27′ 18.77″N, 13°17′54.90″E). The long-term average rainfall per year is 589 mm. Rainfall distributions over the year, temperature patterns and other climatic parameters are shown in Fig 1. While total annual rainfall values and average maximum and minimum temperatures are not substantially different from native tall grass prairie habitats (e.g. Madison, Wisconsin), the rainfall distribution during the year differs greatly, with higher winter and lower summer precipitation in Berlin. Berlin winters tend to alternate between periods of frost and periods of mild wet weather with above-freezing temperatures. The summers tend to be dry and warm with a dip in precipitation in midsummer.

The site was formerly used for experimental vegetable gardening and was abandoned in the summer of 2008. After clearing, the site was rototilled to 40 cm in late 2008. In 2009, *Sinapis alba* was sown as an intermediate crop. The site was cleared again at the end of summer 2009. Five soil samples each of the topsoil site and the mulched site were taken from a depth of 30 cm and were analyzed for pH, P, K, Mg, N min, C/N ratio and classified soil type (see S1 Table for details [19]) to certify the sites’ suitability for the intended use. The soil was classified as a loamy to slightly loamy sand. The pH values at the site averaged 6.8, which allows for good plant nutrient availability [20]. C/N values were above optimum (~10) but still below critical values (>20) [21]. Plant-available nitrogen levels were within the desired range of 25–125 kg/ha for prairie plantings, depending on the individual species and composition [22]. K and Mg supply was sufficient. The very high P values are the result of heavy fertilizing during previous uses of the site. The study design was developed to allow for a number of factors to be considered efficiently:

1. Soil treatment (in-situ topsoil, 7 cm graywacke chippings 5/11 on top of in-situ topsoil)
2. Species diversity (32, 16, 8 species)
3. Grass-to-forb ratio (80:20, 50:50, 20:80)
4. Management type [mowing (m), m + selective weeding (s), m + s + burning in early spring (b)]

The focuses of this article are the effects of soil treatment on the establishment of the overall planting and the individual species, on weed species, on soil temperatures and on the vitality of prairie species. Research into the effects of the other factors is presented in a separate publication [23].

A four factorial split-plot-block design [A/(B+C)/D] was established (overall size 43.20 m × 22.60 m) in September 2009. The non-randomized layout was chosen in order to provide for efficient and controllable management conditions (especially with respect to burning) as well as the reduction of edge effects between treatments.

We developed nine species mixes (three different species diversities combined with three different grass-to-forb ratios). Species were selected on the basis of suitability for dry-mesic to mesic soils, medium size and growth, attractiveness of flowers or seed heads, wild-growing origin and known or assumed horticultural potential. Three C4 grasses and 29 forb species were chosen. Transient forb species were included for the initial effect of the plantings, slow developers for long-term aspects. These mixes were established in a 3 × 3 grid (3.60 m × 4.80 m each). Each of these plots was divided into three subplots (3.60 m × 1.60 m) for the different management types. This basic unit was planted into (1) topsoil and (2) topsoil with a graywacke gravel mulch topping, giving the study design 9 × 6 individual factor combination units. Three replicates were established (see S1 Fig for study design layout).

Plots were marked by wooden pegs at the corners. A 100 cm geomembrane was installed around the site to inhibit clonal immigration of nearby turf grasses and to reduce seed invasion. The same was done between the two soil treatment sites to prevent the gravel mulch from moving into the topsoil plots and to reduce the transfer of prairie plant seeds between soil treatment plots.

The choice of mulch material was based on local availability, soil-chemical characteristics of the parent material, price and visual quality.

The seeds were obtained from different sources in the USA and Germany. Two nurseries carried out the cultivation according to their species expertise and logistical capacities.

The intended species composition and the field experiment establishment agenda had to be adjusted after several species failed to cultivate. Five species had to be excluded completely from the study for lack of sufficient replacement seed material. *Liatris ligulistylis*, *Oenothera macrocarpa*, *Penstemon hirsutus*, *Pycnanthemum pilosum* and *Solidago missouriensis* were cultivated as substitute species between winter 2009 and summer 2010 in university green houses. The original cultivation of *Dalea purpurea*, *Penstemon grandiflorus* and *Verbena stricta* failed to produce the quantities required for the original study layout; those species were supplemented through additional cultivation, and their planting was delayed, as well.

In September 2009, 25 species were planted. We used seedling plants with miniature root balls (approx. ø 4 cm) as an inexpensive alternative to traditional 9-cm containers. In addition to the substantial financial savings, seedlings of native plants have been shown to have better rooting and faster growth and to achieve an early closure of the target vegetation cover in extensive plantings [24].

Plants were introduced in an 8 × 18 grid mixed planting with a planting density of 25 individuals/ m^2^. We chose a mixed-planting technique for the arrangement of the plants, because it is an efficient way of establishing and maintaining extensive herbaceous plantings [25–29], including prairie plantings [30, 31]. Due to the meadow-like and irregular distribution of the plants, small changes in the original design or minor weed growth are not readily perceived as an unwanted development, and therefore the need for immediate grooming action is reduced. This kind of modular distribution of plants facilitates and accelerates the planning process as well as the actual planting process, thereby reducing development and installation costs [32].

Open spaces in the matrix were marked with Tonkin canes. The site was irrigated daily for 10 days after planting by two full-circle sprinklers. After that, no further maintenance was undertaken until late winter. Substitute species were planted in September 2010. For final species choice, ecological and horticultural characteristics and species mixture composition see Tables 1 and 2.

**Table 1:** Ecological and horticultural characteristics of study species.

species	life span	compositional function based on Hansen (1981)	natural habitat	moisture requirements	blooming aspect and flower season in Berlin
*Agastache foeniculum*	short	companion perennial	prairies	dry	violet-blue triangular lance, Jul-Aug
*Amorpha canescens*	long	specimen perennial	prairies, sandy habitats	dry	purple-orange spikes, Jul-Sep
*Asclepias tuberosa*	medium	theme perennial	prairies, sandy habitats	dry	orange in wide clusters, Aug-Sep
*Asclepias verticillata*	long	filling-in perennial	prairies, open habitats	dry-mesic	several white flowers in a cluster, Aug
*Aster oblongifolius*	medium	companion perennial	open habitats	dry	violet-blue daisies, mid Aug-Oct
*Baptisia australis*	long	specimen perennial	prairies, rocky habitats	moist	blue-violet racemes, Jul
*Bouteloua curtipendula*	medium	grass matrix	prairies, sandy habitats	dry	purplish flower, Aug-Sep
*Dalea purpurea*	medium	filling-in perennial	prairies, open habitats	dry	rose-purple cylindrical clusters, Jul-Aug
*Echinacea angustifolia*	medium	theme perennial	prairies	dry	purple daisies, Jul-Aug
*Echinacea pallida*	medium	companion perennial	prairies	dry-mesic	pale pink daisies, Jul-Aug
*Echinacea paradoxa*	medium	companion perennial	prairies	dry	yellow daisies, Jul-Aug
*Eryngium yuccifolium*	medium	specimen perennial	prairies, open habitats	dry-moist	white-green thistlelike globular head, Jul-Aug
*Liatris aspera*	medium	companion perennial	open habitats	dry	purple-pink spikes, Aug-Sep
*Liatris ligulistylis*	medium	theme perennial	open habitats	mesic-moist	purple-pink spikes, Aug-Sep
*Monarda bradburiana*	short	companion perennial	open habitats	mesic	pink-purple cluster, Jul-Aug
*Oenothera macrocarpa*	medium	filling-in perennial	prairies, rocky habitats	dry	yellow rhomboidal flower, Jul-Sep
*Oligoneuron album*	short	theme perennial	prairies	dry	white daisies, Aug-Sep
*Parthenium integrifolium*	medium	companion perennial	prairies, woodlands	dry	white clusters, Jul
*Penstemon grandiflorus*	medium	companion perennial	prairies, open habitats	dry	lilac-blue tubular flowers, Jun-Jul
*Penstemon hirsutus*	medium	companion perennial	open habitats, woodlands	dry	purple-white tubular flowers, Jun-Jul
*Penstemon ovatus*	medium	companion perennial	prairies	dry-mesic	blue tubular flowers, Jun-Jul
*Pycnanthemum pilosum*	medium	companion perennial	woodlands	dry	white clusters, Jul
*Pycnanthemum tenuifolium*	medium	companion perennial	prairies, woodlands	dry	white clusters, Jul
*Ratibida columnifera* var. *pulcherrima*	short	filling-in perennial	prairies, open habitats	dry	red flowers, Jul-Sep
*Rudbeckia hirta*	short	filling-in perennial	prairies	dry-mesic	orange daisies, Jul-Aug
*Rudbeckia missouriensis*	medium	companion perennial	open habitats	dry	yellow daisies, Aug-Sep
*Rudbeckia triloba*	short	theme perennial	open habitats, woodlands	moist	yellow daisies, Aug-Sep
*Schizachyrium scoparium*	medium	grass matrix	prairies, open habitats	dry	grayish spikes, Sep
*Solidago missouriensis*	medium	theme perennial	prairies, open habitats	dry	small yellow flowers in plume-shaped head, Aug-Sep
*Sporobolus heterolepis*	medium	grass matrix	prairies, open habitats	dry	pink-bronze in airy panicles, Aug-Sep
*Verbena hastata*	short	companion perennial	prairies, open habitats	moist	blue-violet pencillike spikes, Jul-Aug
*Verbena stricta*	short	companion perennial	prairies, open habitats	dry	purple-white pencillike spikes, Jul-Aug

Information on natural habitats and moisture requirements from [33].

**Table 2:** Species mixture composition.

mixture	A	D	G	B	E	H	C	F	I
number of species in the mixture	32			16			8		
grass-to-forb ratio	80:20	50:50	20:80	80:20	50:50	20:80	80:20	50:50	20:80
	quantity of individuals per species in each mix								
*Agastache foeniculum*	1	2	3						
*Amorpha canescens*	1	2	2	1	1	2			
*Asclepias tuberosa*	1	2	6	2	6	10	7	15	29
*Asclepias verticillata*	1	3	4						
*Aster oblongifolius*	1	3	5	2	6	9			
*Baptisia australis*	1	1	2	1	1	2	1	2	3
*Bouteloua curtipendula*	38	24	9	38	24	9	38	24	9
*Dalea purpurea*	1	2	1						
*Echinacea angustifolia*	1	4	7	4	9	14	9	24	36
*Echinacea pallida*	1	3	4						
*Echinacea paradoxa*	1	2	4						
*Eryngium yuccifolium*	1	3	5						
*Liatris aspera*	1	3	4						
*Liatris ligulistylis*	1	4	6	4	8	12			
*Monarda bradburiana*	1	2	3						
*Oenothera macrocarpa*	1	2	4	3	7	11			
*Oligoneuron album*	1	2	3	2	6	9	7	18	30
*Parthenium integrifolium*	1	2	4						
*Penstemon grandiflorus*	1	2	3						
*Penstemon hirsutus*	1	3	4	2	8	12			
*Penstemon ovatus*	1	3	4						
*Pycnanthemum pilosum*	1	3	4						
*Pycnanthemum tenuifolium*	1	3	5						
*Ratibida columnifera* var. *pulcherrima*	1	2	4						
*Rudbeckia hirta*	1	2	4						
*Rudbeckia missouriensis*	1	2	5	2	7	11			
*Rudbeckia triloba*	1	3	4	2	4	6	5	13	17
*Schizachyrium scoparium*	38	24	10	38	24	10	38	24	10
*Solidago missouriensis*	1	2	3	2	4	7			
*Sporobolus heterolepis*	39	24	10	39	24	10	39	24	10
*Verbena hastata*	1	2	4	2	5	10			
*Verbena stricta*	1	3	4						

### Data collection and study site maintenance

To address questions of initial establishment success of the mixtures and of individual species, survival of planted individuals was recorded in early June (to assess winter mortality) and September (to assess summer mortality) as were cover values of grasses, forbs and bare ground. To study the effect of the gravel mulch on soil temperature, data assessment was undertaken monthly on both soil treatment sites, generally on high-radiation days around midday, at depths of 10, 25, 50, 100, 150, 200 and 300 mm with a high precision digital thermometer (model GMH 3710, Greisinger electronic, Germany) and customized test probe starting in January 2011. Identity and cover of weedy species were determined for all plots in June and September prior to selective weeding measures to address the issue of actual weed suppression by gravel mulch top layers.

Vitality criteria (e.g. number of florescences, number of vegetative shoots) were assessed between 2011 and 2013 for a representative selection of eight species either in July or September, depending on the phenological development of the traits to be recorded. Two characteristic traits per species were chosen. Assessment of vitality aspects was only undertaken within the 32-species plots, where all of the species to be assessed were present, in order to create equally large sampling units. Sample size per species was capped at five individuals per study plot. For all of the grass species this required a permanent marking of the selected individuals from a generally larger amount of stock. For the remaining species, the vitality assessment included all planted individuals.

The site was mowed yearly in late winter to a height of 10 cm. The clippings were raked from the site. Burning was undertaken in mid-March, using a three-wheeled mobile weed burner. The plots were burned until standing vegetation and surface litter were carbonized.

Selective weeding was undertaken monthly between April and September. Weeds growing above the general plant canopy were generally pulled by hand. All woody vegetation (mainly *Betula pendula*, *Acer platanoides*, *Salix* spp.) and the forbs *Cirsium arvense* and *Humulus lupulus* were consistently removed regardless of size using tools if necessary.

### Limitations of the study

Short term changes of the originally intended species composition became necessary due to the complete failure of cultivation of some species or incomplete delivery of other species. A large percentage of delivered *Echinacea angustifolia*, *Oligoneuron album* and *D*. *purpurea* were of insufficient quality, which may explain the high mortality rates during the initial establishment phase.

Because study site establishment occurred over two years, it is difficult to compare first year mortality rates, since establishment conditions differed greatly between years (two weeks of unfavorable weather after planting in 2009; safe-site effects for seedling establishment due to standing vegetation in 2010 accompanied by moderate temperatures); for climate details at time of planting see S1 File [50]. From 2012 on, mice herbivory heavily affected both *Liatris* populations and *E*. *angustifolia* (no further investigation).

### Statistical analysis

Where data on plot-based average mortality rates were not sufficiently normal for parametric analysis, they were either improved by logit transformation or the Kruskal-Wallis test was used. Where nonnormal data could not be adequately improved (individual species mortality rates, vitality data and soil temperature), we used the Wilcoxon-Mann-Whitney test. Data on weed species numbers were analyzed via generalized linear modeling (Poisson or negative binomial distribution depending on the dataset variance/mean proportions). Data on weed species cover values were analyzed using generalized linear modeling with a Tweedie distribution. Potential effects of treatments B, C and D were incorporated in the models as error terms where necessary. Statistical tests were undertaken using R 3.0.2 [34].

## Results

### General establishment of the plantings and individual species

Regardless of soil type, for the 25 species planted in autumn 2009, an average mortality rate of 13.6% (grasses 7.1%, forbs 21.7%) was found in June 2010. For the seven species planted in autumn 2010, the first data assessment took place in June 2011 and an average mortality rate of 10.1% was recorded (forbs only). The species with the highest average mortality rates in the first summer after planting were *Rudbeckia hirta* (74.5%) and *Verbena hastata* (71.5%). Species with higher average mortality rates were *Ratibida columnifera* var. *pulcherrima* (44.9%), *Baptisia australis* (36.3%) and *E*. *angustifolia* (35.0%). The species which established themselves most successfully were *O*. *album* (average mortality rate 0.9%), *P*. *pilosum* (1.1%), *Rudbeckia triloba* (1.1%), *L*. *ligulistylis* (1.7%) and *Eryngium yuccifolium* (2.0%).

By September 2012, nearly half of the 32 species had average mortality rates of close to or more than 50%. Five species had either completely or nearly died out: *R*. *hirta* (100%), *R*. *columnifera* var. *pulcherrima* (98.6%), *D*. *purpurea* (98.1%), *B*. *australis* (96.9%) and *V*. *hastata* (92.6%). The five long-term top performers were *Schizachyrium scoparium* (mortality rate 4.9%), *O*. *album* (5.2%), *Pycnanthemum tenuifolium* (6.0%), *Sporobolus heterolepis* (6.1%) and *E*. *yuccifolium* (6.8%).

### Effect of gravel mulch topping on plot-based average mortality rates and on individual species mortality rates

Because initial plantings took place in two different years, results for the first two growing seasons are presented separately for the groups of species (25 species planted in 2009 vs.7 species planted in 2010), as well as for the overall planting of 32 species, which results in a combination of data from different years of cultivation. Gravel mulch plots had significantly higher average mortality rates in the first year after the 2009 planting (topsoil 10.7%, gravel mulch 16.6% for 25 species in June 2010, P<0.001***), while differences for the seven species planted in 2010 were not significant.

**Fig 2:**
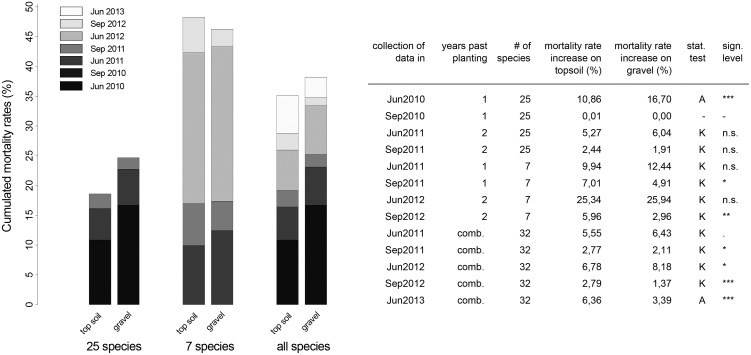
Comparison of average mortality rates of topsoil and gravel mulch plots between Jun2010 and Jun2013. Significant differences between soil types are indicated by asterisks. P<0.1 * P<0.05 ** P<0.01 *** P<0.001 A = ANOVA K = Kruskal-Wallis-Test.

Subsequent mortality results were inconsistent between soil treatments (Fig 2): summer mortality was always higher on topsoil sites and winter mortality was generally higher on gravel mulch sites for all species groupings for the duration of the study. After the last assessment in Jun 2013, the total average mortality rate was 35.12% on topsoil plots and 38.18% on gravel mulch plots.

At the species level, average mortality rates varied considerably between soil treatments. Significant differences were verified for 12 of the 32 species (significant results at P<0.05* in at least two consecutive assessments, or at P<0.1. for the majority of the study). Of these 12 species, only two produced lower mortality rates on the mulched site (*Amorpha canescens*, *P*. *grandiflorus*); all others survived more frequently on topsoil.

While for some species significant differences occurred primarily in the initial phase of the study (i.e. *Asclepias tuberosa* (P<0.001), *R*. *hirta* (P<0.001), *B*. *australis* (P<0.01), *O*. *macrocarpa* (P<0.1)and *P*. *grandiflorus* (P<0.01)), others showed ongoing and consistent differences in mortality behavior for most of the data assessments (*A*. *canescens* (P<0.1), *Aster oblongifolius* (P<0.001), *Bouteloua curtipendula* (P<0.001), *R*. *columnifera* var. *pulcherrima* (P<0.001)), while still others were significantly different only in later stages of the study (*Rudbeckia missouriensis* (P<0.001), *R*. *triloba* (P<0.001), *V*. *stricta* (P<0.01)) (Table 3).

**Table 3:** Significant differences in average mortality rates of 12 species on topsoil vs. gravel mulch plots between June 2010 and September 2012.

Species\ ø mortality rate (%)	ts 06 2010	gm 06 2010	P-level	ts 09 2010	gm 09 2010	P-level	ts 06 2011	gm 06 2011	P-level	ts 09 2011	gm 09 2011	P-level	ts 06 2012	gm 06 2012	P-level	ts 09 2012	gm 09 2012	P-level
significant differences during the first growing season																		
*Asclepias tuberosa*	17,4	31,3	***	17,4	31,3	***	36,4	43,1	n.s.	40,4	45,9	n.s.	55,4	58,3	n.s.	64,0	59,5	n.s.
*Rudbeckia hirta*	53,7	97,0	***	54,6	97,0	***	96,3	100,0	n.s.	100,0	100,0	n.s.						
*Baptisia australis*	26,1	46,6	**	26,1	46,6	**	73,3	89,0	*	90,5	93,2	n.s.	95,1	96,8	n.s.	96,3	97,5	n.s.
*Oenothera macrocarpa*							14,4	29,2	*	20,3	33,9	*	34,5	42,6	n.s.	43,0	48,9	n.s.
*Penstemon grandiflorus*							42,0	13,6	**	48,8	21,6	**	53,7	40,1	n.s.	57,4	43,2	n.s.
significant differences during the majority of the assessments																		
*Amorpha canescens*	16,7	3,7	*	16,7	3,7	*	20,4	6,5	*	20,4	6,5	*	23,1	15,7	n.s.	23,1	16,7	n.s.
*Aster oblongifolius*	8,5	20,8	**	8,5	20,8	**	9,3	27,3	***	9,5	29,6	***	10,0	34,2	***	10,5	36,3	***
*Bouteloua curtipendula*	5,9	23,0	***	5,9	23,0	***	9,3	31,2	***	10,2	32,5	***	13,4	36,2	***	14,7	37,5	***
*Ratibida columnifera var*. *pulcherrima*	25,0	64,8	***	25,0	64,8	***	53,7	88,0	***	56,5	88,0	**	88,0	100,0	*	97,2	100,0	n.s.
significant differences after the first growing season																		
*Rudbeckia missouriensis*	5,3	5,3	n.s.	5,3	5,3	n.s.	5,6	11,4	n.s.	5,6	11,6	n.s.	7,9	29,8	***	8,3	30,5	***
*Rudbeckia triloba*	0,9	1,4	n.s.	0,9	1,4	n.s.	17,5	38,2	***	21,7	41,6	***	56,8	86,9	***	61,3	89,7	***
*Verbena stricta*							11,5	23,8	n.s.	12,5	28,7	n.s.	21,8	51,9	**	25,0	56,5	**

ts = topsoil gm = gravel mulch

Significant differences between soil types are indicated by asterisks (Wilcoxon-Mann-Whitney-Test).

* *P*<0.05

** *P*<0.01

*** *P*<0.001

n.s. = not significant

Note that data for 2010 is missing for species introduced in the second year planting process.

### Effect of gravel mulch topping on soil temperature

**Fig 3:**
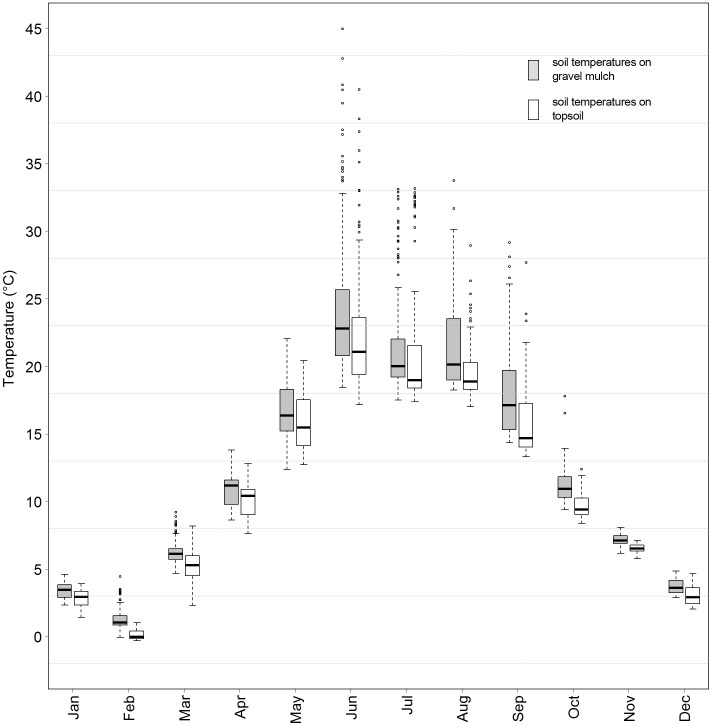
Differences in soil temperature on topsoil vs. gravel mulch plots.

Significant differences existed between temperature gradients in soil with gravel mulch topping vs. in-situ topsoil. Measurements at the 10, 25 and 50 mm level occurred either within the gravel mulch top layer or the in-situ topsoil. All deeper measurements were located within the in-situ soil horizon. During the course of a complete measurement cycle (duration of approx. 2 h), continuous temperature increases were found at all tested depths, especially during the summer months. We compensated for this in the data analysis by using the average value of all ten measurement locations for each soil variable. Fig 3 illustrates average differences in soil temperature between topsoil and gravel mulched sites (data combination of 2011 and 2012 at all depth levels). S2 File gives monthly climate data for both years of measurement [35].

Average gravel mulch soil temperatures were always significantly higher than the respective topsoil values (*P*<0.001). Depth related data analysis revealed consistently warmer temperatures below gravel mulch topping for all depth levels.

**Fig 4:**
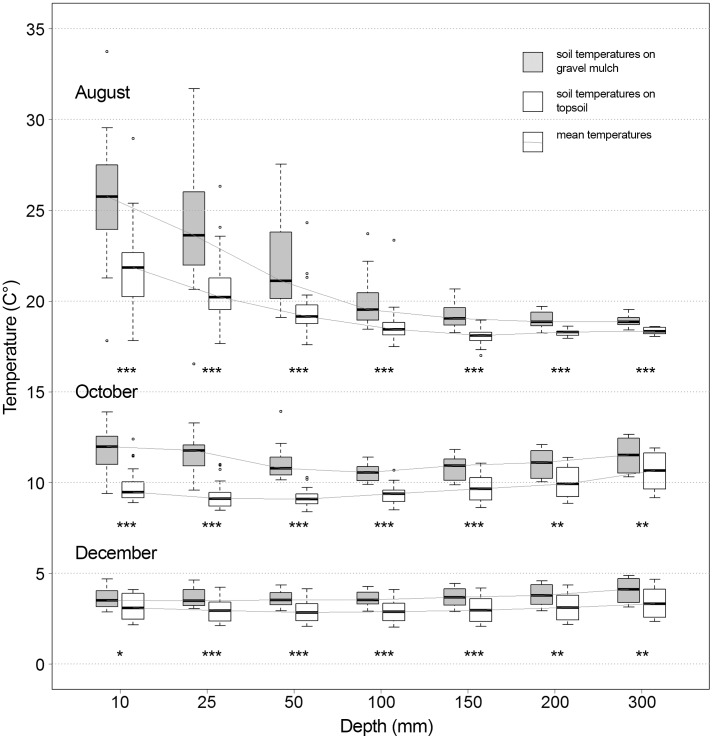
Comparison of average topsoil and gravel mulch soil temperatures along a depth gradient. Significant differences between soil treatments are indicated by asterisks (Wilcoxon-Mann-Whitney-Test). P<0.1 * P<0.05 ** P<0.01 *** P<0.001 n.s. = not significant.

Fig 4 illustrates the change in average soil temperature with increasing soil depth for a representative selection of months (August, October and December). There is a clear overall tendency for the temperature curves of the two soil treatments to change in parallel.

### Effect of gravel mulch topping on weed species

A total of 121 different weed species were identified on the study site during five assessments in June and September of 2011 and 2012 and June 2013 (for a complete list see S2 Table). While 61 species occurred on both soil types, 37 species were restricted to topsoil plots and 23 species to mulched sites. Topsoil-only species were mostly of low growth and low root mass and included moisture-, compaction- and siltation-indicator species [36] such as *Sagina procumbens*, *Hernaria glabra*, *Matricaria discoidea* and *Rorippa palustris* as well as many species with rosette-forming ground leaves such as *Arenaria serpyllifolia*, *Campanula rapunculus*, *Crepis capillaris* and *Myosotis arvensis*. The spectrum of species exclusive to gravel mulched sites was very unspecific. It included low frequencies of species with a variety of growth characteristics.

**Fig 5:**
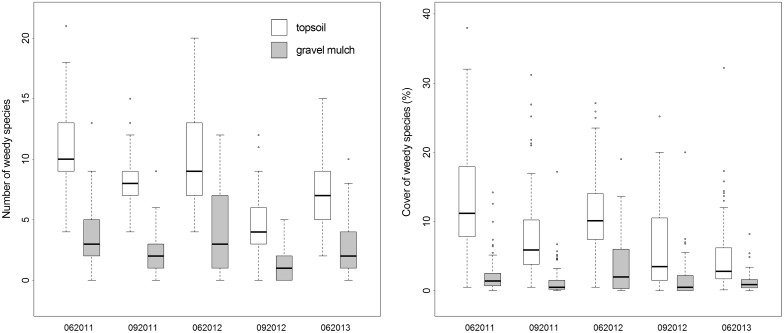
Number and cover of weedy species on topsoil vs. gravel mulch plots between June 2011 and June 2013.

Quantity and area cover values of weedy species greatly differed between soil types without exception for all five assessments. Topsoil sites always had higher average weed species numbers and weed species cover values than gravel mulch sites (Fig 5).

### Effect of gravel mulch topping on individual species vitality

Vitality criteria of all evaluated species showed significant effects of soil treatment (Table 4).

**Table 4:** Assessment criteria and significance of soil treatment on vitality of eight prairie species.

	data coll. in June	data coll. in September	basal Ø (cm)	clump Ø (cm)	# of gen. shoots	# of veg. shoots	height of longest shoot (cm)	original sample size	av. survival rate (%)	sig. influence of substrate on 1st vitality trait	on 2nd vitality trait	av. survival rate (%)	sig. influence of substrate on 1st vitality trait	on 2nd vitality trait	av. survival rate (%)	sig. influence of substrate on 1st vitality trait	on 2nd vitality trait
Species			choice of two traits						2011			2012			2013		
Amorpha canescens		x			x	x		90	93,3	ns	ns	80,0	ns	ns	73,3	**	ns
Bouteloua curtipendula		x	x		x			270	98,9	***	ns	90,0	***	*	85,5	ns	.
Eryngium yuccifolium	x				x		x	164	-	-	-	85,9	***	ns	80,5	***	*
Monarda bradburiana	x			x	x			108	77,7	***	*	76,8	*	ns	71,3	ns	.
Parthenium integrifolium	x		x		x			126	92,1	ns	**	91,3	***	**	75,4	**	**
Schizachyrium scoparium		x	x		x			270	100,0	***	***	99,6	*	***	97,8	***	***
Solidago missouriensis		x	x		x			108	98,1	*	***	90,7	**	***	-	-	-
Sporobolus heterolepis		x	x		x			270	99,3	***	ns	99,3	***	ns	98,5	***	***

Significant differences between soil types are indicated by asterisks (Wilcoxon-Mann-Whitney-Test). P<0.1

* P<0.05

** P<0.01

*** P<0.001

ns = not significant

Only two species preferred topsoil growing conditions (*A*. *canescens* and *B*. *curtipendula*), whereas the majority thrived on gravel mulch sites: *S*. *scoparium*, *E*. *yuccifolium*, *Parthenium integrifolium* and *S*. *missouriensis* grew significantly larger diameters on gravel mulch sites. Numbers of flowering shoots were higher on mulched sites for *Monarda bradburiana*, *P*. *integrifolium* and *S*. *missouriensis*.

*S*. *heterolepis* showed ambivalent development and produced smaller basal diameters on gravel mulch sites in 2011 and 2012; the trend was reversed in 2013, accompanied by significantly higher numbers of flowering shoots.

**Fig 6:**
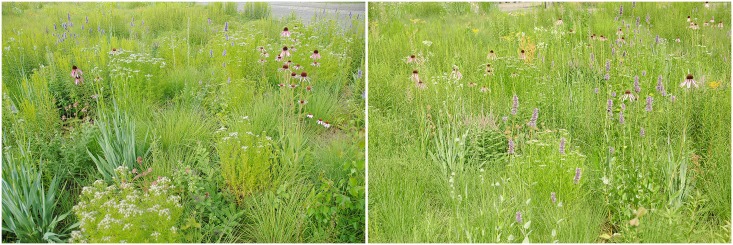
Comparison of gravel mulch vs. topsoil planting appearance in July 2012.

At the end of the study, the following species had significantly higher vitality values on gravel mulch: *E*. *yuccifolium*, *M*. *bradburiana*, *P*. *integrifolium*, *S*. *scoparium* and *S*. *heterolepis*. This contributed to a general visual impression of enhanced vigor on the gravel mulch site (no further assessment). The overall appearance of the plantings greatly differed: topsoil plantings developed a meadow-like and rather homogeneous structure; mulched plantings maintained the visual impression of a grid planting of vital individual plants for a long time (Fig 6).

## Discussion

### General establishment of the plantings and individual species

September 2009 provided extreme weather conditions: above average numbers of warm and hot days, 139% of average sunshine duration and 67% of average rainfall (S3 File). This may have contributed substantially to the establishment success and further development of the initial planting (recorded average mortality rate of 13.6% during the first assessment in June 2010). Many undersize seedlings may have withered when their roots failed to penetrate into the surrounding soil during the first part of the grow-in phase despite regular watering during the first two weeks after planting. Additionally, plants may have died from desiccation during dry winter periods owing to insufficient deep-rooting or from frost heave (observed sporadically).

First-year average mortality rates for the seven species planted in 2010 were slightly lower (10.1%). Weather conditions after the second year planting were not as severe as during the first planting (above average rainfall, below average temperatures and sunshine duration, see S3 File), and the existing vegetation may have provided ‘safe sites’ against heat and wind for the juvenile seedlings (as described in [37]).

During the third year of the study, competitive effects among well-developed individuals of the target species may have accounted for some of the losses as well. By 2012, many of the original seedlings had grown into successful specimens, generally limiting local light availability and increasing below-ground competition. *S*. *missouriensis* showed a high rate of tillering, which led to locally dominant populations covering up to 75% of some plots towards the end of the study. Similar effects of competitive exclusion may have been caused by the weed *Calamagrostis epigeios*, which established dominant and uncontrollable populations on many of the topsoil study sites.

Regardless of soil treatment, here we address the species which generally failed to establish successfully (total average mortality rates ≥ 50% at the end of the study, values in parentheses). Of these 12 species, 3 species are naturally short-lived annuals or biennials: *R*. *columnifera* var. *pulcherrima* (98.6%), *R*. *hirta* (100%) and *R*. *triloba* (75.5%). They were included for initial effect in the plantings and were expected to be replaced by their offspring. Planting material of *D*. *purpurea* (98.1%) was of generally poor quality (primary root only), which was very likely a major factor in its establishment failure. The same was true for *E*. *angustifolia* (77.8%). Additionally, its population was heavily affected by mice herbivory from 2012 on (digging and feeding on the roots and rhizomes, no further investigation). Herbivore preferences for *Echinacea* and *Liatris* species are well documented for leaves and seeds [38, 39], as well as for the tuberous roots [40, 41]. *L*. *ligustylis* (75.0%) and *Liatris aspera* (57.1%) were both equally affected by mice herbivory. Most individuals of *Penstemon ovatus* (73.0%) died shortly after blooming, which was interpreted as a tendency to ephemerality under the present growing conditions. The *A*. *tuberosa* population (61.7%) suffered from consistently high winter mortality. The species is drought tolerant, preferring dry to moist, well-drained soils [42, 43] and might therefore have suffered from inadequate drainage and temporary water logging during winter months. The surviving individuals thrived and reproduced effectively, and seedling plants have been observed. Of the surviving *Asclepias verticillata* (55.2%) individuals, the ones growing close to the edges of the study site were observed to thrive and spread more than the others, which may be an effect of the species’ limited shade tolerance in the face of densely growing, increasingly tall competitors [44]. Only two individuals of *B*. *australis* (96.9%) survived. Being a slow developer, establishment from delicate miniature plugs may have been the key reason for failure.

### Effect of gravel mulch topping on plot-based average mortality rates and on individual species mortality rates

Mortality on gravel mulch plots was generally a lot higher than on topsoil plots during the first year after planting (Fig 2). The growing-in process on the gravel mulch site took longer (as the roots needed to overcome the distance to the in-situ soil) and was more stressful (higher soil temperatures) than on topsoil, especially under September 2009 climate conditions (S3 File). Since miniature plug plants bring with them only a little material capable of storing nutrients and water, those planted in gravel mulch most likely suffered more from nutrient and water depletion than individuals planted into loamy in-situ soil. More similar average mortality rates for the two soil types in the second and third year after planting support this theory. Consequently, initial care on gravel mulch sites needs to be increased to compensate for the harsher establishment conditions.

Of the 12 species that showed significant differences in mortality between soil variables (Table 3), only two had higher mortality rates on topsoil after the first winter (*P*. *grandiflorus* and *A*. *canescens*).

Five species showed significant differences in mortality rates for the two soil treatments during the initial phase of the study, most likely due to unfavorable establishment conditions associated with the particular soil treatment. Four out of those five species had significantly higher death rates on gravel mulch sites, which contradict the species known preferences for well-drained, dry to mesic soil-conditions, which were more likely met by the mulched sites. This reasoning particularly applies to *A*. *tuberosa* [42]. If drowning during the winter was thus not the most probable cause of death, the plants most likely withered during the growing-in phase.

For species with significant differences in mortality rates during the majority of the assessments, in the soil treatment with the higher mortality, growing conditions were acceptable but not optimal. Except for *R*. *columnifera* var. *pulcherrima*, all species of this group had low to moderate mortality rates. Especially for *B*. *curtipendula*, the higher mortality rates on gravel mulch were unexpected considering its wide range of natural habitats from well-drained sandy soils to heavy clays [45] and the personal experience of the authors (observed sensitivity to wet winter soil conditions). The overall establishment rate after three years was good (73.9%), although it was remarkably lower than that of the other two prairie grasses used in the study (*S*. *heterolepis* 93.9% and *S*. *scoparium* 95.0%).

Three species showed significant differences in mortality behavior specifically at the end of the study, and in all cases, the higher mortality occurred on gravel mulch. While the *R*. *triloba* population most likely decreased due to the natural life span of the individuals, this was not the case for *R*. *missouriensis* and *V*. *stricta*. There was no obvious explanation for this as the habitat preferences of these species should have favored the gravel mulch.

### Effect of gravel mulch topping on soil temperatures

The chosen graywacke material is of medium grayish color and becomes much darker when wet. Intensive surface warming by solar radiation was expected. We were interested in the warming effect on the subjacent soil horizon, assuming that it would penetrate down deep and might therefore have a boosting influence on biological activity and corresponding plant performance [18]. The warming effect continued with increasing depth (Fig 4). We expected the mulch layer mostly to increase the continentality of the soil conditions via more intense soil warming during summer months, but the overall effect of the gravel mulch layer proved to be a general increase in average temperatures throughout the year (earlier warming of lower soil layers in the spring, warmer soil surface temperatures during autumn). For the mulched site, this basically results in site conditions mimicking those several degrees of latitude south of the current study location. The resulting prolongation of the growing period and the buffered temperatures during winter months provide an interesting leeway for the planting design profession. Less-hardy species may be added to the designers’ species portfolio and may be used to create innovative landscapes. Consequently, the effects of milder winter soil temperatures on soil moisture conditions should be taken into account when choosing the species portfolio.

### Effect of gravel mulch topping on weed species

Growth of light-dependent weedy germinators from the in-situ seed bank was efficiently reduced by the mulch cover (Fig 5). Seed-propagated weeds did not substantially disturb the appearance of the gravel mulch prairie plantings due to their lack of cover and short stature. The topsoil sites presented a generally more weedy appearance. For the most part, this was not due to higher numbers and greater cover of weedy species, though. Many of the weedy species which thrived on the topsoil sites were of short stature and had little potential for long-term competitive exclusion of target species (e.g. *Sagina procumbens*, *Trifolium repens*, *Plantago major*, *Veronica arvensis*) [36]. It was instead key weedy species with high numbers of individuals and the generally lower and less vigorous growth of target prairie species on topsoil which contributed to the more meadow-like, intermixed-vegetation appearance (Fig 6). *Taraxacum officinale* and *Conyza canadensis* were the two most numerous weedy species with an easily recognizable general appearance and inflorescence.

The mulch layer did not have any effect on the proliferation of weeds that spread by roots (e.g. *Cirsium arvense*, *Calamagrostis epigeios*) that were present in the vicinity of the study site before the experiment. Seedlings of woody weed species were regularly found on both soil types. *Betula pendula* germinated more effectively on gravel mulch plots, although the nearest trees grew closer to the topsoil site. It is to be expected that *B*. *pendula*, as a pioneer tree species, would successfully colonize the gravel mulch “wasteland”. *Salix capraea*, *Acer platanoides* and *Carpinus betulus* grew more commonly on topsoil, which confirmed the species’ preferences for mesic to moist, rich and rather loamy soils.

Although the gravel mulch layer proved to be most beneficial in terms of weed reduction or suppression, resulting in reduced care requirements, regular maintenance and specifically the control of woody and root-propagated weed species remained necessary [23]. An overall tendency to decreasing numbers and cover values of weeds over time was attributed to the development of an increasingly dense target plant canopy and resulting competitive exclusion of light-dependent germinators.

### Effect of gravel mulch topping on individual species vitality

Soil treatment and the according temperature level had highly significant effects on individual species vitality (Table 4). The permanently higher soil temperatures throughout the root horizon caused a general increase in biological activity and accordingly in nutrient availability [46], leading to various signs of enhanced vigor and generative reproduction. Photosynthetic activity was enhanced early in the growing season and resulted in faster and more prolific plant development [47, 48]. During summer months, mulched site soil temperatures more likely resembled the soil conditions found in natural, typical continental prairie habitats of the Midwest. In spring and autumn months, the higher temperatures caused a prolongation of the growing season [49]. Therefore, target species might have been stimulated to higher productivity by more optimal growth temperatures [50]. Additionally, plant growth on mulched ground was generally less constrained by weedy competitors.

## Application to practice: The pros and cons of gravel mulch toppings on herbaceous plantings

Despite higher mortality rates during the establishment stage, the advantages of gravel mulch top layers are obvious: the established plantings grow more vigorously and produce taller specimens with more flowers than topsoil plantings due to increased soil temperatures and therefore enhanced biological activity. Weeding maintenance is considerably reduced. The lack of organic surface material helps suppress weeds as it is only in the very small areas around the root balls that there is sufficient humus for anemochorous weed species to germinate. The choice of material and the according visual impact of the mulch layer allow for an atmospheric support of planting design ideas.

On the other hand, during the growing-in phase of mulched plantings, increased care, especially watering, is essential to compensate for the rather harsh establishment conditions. Based on our experience in this study, we recommend cool weather autumn planting and four weeks of regular watering depending on soil moisture conditions. Material and installation costs for the mulch layer amounted to 7.17€/m^2^ in this study. This initial investment needs to be considered, but is compensated for by long term savings in maintenance costs.

We recommend the use of species of low to moderate tillering activity. Species known to regularly fall prey to herbivores should be added in disproportionately high numbers to compensate for potential losses. Short-lived species for initial effect should only be included into mixtures of high species diversity, where the potential failure of reestablishment from seed will not be as obvious. Slowly growing species should not be added as seedling plants with miniature plugs but as container grown plants.

From the experience in this study, the following species can be recommended for urban green projects: *A*. *canescens*, *A*. *tuberosa*, *E*. *yuccifolium*, *M*. *bradburiana*, *O*. *album*, *P*. *integrifolium*, *P*. *tenuifolium*, *R*. *triloba*, *S*. *scoparium* and *S*. *heterolepis*.

## Conclusions

This study focused on the effect of different soil treatments on the establishment success of prairie mixed plantings in a Berlin urban context and produced valuable insight on the suitability of the employed species and on establishment techniques. The establishment process itself proved to be less successful on gravel mulch plots than on topsoil. The lack of material capable of storing nutrients and water caused significantly higher mortality rates in gravel mulch sites during the growing-in phase. A gravel mulch layer on top of in-situ soil caused highly elevated surface temperatures in high radiation summer weather and higher soil temperatures across all depth layers throughout the year. This warming process resulted in significantly increased biological activity and enhanced individual development of target plants during summer months, once they were successfully established. Winter site conditions were shifted towards significantly warmer soil temperatures above the frost level on mulched sites, which caused species with a preference for well-drained soils to suffer regularly from temporary water-logging (e.g. *B*. *curtipendula*, *A*. *tuberosa*). The presence of seed-propagated weedy species was significantly reduced on mulched plots, while no effect was found for rhizomatous weeds.

In the mulched plantings, a generally enhanced visual impact, the success of individual target plants and the obvious reduction in weeds need to be weighed against the time and cost of installing the mulch layer and the need for more intense and longer lasting establishment care to compensate for the harsher site conditions.

Our findings will hopefully support the planning process of future projects. Further analysis of how management and species mixture affect the development of the experiment site will be of great interest. Ongoing investigation into the medium- and long-term development of the plantings will be necessary to answer questions on community processes. Successful establishment of sustainable planting designs depends on long-term rejuvenating processes within the plant community, and thus the possibility that the mulch layer will inhibit seed propagation needs to be carefully considered.

## Supporting information

S1 Table Results of soil sample analysis [19]. lS = heavily loamy sand, l'S = slightly loamy sand, gm = gravel mulch, ts = topsoil.
(XLS)

S2 Table Complete list of documented weed species on both soil treatments sorted by species name. (XLSX)

S1 Fig Study design layout. (TIF)

S1 File Climate data at time of planting (Sep. 2009 and Sep. 2010) [35]. (PDF)

S2 File Monthly climate data of 2011 and 2012 [35]. (PDF)

S3 File Original datasets. (ZIP)
