# Supplementary figures and images for: To mulch or not to mulch? Effects of gravel mulch toppings on plant establishment and development in ornamental prairie plantings
Source: PLoS One. 2017 Feb 6;12(2):e0171533. doi: 10.1371/journal.pone.0171533 (PMC5293235; doi:10.1371/journal.pone.0171533)

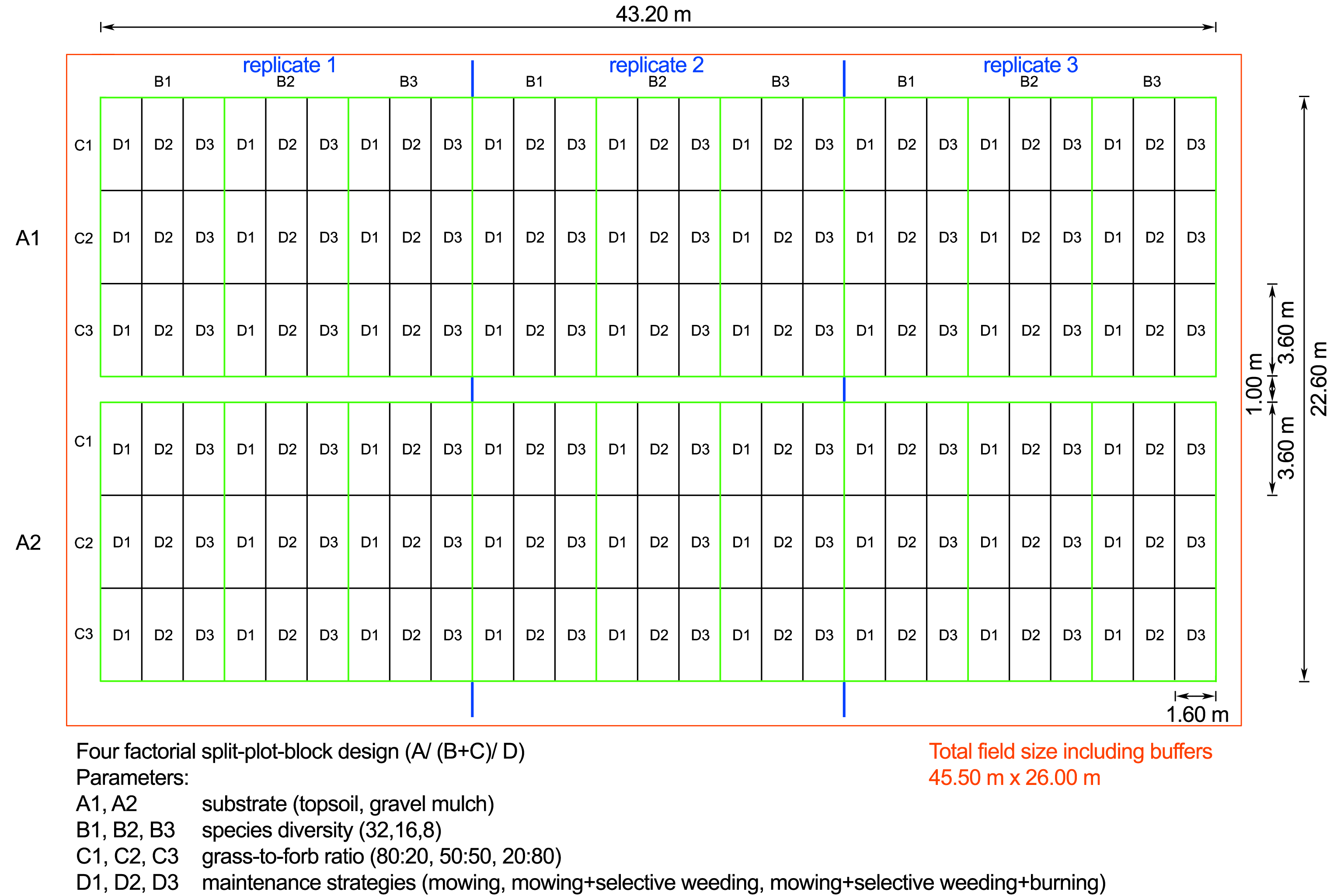

Supplement: S1 Fig — (TIF) [file pone.0171533.s003.tif]
