# Supplementary material for: To mulch or not to mulch? Effects of gravel mulch toppings on plant establishment and development in ornamental prairie plantings
Source: PLoS One. 2017 Feb 6;12(2):e0171533. doi: 10.1371/journal.pone.0171533 (PMC5293235; doi:10.1371/journal.pone.0171533)
Supplement: S1 File — (PDF) [file pone.0171533.s004.pdf]

Beiträge zur Berliner Wetterkarte

Klimatologische Mittelwerte von Berlin-Dahlem

66/09
KBD IX/09

ISSN 0177-3984
01.10.2009

Element	Maßeinheit	Beobachtungsperiode	Vieljähr. Durchschnittswert	Durchschnittswert 1961-90	**September 2009**	Datum	Abweichung vom Durchschnitt 1961-90	in %	Bisherige Extreme 1909-2008	Datum
Luftdruck auf NN reduziert	hPa	1881-1970	1016.5	1016.7	1019.5		+2.8			
Höchster Luftdruck	hPa	1951-1980	1030.1	1030.0	1029.9	11.	-0.1		1038.5+	07.09.1953
Tiefster Luftdruck	hPa	1951-1980	1000.8	1000.4	999.1	03.	-1.3		987.2+	21.09.1990
Temperatur (stündl. Ablesung 01-24 MEZ)	°C	1955-1984	13.7	13.6	15.2		+1.6			
Temperatur (Klimamittel)	°C	1909-1969	13.9	13.5	15.2		+1.7			
Höchste Temperatur	°C	1909-1969	27.0	26.1	32.0	01.	+5.9		34.2	03.09.1911 12.9.1919
Mittlere Maximum-Temperatur	°C	1909-1969	19.3	18.7	20.9		+2.2			
Tiefste Temperatur	°C	1909-1969	3.4	4.0	4.5	30.	+0.5		-0.5	21.09.1915
Mittlere Minimum-Temperatur	°C	1909-1969	9.4	9.4	9.9		+0.5			
Tiefste Temperatur am Erdboden	°C	1951-1980	1.8	2.6	0.3	30.	-2.3		-1.9*	21.09.1997
Mittlere Min.-Temperatur am Erdboden	°C	1951-1980	7.9	*8.3*	6.4		*-1.9*			
Heiße Tage (Max.: ≥ 30,0°C)	Tage	1909-1969	0.4	0.1	1		+1			
Sommertage (Max.: ≥ 25,0°C)	Tage	1909-1969	2.9	2.1	4		+2			
(Max.: ≥ 20,0°C)	Tage			10.8	21		+10			
Frosttage (Min.: < 0,0°C)	Tage	1909-1969	0	0	0		0			
(Min.: < -10,0°C)	Tage			0	0		0			
Eistage (Max.: < 0,0°C)	Tage	1909-1969	0	0	0		0			
Zahl d. Tage Min-Temp. am Erdb. < 0,0°C	Tage	1951-1980	0.3	0.1	0		-0			
Sonnenscheindauer	h	1951-1980	171.5	156.3	216.6		+60.3	139		
in % vom astronomisch möglichen	%		45.0	41.0	56.8		+15.8			
Zahl der sonnenscheinlosen Tage	Tage	1951-1980	2.2	2.4	0		-2			
Bewölkung (in Achtel)	/8	1909-1969	4.6	4.7	4.0		-0.7			
Heitere Tage (< 1,6 Achtel Bewölkung)	Tage	1909-1969	4.3	3.7	6		+2			
Trübe Tage (> 6,4 Achtel Bewölkung)	Tage	1909-1969	7.4	8.4	2		-6			
Dampfdruck	hPa	1909-1969	12.5	12.6	12.6		0.0			
Relative Luftfeuchtigkeit	%	1909-1969	79	80	75		-5			
Niederschlagshöhe	mm	1909-1969	45.7	45.5	30.5		-15.0	67		
Maximale Tagesmenge	mm	1909-1969	14.2	15.4	7.7	03.	-7.7		40.0	05.09.1931
Zahl der Tage mit Sturzregen	Tage			0.1	-		-			
Zahl der Tage mit ≥ 10,0 mm Niederschlag	Tage	1909-1969	1.0	1.0	0		-1			
Zahl der Tage mit ≥ 5,0 mm Niederschlag	Tage			2.7	3		+0			
Zahl der Tage mit ≥ 2,5 mm Niederschlag	Tage	1909-1969	5.5	5.2	6		+1			
Zahl der Tage mit ≥ 1,0 mm Niederschlag	Tage	1909-1969	8.4	8.6	7		-2			
Zahl der Tage mit ≥ 0,1 mm Niederschlag	Tage	1909-1969	12.6	14.4	10		-4			
Z.d.T.m. gefall. flüss. Niederschl. ≥ 0,1 mm	Tage			14.0	10		-4			
Z.d.T.m. flüss. u. fest. Niederschl. ≥ 0,1 mm	Tage			0.2	0		-0			
Z.d.T.m. gefall. fest. Niederschl. ≥ 0,1 mm	Tage			0.0	0		-0			
Z.d.T.m. abgesetzt. Niederschl. ≥ 0,1 mm	Tage			0.2	-		-			
Z.d.T.m. Hagel > 0,0 mm	Tage			0.1	0		-0			
Z.d.T.m. Graupel, Griesel o. Eisk. ≥ 0,0 mm	Tage			0.1	0		-0			
Zahl der Tage mit Reif oder Rauhreif	Tage	1964-1980	0.2	0.1	-		-			
Zahl der Tage mit Tau	Tage	1964-1980	27.4	27.2	-		-			
Zahl der Tage mit Nebel	Tage	1951-1980	2.4	1.8	0		-2			
Zahl der Tage mit Gewitter	Tage	1951-1980	2.4	2.4	1		-1			
Zahl der Tage mit Wetterleuchten	Tage	1951-1980	0.6	0.5	2		+1			
Z.d.T.m. Schneedecke ≥ 0 cm um 7:30 Uhr	Tage	1951-1980	0	0	0		0			
Z.d.T.m. Schneedecke ≥ 1 cm um 7:30 Uhr	Tage	1951-1980	0	0	0		0			
Z.d.T.m. Schneedecke ≥ 5 cm um 7:30 Uhr	Tage			0	0		0			
Z.d.T.m. Neuschnee ≥ 1 cm um 7:30 Uhr	Tage			0	0		0			
Summe der um 7:30 gem. Neuschneemenge	cm			.	0					
Max. Höhe der Schneedecke um 7:30 Uhr	cm			.						
Z.d.T.m. Glatteis durch gefrierenden Regen	Tage	1951-1980	0	0	0		0			
Z.d.T.m. Böen Windst. 6 Bft. (≥ 10,8 m/s)	Tage	1952-1980		9.8	12		+2			
Z.d.T.m. Böen Windst. 8 Bft. (≥ 17,2 m/s)	Tage	1952-1980		1.2	1		-0			
Maximale Windspitze	m/s			18.5	17.4	05.	-1.1		24.7*	9.9.1997
Heizgradsumme					30.7					
Kältesumme		1909-1969	0	0	0		0			

*) 1951-2008 +) 1881-2008

Manfred Wegener

Beiträge zur Berliner Wetterkarte
Klimatologische Mittelwerte von Berlin-Dahlem

60/10
KBD IX/10
ISSN 0177-3984
01.10.2009

Element	Maß-einheit	Beob-achtungs-periode	Vieljähr. Durch-schnitts-wert	Durch-schnitts-wert 1961-90	**September 2010**	Datum	Abweichung vom Durchschnitt 1961-90	in %	Bisherige Extreme 1909-2008	Datum
Luftdruck auf NN reduziert	hPa	1881-1970	1016.5	1016.7	1014.7		-2.0			
Höchster Luftdruck	hPa	1951-1980	1030.1	1030.0	1025.4	05.	-4.6		1038.5+	07.09.1953
Tiefster Luftdruck	hPa	1951-1980	1000.8	1000.4	999.4	25.	-1.0		987.2+	21.09.1990
Temperatur (stündl. Ablesung 01-24 MEZ)	°C	1955-1984	13.7	13.6	12.8		-0.8			
Temperatur (Klimamittel)	°C	1909-1969	13.9	13.5	12.8		-0.7			
Höchste Temperatur	°C	1909-1969	27.0	26.1	24.4	24.	-1.7		34.2	03.09.1911<br>12.9.1919
Mittlere Maximum-Temperatur	°C	1909-1969	19.3	18.7	17.4		-1.3			
Tiefste Temperatur	°C	1909-1969	3.4	4.0	2.5	30.	-1.5		-0.5	21.09.1915
Mittlere Minimum-Temperatur	°C	1909-1969	9.4	9.4	8.6		-0.8			
Tiefste Temperatur am Erdboden	°C	1951-1980	1.8	2.6	-0.2	30.	-2.8		-1.9*	21.09.1997
Mittlere Min.-Temperatur am Erdboden	°C	1951-1980	7.9	*8.3*	6.1		*-2.2*			
Heiße Tage (Max.: ≥ 30,0°C)	Tage	1909-1969	0.4	0.1	0		-0			
Sommertage (Max.: ≥ 25,0°C)	Tage	1909-1969	2.9	2.1	0		-2			
(Max.: ≥ 20,0°C)	Tage			10.8	6		-5			
Frosttage (Min.: < 0,0°C)	Tage	1909-1969	0	0	0		0			
(Min.: ≤ -10,0°C)	Tage			0	0		0			
Eistage (Max.: < 0,0°C)	Tage	1909-1969	0	0	0		0			
Zahl d. Tage Min-Temp. am Erdb. < 0,0°C	Tage	1951-1980	0.3	0.1	1		+1			
Sonnenscheindauer	h	1951-1980	171.5	156.3	142.9		-13.4	91		
in % vom astronomisch möglichen	%		45.0	41.0	37.5		-3.5			
Zahl der sonnenscheinlosen Tage	Tage	1951-1980	2.2	2.4	6		+4			
Bewölkung (in Achtel)	/8	1909-1969	4.6	4.7	5.1		+0.4			
Heitere Tage (< 1,6 Achtel Bewölkung)	Tage	1909-1969	4.3	3.7	1		-3			
Trübe Tage (> 6,4 Achtel Bewölkung)	Tage	1909-1969	7.4	8.4	9		+1			
Dampfdruck	hPa	1909-1969	12.5	12.6	11.8		-0.8			
Relative Luftfeuchtigkeit	%	1909-1969	79	80	81		+1			
Niederschlagshöhe	mm	1909-1969	45.7	45.5	89.7		+44.2	197		
Maximale Tagesmenge	mm	1909-1969	14.2	15.4	29.8	26.	+14.4		40.0	05.09.1931
Zahl der Tage mit Sturzregen	Tage			0.1	-		-			
Zahl der Tage mit ≥ 10,0 mm Niederschlag	Tage	1909-1969	1.0	1.0	3		+2			
Zahl der Tage mit ≥ 5,0 mm Niederschlag	Tage			2.7	4		+1			
Zahl der Tage mit ≥ 2,5 mm Niederschlag	Tage	1909-1969	5.5	5.2	6		+1			
Zahl der Tage mit ≥ 1,0 mm Niederschlag	Tage	1909-1969	8.4	8.6	9		+0			
Zahl der Tage mit ≥ 0,1 mm Niederschlag	Tage	1909-1969	12.6	14.4	16		+2			
Z.d.T.m. gefall. flüss. Niederschl. ≥ 0,1 mm	Tage			14.0	16		+2			
Z.d.T.m. flüss. u. fest. Niederschl. ≥ 0,1 mm	Tage			0.2	0		-0			
Z.d.T.m. gefall. fest. Niederschl. ≥ 0,1 mm	Tage			0.0	0		-0			
Z.d.T.m. abgesetzt. Niederschl. ≥ 0,1 mm	Tage			0.2	-		-			
Z.d.T.m. Hagel ≥ 0,0 mm	Tage			0.1	0		-0			
Z.d.T.m. Graupel, Griesel o. Eisk. ≥ 0,0 mm	Tage			0.1	0		-0			
Zahl der Tage mit Reif oder Rauhreif	Tage	1964-1980	0.2	0.1	-		-			
Zahl der Tage mit Tau	Tage	1964-1980	27.4	27.2	-		-			
Zahl der Tage mit Nebel	Tage	1951-1980	2.4	1.8	4		+2			
Zahl der Tage mit Gewitter	Tage	1951-1980	2.4	2.4	1		-1			
Zahl der Tage mit Wetterleuchten	Tage	1951-1980	0.6	0.5	2		+1			
Z.d.T.m. Schneedecke ≥ 0 cm um 7:30 Uhr	Tage	1951-1980	0	0	0		0			
Z.d.T.m. Schneedecke ≥ 1 cm um 7:30 Uhr	Tage	1951-1980	0	0	0		0			
Z.d.T.m. Schneedecke ≥ 5 cm um 7:30 Uhr	Tage			0	0		0			
Z.d.T.m. Neuschnee ≥ 1 cm um 7:30 Uhr	Tage			0	0		0			
Summe der um 7:30 gem. Neuschneemenge	cm			.	0					
Max. Höhe der Schneedecke um 7:30 Uhr	cm			.						
Z.d.T.m. Glatteis durch gefrierenden Regen	Tage	1951-1980	0	0	0		0			
Z.d.T.m. Böen Windst. 6 Bft. (≥ 10,8 m/s)	Tage	1952-1980		9.8	14		+4			
Z.d.T.m. Böen Windst. 8 Bft. (≥ 17,2 m/s)	Tage	1952-1980		1.2	1		-0			
Maximale Windspitze	m/s			18.5	21.7	15.	+3.2		24.7*	9.9.1997
Heizgradsumme					122.6					
Kältesumme		1909-1969	0	0	0		0			

*) 1951-2008 +) 1881-2008

Manfred Wegener
