# Supplementary material for: To mulch or not to mulch? Effects of gravel mulch toppings on plant establishment and development in ornamental prairie plantings
Source: PLoS One. 2017 Feb 6;12(2):e0171533. doi: 10.1371/journal.pone.0171533 (PMC5293235; doi:10.1371/journal.pone.0171533)
Supplement: S2 File — (PDF) [file pone.0171533.s005.pdf]

Beiträge zur Berliner Wetterkarte

Klimatologische Mittelwerte von Berlin-Dahlem

09/11 ISSN 0177-3984
KBD 01/11 01.02.2011

Element	Maßeinheit	Beobachtungsperiode	Vieljähr. Durchschnittswert	Durchschnittswert 1961-90	**JANUAR 2011**	Datum	Abweichung vom Durchschnitt 1961-90	in %	Bisherige Extreme 1909-2010	Datum
Luftdruck auf NN reduziert	hPa	1881-1970	1016.8	1016.2	1017.8		+1.6			
Höchster Luftdruck	hPa	1951-1980	1036.8	1036.7	1033.2	21.	-3.5		1057.8+	23.01.1907
Tiefster Luftdruck	hPa	1951-1980	989.7	989.9	998.9	07.	+9.0		966.6+	17.01.1955
Temperatur (stündl. Ablesung 01-24 MEZ)	°C	1955-1984	-0.5	-0.5	1.2		+1.7			
Temperatur (Klimamittel)	°C	1909-1969	-0.4	-0.4	1.2		+1.6			
Höchste Temperatur	°C	1909-1969	8.8	8.3	10.4	14.	+2.1		15.2	10.01.1991<br>28.01.2002
Mittlere Maximum-Temperatur	°C	1909-1969	1.9	1.8	3.6		+1.8			
Tiefste Temperatur	°C	1909-1969	-11.8	-11.4	-8.9	05.	+2.5		-21.0	26.01.1942
Mittlere Minimum-Temperatur	°C	1909-1969	-3.1	-2.9	-1.2		+1.7			
Tiefste Temperatur am Erdboden	°C	1951-1980	-14.5	*-14.0*	-11.7	05.	*+2.3*		-26.6*	31.01.1963
Mittlere Min.-Temperatur am Erdboden	°C	1951-1980	-4.2	*-4.1*	-3.3		*+0.8*			
Heiße Tage (Max.: ≥ 30,0°C)	Tage	1909-1969	0	0	0		0			
Sommertage (Max.: ≥ 25,0°C)	Tage	1909-1969	0	0	0		0			
(Max.: ≥ 20,0°C)	Tage			0	0		0			
Frosttage (Min.: < 0,0°C)	Tage	1909-1969	20.5	19.9	18		-2			
(Min.: ≤ -10,0°C)	Tage			4.0	0		-4			
Eistage (Max.: < 0,0°C)	Tage	1909-1969	9.4	9.2	7		-2			
Zahl d. Tage Min-Temp. am Erdb. < 0,0°C	Tage	1951-1980	24.1	23.1	25		+2			
Sonnenscheindauer	h	1951-1980	48.0	45.4	48.5		+3.1	107		
in % vom astronomisch möglichen	%		18.7	17.7	18.9		+1.2			
Zahl der sonnenscheinlosen Tage	Tage	1951-1980	14.4	13.9	14		+0			
Bewölkung (in Achtel)	/8	1909-1969	6.0	6.0	6.1		+0.1			
Heitere Tage (< 1,6 Achtel Bewölkung)	Tage	1909-1969	2.1	1.9	2		+0			
Trübe Tage (> 6,4 Achtel Bewölkung)	Tage	1909-1969	17.4	17.4	18		+1			
Dampfdruck	hPa	1909-1969	5.3	5.4	6.0		+0.6			
Relative Luftfeuchtigkeit	%	1909-1969	85	85	87		+2			
Niederschlagshöhe	mm	1909-1969	46.6	43.2	34.3		-8.9	79		
Maximale Tagesmenge	mm	1909-1969	11.2	9.3	7.4	14.	-1.9		45.0	18.01.2007
Zahl der Tage mit Sturzregen	Tage			0	-		-			
Zahl der Tage mit ≥ 10,0 mm Niederschlag	Tage	1909-1969	0.8	0.4	0		-0			
Zahl der Tage mit ≥ 5,0 mm Niederschlag	Tage			2.7	3		+0			
Zahl der Tage mit ≥ 2,5 mm Niederschlag	Tage	1909-1969	6.4	6.4	5		-1			
Zahl der Tage mit ≥ 1,0 mm Niederschlag	Tage	1909-1969	10.6	10.3	8		-2			
Zahl der Tage mit ≥ 0,1 mm Niederschlag	Tage	1909-1969	17.5	17.8	17		-1			
Z.d.T.m. gefall. flüss. Niederschl. ≥ 0,1 mm	Tage			6.9	10		+3			
Z.d.T.m. flüss. u. fest. Niederschl. ≥ 0,1 mm	Tage			6.4	5		-1			
Z.d.T.m. gefall. fest. Niederschl. ≥ 0,1 mm	Tage			4.5	2		-3			
Z.d.T.m. abgesetzt. Niederschl. ≥ 0,1 mm	Tage			0.1	-		-			
Z.d.T.m. Hagel ≥ 0,0 mm	Tage			0.1	0		-0			
Z.d.T.m. Graupel, Griesel o. Eisk. ≥ 0,0 mm	Tage		5.0	6.3	6		-0			
Zahl der Tage mit Reif oder Rauhreif	Tage	1964-1980	15.1	14.4	-		-			
Zahl der Tage mit Tau	Tage	1964-1980	4.8	5.1	-		-			
Zahl der Tage mit Nebel	Tage	1951-1980	5.4	5.3	1		-4			
Zahl der Tage mit Gewitter	Tage	1951-1980	0.3	0.5	0		-1			
Zahl der Tage mit Wetterleuchten	Tage	1951-1980	0.1	0.1	0		-0			
Z.d.T.m. Schneedecke ≥ 0 cm um 7:30 Uhr	Tage	1951-1980	16.1	16.3	20		+4			
Z.d.T.m. Schneedecke ≥ 1 cm um 7:30 Uhr	Tage	1951-1980	15.1	15.3	19		+4			
Z.d.T.m. Schneedecke ≥ 5 cm um 7:30 Uhr	Tage			9.4	14		+5			
Z.d.T.m. Neuschnee ≥ 1 cm um 7:30 Uhr	Tage			4.8	3		-2			
Summe der um 7:30 gem. Neuschneemenge	cm				3					
Max. Höhe der Schneedecke um 7:30 Uhr	cm			9.5	32	01.	+23		31	06.01.1970
Z.d.T.m. Glatteis durch gefrierenden Regen	Tage	1951-1980	2.9	2.8	1		-2			
Z.d.T.m. Böen Windst. 6 Bft. (≥ 10,8 m/s)	Tage	1952-1980		12.0	12		-0			
Z.d.T.m. Böen Windst. 8 Bft. (≥ 17,2 m/s)	Tage	1952-1980		3.0	1		-2			
Maximale Windspitze	m/s			21.2	18.6	01.	-2.6		34.5*	18.1.2007
Heizgradsumme					590.1					
Kältesumme		1909-1969	66.2	66.6	30.0		-36.6			

*) 1951-2010 +) 1881-2010

Manfred Wegener

Beiträge zur Berliner Wetterkarte

Klimatologische Mittelwerte von Berlin-Dahlem

14/11 ISSN 0177-3984
KBD II/11 01.03.2011

Element	Maß-einheit	Beob-achtungs-periode	Vieljähr. Durch-schnitts-wert	Durch-schnitts-wert 1961-90	**Februar 2011**	Datum	Abweichung vom Durchschnitt 1961-90	in %	Bisherige Extreme 1909-2010	Datum
Luftdruck auf NN reduziert	hPa	1881-1970	1016.1	1016.2	1019.6		+3.4			
Höchster Luftdruck	hPa	1951-1980	1035.7	1036.2	1035.3	25.	-0.9		1048.0+	16.02.1959
Tiefster Luftdruck	hPa	1951-1980	990.1	989.6	1005.6	16.	+16.0		965.2+	26.02.1989
Temperatur (stündl. Ablesung 01-24 MEZ)	°C	1955-1984	0.2	0.5	-0.3		-0.8			
Temperatur (Klimamittel)	°C	1909-1969	0.1	0.6	-0.3		-0.9			
Höchste Temperatur	°C	1909-1969	10.4	10.0	10.5	05./28.	+0.5		18.6	21.02.1990
Mittlere Maximum-Temperatur	°C	1909-1969	3.1	3.5	2.8		-0.7			
Tiefste Temperatur	°C	1909-1969	-11.4	-9.4	-12.8	23.	-3.4		-26.0	11.02.1929
Mittlere Minimum-Temperatur	°C	1909-1969	-2.9	-2.2	-3.2		-1.0			
Tiefste Temperatur am Erdboden	°C	1951-1980	-14.1	*-12.3*	-14.7	23.	*-2.4*		-29.2*	09.02.1956
Mittlere Min.-Temperatur am Erdboden	°C	1951-1980	-4.1	*-3.6*	-4.7		*-1.1*			
Heiße Tage (Max.: ≥ 30,0°C)	Tage	1909-1969	0	0	0		0			
Sommertage (Max.: ≥ 25,0°C)	Tage	1909-1969	0	0	0		0			
(Max.: ≥ 20,0°C)	Tage			0	0		0			
Frosttage (Min.: < 0,0°C)	Tage	1909-1969	18.5	17.6	22		+4			
(Min.: ≤ -10,0°C)	Tage			2.1	4		+2			
Eistage (Max.: < 0,0°C)	Tage	1909-1969	6.8	5.9	8		+2			
Zahl d. Tage Min-Temp. am Erdb. < 0,0°C	Tage	1951-1980	21.5	21.1	26		+5			
Sonnenscheindauer	h	1951-1980	69.3	72.3	100.3		+28.0	139		
in % vom astronomisch möglichen	%		25.1	26.2	36.3		+10.1			
Zahl der sonnenscheinlosen Tage	Tage	1951-1980	10.0	10.0	10		-0			
Bewölkung (in Achtel)	/8	1909-1969	5.8	5.6	5.3		-0.3			
Heitere Tage (< 1,6 Achtel Bewölkung)	Tage	1909-1969	2.2	3.1	4		+1			
Trübe Tage (> 6,4 Achtel Bewölkung)	Tage	1909-1969	14.3	14.1	12		-2			
Dampfdruck	hPa	1909-1969	5.3	5.5	4.5		-1.0			
Relative Luftfeuchtigkeit	%	1909-1969	82	82	70		-12			
Niederschlagshöhe	mm	1909-1969	36.8	36.6	24.4		-12.2	67		
Maximale Tagesmenge	mm	1909-1969	9.6	9.9	13.7	05.	+3.8		24.2	06.02.1974
Zahl der Tage mit Sturzregen	Tage			0	-		-			
Zahl der Tage mit ≥ 10,0 mm Niederschlag	Tage	1909-1969	0.7	0.6	1		+0			
Zahl der Tage mit ≥ 5,0 mm Niederschlag	Tage			2.1	2		-0			
Zahl der Tage mit ≥ 2,5 mm Niederschlag	Tage	1909-1969	4.6	4.7	3		-2			
Zahl der Tage mit ≥ 1,0 mm Niederschlag	Tage	1909-1969	8.5	8.5	4		-5			
Zahl der Tage mit ≥ 0,1 mm Niederschlag	Tage	1909-1969	14.1	14.3	7		-7			
Z.d.T.m. gefall. flüss. Niederschl. ≥ 0,1 mm	Tage			4.8	5		+0			
Z.d.T.m. flüss. u. fest. Niederschl. ≥ 0,1 mm	Tage			5.8	2		-4			
Z.d.T.m. gefall. fest. Niederschl. ≥ 0,1 mm	Tage			3.6	0		-4			
Z.d.T.m. abgesetzt. Niederschl. ≥ 0,1 mm	Tage			0.0	-		-			
Z.d.T.m. Hagel ≥ 0,0 mm	Tage			0.2	0		-0			
Z.d.T.m. Graupel, Griesel o. Eisk. ≥ 0,0 mm	Tage			6.1	8		+2			
Zahl der Tage mit Reif oder Rauhreif	Tage	1964-1980	13.8	13.6	-		-			
Zahl der Tage mit Tau	Tage	1964-1980	5.4	5.6	-		-			
Zahl der Tage mit Nebel	Tage	1951-1980	4.8	4.2	0		-4			
Zahl der Tage mit Gewitter	Tage	1951-1980	0.4	0.7	0		-1			
Zahl der Tage mit Wetterleuchten	Tage	1951-1980	0.1	0.1	0		-0			
Z.d.T.m. Schneedecke ≥ 0 cm um 7:30 Uhr	Tage	1951-1980	13.0	12.1	3		-9			
Z.d.T.m. Schneedecke ≥ 1 cm um 7:30 Uhr	Tage	1951-1980	12.0	10.8	0		-11			
Z.d.T.m. Schneedecke ≥ 5 cm um 7:30 Uhr	Tage			6.5	0		-7			
Z.d.T.m. Neuschnee ≥ 1 cm um 7:30 Uhr	Tage			3.7	0		-4			
Summe der um 7:30 gem. Neuschneemenge	cm									
Max. Höhe der Schneedecke um 7:30 Uhr	cm			9.9			-10		44	16.02.1940
Z.d.T.m. Glatteis durch gefrierenden Regen	Tage	1951-1980	1.1	1.3	0		-1			
Z.d.T.m. Böen Windst. 6 Bft. (≥ 10,8 m/s)	Tage	1952-1980		11.0	12		+1			
Z.d.T.m. Böen Windst. 8 Bft. (≥ 17,2 m/s)	Tage	1952-1980		2.1	3		+1			
Maximale Windspitze	m/s			20.2	23.8	05.	+3.6		35.5*	21.2.1967
Heizgradsumme					594.4					
Kältesumme		1909-1969	52.1	38.7	55.9		+17.2			

*) 1951-2010 +) 1881-2010

Manfred Wegener

Beiträge zur Berliner Wetterkarte

Klimatologische Mittelwerte von Berlin-Dahlem

22/11 ISSN 0177-3984
KBD III/11 01.04.2011

Element	Maß-einheit	Beob-achtungs-periode	Vieljähr. Durch-schnitts-wert	Durch-schnitts-wert 1961-90	**März 2011**	Datum	Abweichung vom Durchschnitt 1961-90	in %	Bisherige Extreme 1909-2011	Datum
Luftdruck auf NN reduziert	hPa	1881-1970	1014.6	1015.1	1022.7		+7.6			
Höchster Luftdruck	hPa	1951-1980	1033.3	1033.2	1039.5	07.	+6.3		1044.7*	15.03.2003
Tiefster Luftdruck	hPa	1951-1980	993.6	990.6	1005.9	13.	+15.3		972.7*	01.03.1990
Temperatur (stündl. Ablesung 01-24 MEZ)	°C	1955-1984	3.8	4.0	4.8		+0.8			
Temperatur (Klimamittel)	°C	1909-1969	3.7	4.0	4.8		+0.8			
Höchste Temperatur	°C	1909-1969	16.4	16.6	16.8	30.	+0.2		25.1	30.03.1968
Mittlere Maximum-Temperatur	°C	1909-1969	7.8	7.9	10.1		+2.2			
Tiefste Temperatur	°C	1909-1969	-7.0	-6.0	-7.0	07.	-1.0		-16.5	06.03.1942
Mittlere Minimum-Temperatur	°C	1909-1969	-0.2	0.5	-0.2		-0.7			
Tiefste Temperatur am Erdboden	°C	1951-1980	-9.3	-8.8	-11.0	07.	-2.2		-21.6*	08.03.1965
Mittlere Min.-Temperatur am Erdboden	°C	1951-1980	-1.5	*-1.1*	-3.6		*-2.5*			
Heiße Tage (Max.: ≥ 30,0°C)	Tage	1909-1969	0	0	0		0			
Sommertage (Max.: ≥ 25,0°C)	Tage	1909-1969	0.0	0.0	0		-0			
(Max.: ≥ 20,0°C)	Tage			0.3	0		-0			
Frosttage (Min.: < 0,0°C)	Tage	1909-1969	15.1	12.6	18		+5			
(Min.: ≤ -10,0°C)	Tage			0.3	0		-0			
Eistage (Max.: < 0,0°C)	Tage	1909-1969	1.7	1.3	0		-1			
Zahl d. Tage Min-Temp. am Erdb. < 0,0°C	Tage	1951-1980	19.5	18.1	24		+6			
Sonnenscheindauer	h	1951-1980	134.2	122.1	198.5		+76.4	163		
in % vom astronomisch möglichen	%		36.6	33.3	54.1		+20.8			
Zahl der sonnenscheinlosen Tage	Tage	1951-1980	5.8	5.9	5		-1			
Bewölkung (in Achtel)	/8	1909-1969	5.1	5.3	3.8		-1.5			
Heitere Tage (< 1,6 Achtel Bewölkung)	Tage	1909-1969	3.8	3.3	8		+5			
Trübe Tage (> 6,4 Achtel Bewölkung)	Tage	1909-1969	11.7	13.0	7		-6			
Dampfdruck	hPa	1909-1969	6.1	6.3	5.9		-0.4			
Relative Luftfeuchtigkeit	%	1909-1969	76	75	69		-6			
Niederschlagshöhe	mm	1909-1969	33.9	37.5	16.5		-21.0	44		
Maximale Tagesmenge	mm	1909-1969	10.2	9.4	9.7	17.	+0.3		30.7	06.03.1915
Zahl der Tage mit Sturzregen	Tage			0	-		-			
Zahl der Tage mit ≥ 10,0 mm Niederschlag	Tage	1909-1969	0.5	0.6	0		-1			
Zahl der Tage mit ≥ 5,0 mm Niederschlag	Tage			2.1	1		-1			
Zahl der Tage mit ≥ 2,5 mm Niederschlag	Tage	1909-1969	4.4	5.2	2		-3			
Zahl der Tage mit ≥ 1,0 mm Niederschlag	Tage	1909-1969	7.8	8.4	4		-4			
Zahl der Tage mit ≥ 0,1 mm Niederschlag	Tage	1909-1969	13.2	15.5	7		-9			
Z.d.T.m. gefall. flüss. Niederschl. ≥ 0,1 mm	Tage			7.7	7		-1			
Z.d.T.m. flüss. u. fest. Niederschl. ≥ 0,1 mm	Tage			5.1	0		-5			
Z.d.T.m. gefall. fest. Niederschl. ≥ 0,1 mm	Tage			2.5	0		-3			
Z.d.T.m. abgesetzt. Niederschl. ≥ 0,1 mm	Tage			0.1	-		-			
Z.d.T.m. Hagel ≥ 0,0 mm	Tage			0.2	0		-0			
Z.d.T.m. Graupel, Griesel o. Eisk. ≥ 0,0 mm	Tage			4.5	0		-5			
Zahl der Tage mit Reif oder Rauhreif	Tage	1964-1980	12.0	11.2	-		-			
Zahl der Tage mit Tau	Tage	1964-1980	10.1	10.5	-		-			
Zahl der Tage mit Nebel	Tage	1951-1980	2.0	1.6	1		-1			
Zahl der Tage mit Gewitter	Tage	1951-1980	0.9	0.9	0		-1			
Zahl der Tage mit Wetterleuchten	Tage	1951-1980	0.1	0.0	0		-0			
Z.d.T.m. Schneedecke ≥ 0 cm um 7:30 Uhr	Tage	1951-1980	6.9	6.8	0		-7			
Z.d.T.m. Schneedecke ≥ 1 cm um 7:30 Uhr	Tage	1951-1980	5.7	5.0	0		-5			
Z.d.T.m. Schneedecke ≥ 5 cm um 7:30 Uhr	Tage			3.1	0		-3			
Z.d.T.m. Neuschnee ≥ 1 cm um 7:30 Uhr	Tage			1.9	0		-2			
Summe der um 7:30 gem. Neuschneemenge	cm				0					
Max. Höhe der Schneedecke um 7:30 Uhr	cm			7.3			-7		49	06.03.1970
Z.d.T.m. Glatteis durch gefrierenden Regen	Tage	1951-1980	0.3	0.1	0		-0			
Z.d.T.m. Böen Windst. 6 Bft. (≥ 10,8 m/s)	Tage	1952-1980		15.4	13		-2			
Z.d.T.m. Böen Windst. 8 Bft. (≥ 17,2 m/s)	Tage	1952-1980		3.4	2		-1			
Maximale Windspitze	m/s			21.6	18.6	24.	-3.0		30.9*	28.3.1997
Heizgradsumme					482.2					
Kältesumme		1909-1969	12.1	10.3	1.3		-9.0			

*) 1951-2010 +) 1881-2010

Manfred Wegener

Beiträge zur Berliner Wetterkarte

Klimatologische Mittelwerte von Berlin-Dahlem

28/11 ISSN 0177-3984
KBD V/11 03.05.2010

Element	Maß-einheit	Beob-achtungs-periode	Vieljähr. Durch-schnitts-wert	Durch-schnitts-wert 1961-90	**April 2011**	Datum	Abweichung vom Durchschnitt 1961-90	in %	Bisherige Extreme 1909-2010	Datum
Luftdruck auf NN reduziert	hPa	1881-1970	1013.5	1013.8	1018.3		+4.5			
Höchster Luftdruck	hPa	1951-1980	1030.0	1029.7	1025.6	17.	-4.1		1037.5*	21.04.2002
Tiefster Luftdruck	hPa	1951-1980	995.4	995.6	1008.7	12.	+13.1		983.5*	07.04.1959
Temperatur (stündl. Ablesung 01-24 MEZ)	°C	1955-1984	8.1	8.3	12.5		+4.2			
Temperatur (Klimamittel)	°C	1909-1969	8.6	8.4	12.5		+4.1			
Höchste Temperatur	°C	1909-1969	22.6	22.6	24.9	03.	+2.3		30.9	22.04.1968
Mittlere Maximum-Temperatur	°C	1909-1969	13.4	13.1	18.2		+5.1			
Tiefste Temperatur	°C	1909-1969	-2.3	-2.2	0.9	16.	+3.1		-6.7	04.04.1929
Mittlere Minimum-Temperatur	°C	1909-1969	3.8	3.9	7.0		+3.1			
Tiefste Temperatur am Erdboden	°C	1951-1980	-4.5	-4.1	-2.3	16.	+1.8		-8.1*	08.04.1956
Mittlere Min.-Temperatur am Erdboden	°C	1951-1980	2.0	*-2.2*	3.3		*+5.5*			
Heiße Tage (Max.: ≥ 30,0°C)	Tage	1909-1969	0.1	0.1	0		-0			
Sommertage (Max.: ≥ 25,0°C)	Tage	1909-1969	0.6	0.4	0		-0			
(Max.: ≥ 20,0°C)	Tage			2.6	14		+11			
Frosttage (Min.: < 0,0°C)	Tage	1909-1969	4.1	3.4	0		-3			
(Min.: ≤ -10,0°C)	Tage			0	0		0			
Eistage (Max.: < 0,0°C)	Tage	1909-1969	0	0	0		0			
Zahl d. Tage Min-Temp. am Erdb. < 0,0°C	Tage	1951-1980	9.2	8.8	5		-4			
Sonnenscheindauer	h	1951-1980	165.6	157.7	216.7		+59.0	137		
in % vom astronomisch möglichen	%		39.8	37.9	52.1		+14.2			
Zahl der sonnenscheinlosen Tage	Tage	1951-1980	3.5	3.8	2		-2			
Bewölkung (in Achtel)	/8	1909-1969	5.0	5.0	4.1		-0.9			
Heitere Tage (< 1,6 Achtel Bewölkung)	Tage	1909-1969	3.2	3.2	9		+6			
Trübe Tage (> 6,4 Achtel Bewölkung)	Tage	1909-1969	9.4	10.3	7		-3			
Dampfdruck	hPa	1909-1969	7.7	7.5	9.0		+1.5			
Relative Luftfeuchtigkeit	%	1909-1969	70	69	65		-4			
Niederschlagshöhe	mm	1909-1969	42.8	42.2	31.4		-10.8	74		
Maximale Tagesmenge	mm	1909-1969	10.7	10.4	9.6	13.	-0.8		31.9	12.04.1949
Zahl der Tage mit Sturzregen	Tage			0	-		-			
Zahl der Tage mit ≥ 10,0 mm Niederschlag	Tage	1909-1969	0.8	0.9	0		-1			
Zahl der Tage mit ≥ 5,0 mm Niederschlag	Tage			2.7	3		+0			
Zahl der Tage mit ≥ 2,5 mm Niederschlag	Tage	1909-1969	5.8	5.4	4		-1			
Zahl der Tage mit ≥ 1,0 mm Niederschlag	Tage	1909-1969	9.1	8.9	7		-2			
Zahl der Tage mit ≥ 0,1 mm Niederschlag	Tage	1909-1969	13.8	14.2	8		-6			
Z.d.T.m. gefall. flüss. Niederschl. ≥ 0,1 mm	Tage			10.2	6		-4			
Z.d.T.m. flüss. u. fest. Niederschl. ≥ 0,1 mm	Tage			3.7	2		-2			
Z.d.T.m. gefall. fest. Niederschl. ≥ 0,1 mm	Tage			0.3	0		-0			
Z.d.T.m. abgesetzt. Niederschl. ≥ 0,1 mm	Tage			0.0	-		-			
Z.d.T.m. Hagel ≥ 0,0 mm	Tage			0.3	0		-0			
Z.d.T.m. Graupel, Griesel o. Eisk. ≥ 0,0 mm	Tage			2.5	2		-1			
Zahl der Tage mit Reif oder Rauhreif	Tage	1964-1980	6.0	5.1	-		-			
Zahl der Tage mit Tau	Tage	1964-1980	19.9	19.3	-		-			
Zahl der Tage mit Nebel	Tage	1951-1980	1.4	1.0	0		-1			
Zahl der Tage mit Gewitter	Tage	1951-1980	1.9	2.2	2		-0			
Zahl der Tage mit Wetterleuchten	Tage	1951-1980	0.3	0.3	0		-0			
Z.d.T.m. Schneedecke ≥ 0 cm um 7:30 Uhr	Tage	1951-1980	0.5	0.4	0		-0			
Z.d.T.m. Schneedecke ≥ 1 cm um 7:30 Uhr	Tage	1951-1980	0.4	0.3	0		-0			
Z.d.T.m. Schneedecke ≥ 5 cm um 7:30 Uhr	Tage			0.1	0		-0			
Z.d.T.m. Neuschnee ≥ 1 cm um 7:30 Uhr	Tage			0.3	0		-0			
Summe der um 7:30 gem. Neuschneemenge	cm									
Max. Höhe der Schneedecke um 7:30 Uhr	cm			1.1			-1		14	11.04.1977
Z.d.T.m. Glatteis durch gefrierenden Regen	Tage	1951-1980	0	0	0		0			
Z.d.T.m. Böen Windst. 6 Bft. (≥ 10,8 m/s)	Tage	1952-1980		13.4	18		+5			
Z.d.T.m. Böen Windst. 8 Bft. (≥ 17,2 m/s)	Tage	1952-1980		1.9	5		+3			
Maximale Windspitze	m/s			20.0	27.5	08.	+7.5		25.8*	19.4.1980
Heizgradsumme					233.7					
Kältesumme		1909-1969	0.3	0.1	0.0		-0.1			

*) 1951-2010 +) 1881-2010

Manfred Wegener

Beiträge zur Berliner Wetterkarte

Klimatologische Mittelwerte von Berlin-Dahlem

32/11 ISSN 0177-3984
KBD V/11 01.06.2011

Element	Maßeinheit	Beobachtungsperiode	Vieljähr. Durchschnittswert	Durchschnittswert 1961-90	**MAI 2011**	Datum	Abweichung vom Durchschnitt 1961-90	in %	Bisherige Extreme 1909-2010	Datum
Luftdruck auf NN reduziert	hPa	1881-1970	1015.1	1015.2	1018.6		+3.4			
Höchster Luftdruck	hPa	1951-1980	1028.3	1028.1	1029.9	09.	+1.8		1037.5*	02.05.1990
Tiefster Luftdruck	hPa	1951-1980	1000.7	999.5	1007.0	26.	+7.5		984.4*	06.05.1997
Temperatur (stündl. Ablesung 01-24 MEZ)	°C	1955-1984	13.1	13.4	14.5		+1.1			
Temperatur (Klimamittel)	°C	1909-1969	13.6	13.5	14.5		+1.0			
Höchste Temperatur	°C	1909-1969	27.1	27.0	32.7	31.	+5.7		33.3	28.05.2005
Mittlere Maximum-Temperatur	°C	1909-1969	18.7	18.6	21.3		+2.7			
Tiefste Temperatur	°C	1909-1969	1.1	1.7	-0.8	05.	-2.5		-2.9	09.05.1941
Mittlere Minimum-Temperatur	°C	1909-1969	8.0	8.2	7.8		-0.4			
Tiefste Temperatur am Erdboden	°C	1951-1980	-0.4	0.8	-4.7	05.	-5.5		-4.1*	12.05.1978
Mittlere Min.-Temperatur am Erdboden	°C	1951-1980	6.4	*6.7*	3.1		*-3.6*			
Heiße Tage (Max.: ≥ 30,0°C)	Tage	1909-1969	0.4	0.2	2		+2			
Sommertage (Max.: ≥ 25,0°C)	Tage	1909-1969	3.4	3.3	7		+4			
(Max.: ≥ 20,0°C)	Tage			11.9	20		+8			
Frosttage (Min.: < 0,0°C)	Tage	1909-1969	0.3	0.2	1		+1			
(Min.: ≤ -10,0°C)	Tage			0	0		0			
Eistage (Max.: < 0,0°C)	Tage	1909-1969	0	0	0		0			
Zahl d. Tage Min-Temp. am Erdb. < 0,0°C	Tage	1951-1980	1.6	1.0	6		+5			
Sonnenscheindauer	h	1951-1980	221.4	221.6	286.6		+65.0	129		
in % vom astronomisch möglichen	%		45.4	45.5	58.8		+13.3			
Zahl der sonnenscheinlosen Tage	Tage	1951-1980	2.0	2.1	0		-2			
Bewölkung (in Achtel)	/8	1909-1969	4.9	4.7	3.8		-0.9			
Heitere Tage (< 1,6 Achtel Bewölkung)	Tage	1909-1969	3.6	3.6	7		+3			
Trübe Tage (> 6,4 Achtel Bewölkung)	Tage	1909-1969	8.8	8.3	2		-6			
Dampfdruck	hPa	1909-1969	10.4	10.3	10.1		-0.2			
Relative Luftfeuchtigkeit	%	1909-1969	67	67	63		-4			
Niederschlagshöhe	mm	1909-1969	49.0	55.3	30.1		-25.2	54		
Maximale Tagesmenge	mm	1909-1969	15.9	15.3	12.4	22.	-2.9		50.3	03.05.1996
Zahl der Tage mit Sturzregen	Tage			0.1	-		-			
Zahl der Tage mit ≥ 10,0 mm Niederschlag	Tage	1909-1969	1.3	1.4	1		-0			
Zahl der Tage mit ≥ 5,0 mm Niederschlag	Tage			4.0	2		-2			
Zahl der Tage mit ≥ 2,5 mm Niederschlag	Tage	1909-1969	5.4	6.4	3		-3			
Zahl der Tage mit ≥ 1,0 mm Niederschlag	Tage	1909-1969	8.5	9.6	8		-2			
Zahl der Tage mit ≥ 0,1 mm Niederschlag	Tage	1909-1969	12.6	14.0	11		-3			
Z.d.T.m. gefall. flüss. Niederschl. ≥ 0,1 mm	Tage			13.0	10		-3			
Z.d.T.m. flüss. u. fest. Niederschl. ≥ 0,1 mm	Tage			1.0	1		0			
Z.d.T.m. gefall. fest. Niederschl. ≥ 0,1 mm	Tage			0	0		0			
Z.d.T.m. abgesetzt. Niederschl. ≥ 0,1 mm	Tage			0.0	-		-			
Z.d.T.m. Hagel ≥ 0,0 mm	Tage			0.4	0		-0			
Z.d.T.m. Graupel, Griesel o. Eisk. ≥ 0,0 mm	Tage			0.5	1		+0			
Zahl der Tage mit Reif oder Rauhreif	Tage	1964-1980	0	0	-		-			
Zahl der Tage mit Tau	Tage	1964-1980	23.2	23.3	-		-			
Zahl der Tage mit Nebel	Tage	1951-1980	0.7	0.6	0		-1			
Zahl der Tage mit Gewitter	Tage	1951-1980	4.4	5.0	6		+1			
Zahl der Tage mit Wetterleuchten	Tage	1951-1980	0.8	1.0	0		-1			
Z.d.T.m. Schneedecke ≥ 0 cm um 7:30 Uhr	Tage	1951-1980	0	0	0		0			
Z.d.T.m. Schneedecke ≥ 1 cm um 7:30 Uhr	Tage	1951-1980	0	0	0		0			
Z.d.T.m. Schneedecke ≥ 5 cm um 7:30 Uhr	Tage			0	0		0			
Z.d.T.m. Neuschnee ≥ 1 cm um 7:30 Uhr	Tage			0	0		0			
Summe der um 7:30 gem. Neuschneemenge	cm				0					
Max. Höhe der Schneedecke um 7:30 Uhr	cm			0.0			0		0 dbr	01.05.1970
Z.d.T.m. Glatteis durch gefrierenden Regen	Tage	1951-1980	0	0	0		0			
Z.d.T.m. Böen Windst. 6 Bft. (≥ 10,8 m/s)	Tage	1952-1980		13.0	22		+9			
Z.d.T.m. Böen Windst. 8 Bft. (≥ 17,2 m/s)	Tage	1952-1980		1.2	1		-0			
Maximale Windspitze	m/s			19.2	17.9	22.	-1.3		28.4*	16.5.1983
Heizgradsumme					83.9					
Kältesumme		1909-1969	0	0	0		0			

*) 1951-2010 +) 1881-2010

Manfred Wegener

Beiträge zur Berliner Wetterkarte

Klimatologische Mittelwerte von Berlin-Dahlem

38/11 ISSN 0177-3984
KBD VI/11 01.07.2011

Element	Maß-einheit	Beob-achtungs-periode	Vieljähr. Durch-schnitts-wert	Durch-schnitts-wert 1961-90	Juni 2011	Datum	Abweichung vom Durchschnitt 1961-90	in %	Bisherige Extreme 1909-2010	Datum
Luftdruck auf NN reduziert	hPa	1881-1970	1015.1	1015.3	1015.1		-0.2			
Höchster Luftdruck	hPa	1951-1980	1026.9	1026.4	1032.5	02.	+6.1		1035.0*	13.06.1957
Tiefster Luftdruck	hPa	1951-1980	1001.6	1001.3	999.5	08.	-1.8		993.9*	06.06.1986
Temperatur (stündl. Ablesung 01-24 MEZ)	°C	1955-1984	16.7	16.6	18.1		+1.5			
Temperatur (Klimamittel)	°C	1909-1969	16.7	16.7	18.1		+1.4			
Höchste Temperatur	°C	1909-1969	30.0	30.4	32.2	05.	+1.8		36.1	20.06.2000
Mittlere Maximum-Temperatur	°C	1909-1969	21.8	21.8	24.0		+2.2			
Tiefste Temperatur	°C	1909-1969	5.3	5.4	8.5	02.	+3.1		0.8	01.06.1977
Mittlere Minimum-Temperatur	°C	1909-1969	11.0	11.4	12.3		+0.9			
Tiefste Temperatur am Erdboden	°C	1951-1980	4.1	3.9	4.3	02.	+0.4		-0.6*	01.06.1977
Mittlere Min.-Temperatur am Erdboden	°C	1951-1980	10.0	*10.1*	8.7		*-1.4*			
Heiße Tage (Max.: ≥ 30,0°C)	Tage	1909-1969	1.4	1.2	2		+1			
Sommertage (Max.: ≥ 25,0°C)	Tage	1909-1969	7.7	7.8	12		+4			
(Max.: ≥ 20,0°C)	Tage			19.4	25		+6			
Frosttage (Min.: < 0,0°C)	Tage	1909-1969	0	0	0		0			
(Min.: ≤ -10,0°C)	Tage			0	0		0			
Eistage (Max.: < 0,0°C)	Tage	1909-1969	0	0	0		0			
Zahl d. Tage Min-Temp. am Erdb. < 0,0°C	Tage	1951-1980	0.1	0.0	0		-0			
Sonnenscheindauer	h	1951-1980	236.2	220.9	267.8		+46.9	121		
in % vom astronomisch möglichen	%		47.0	44.0	53.3		+9.3			
Zahl der sonnenscheinlosen Tage	Tage	1951-1980	1.3	1.3	0		-1			
Bewölkung (in Achtel)	/8	1909-1969	4.9	4.8	4.4		-0.4			
Heitere Tage (< 1,6 Achtel Bewölkung)	Tage	1909-1969	2.8	2.8	5		+2			
Trübe Tage (> 6,4 Achtel Bewölkung)	Tage	1909-1969	8.2	8.0	3		-5			
Dampfdruck	hPa	1909-1969	12.8	13.1	13.1		0.0			
Relative Luftfeuchtigkeit	%	1909-1969	68	69	66		-3			
Niederschlagshöhe	mm	1909-1969	64.2	70.7	41.5		-29.2	59		
Maximale Tagesmenge	mm	1909-1969	19.6	22.1	15.1	22.	-7.0		57.9	28.06.1964
Zahl der Tage mit Sturzregen	Tage			0.2	-		-			
Zahl der Tage mit ≥ 10,0 mm Niederschlag	Tage	1909-1969	1.7	2.0	1		-1			
Zahl der Tage mit ≥ 5,0 mm Niederschlag	Tage			4.2	2		-2			
Zahl der Tage mit ≥ 2,5 mm Niederschlag	Tage	1909-1969	6.4	7.0	5		-2			
Zahl der Tage mit ≥ 1,0 mm Niederschlag	Tage	1909-1969	9.5	10.2	10		-0			
Zahl der Tage mit ≥ 0,1 mm Niederschlag	Tage	1909-1969	13.4	14.9	15		+0			
Z.d.T.m. gefall. flüss. Niederschl. ≥ 0,1 mm	Tage			14.6	15		+0			
Z.d.T.m. flüss. u. fest. Niederschl. ≥ 0,1 mm	Tage			0.3	0		-0			
Z.d.T.m. gefall. fest. Niederschl. ≥ 0,1 mm	Tage			0	0		0			
Z.d.T.m. abgesetzt. Niederschl. ≥ 0,1 mm	Tage			0.1	-		-			
Z.d.T.m. Hagel ≥ 0,0 mm	Tage			0.2	0		-0			
Z.d.T.m. Graupel, Griesel o. Eisk. ≥ 0,0 mm	Tage			0.0	0		-0			
Zahl der Tage mit Reif oder Rauhreif	Tage	1964-1980	0	0	-		-			
Zahl der Tage mit Tau	Tage	1964-1980	25.4	24.3	-		-			
Zahl der Tage mit Nebel	Tage	1951-1980	0.3	0.3	0		-0			
Zahl der Tage mit Gewitter	Tage	1951-1980	7.4	6.4	9		+3			
Zahl der Tage mit Wetterleuchten	Tage	1951-1980	1.0	0.9	3		+2			
Z.d.T.m. Schneedecke ≥ 0 cm um 7:30 Uhr	Tage	1951-1980	0	0	0		0			
Z.d.T.m. Schneedecke ≥ 1 cm um 7:30 Uhr	Tage	1951-1980	0	0	0		0			
Z.d.T.m. Schneedecke ≥ 5 cm um 7:30 Uhr	Tage			0	0		0			
Z.d.T.m. Neuschnee ≥ 1 cm um 7:30 Uhr	Tage			0	0		0			
Summe der um 7:30 gem. Neuschneemenge	cm									
Max. Höhe der Schneedecke um 7:30 Uhr	cm									
Z.d.T.m. Glatteis durch gefrierenden Regen	Tage	1951-1980	0	0	0		0			
Z.d.T.m. Böen Windst. 6 Bft. (≥ 10,8 m/s)	Tage	1952-1980		12.4	19		+7			
Z.d.T.m. Böen Windst. 8 Bft. (≥ 17,2 m/s)	Tage	1952-1980		0.9	3		+2			
Maximale Windspitze	m/s			18.7	20.3	22.	+1.6		32.2*	23.6.2003
Heizgradsumme										
Kältesumme		1909-1969	0	0	0		0			

*) 1951-2010 +) 1881-2010

Manfred Wegener

Beiträge zur Berliner Wetterkarte

Klimatologische Mittelwerte von Berlin-Dahlem

43/11 ISSN 0177-3984
KBD VII/11 02.08.2011

Element	Maß-einheit	Beob-achtungs-periode	Vieljähr. Durch-schnitts-wert	Durch-schnitts-wert 1961-90	**JULI 2011**	Datum	Abweichung vom Durchschnitt 1961-90	in %	Bisherige Extreme 1909-2010	Datum
Luftdruck auf NN reduziert	hPa	1881-1970	1014.2	1015.5	1010.4		-5.1			
Höchster Luftdruck	hPa	1951-1980	1024.9	1025.4	1019.7	11./12.	-5.7		1032.4*	16.07.2006
Tiefster Luftdruck	hPa	1951-1980	1001.7	1001.9	999.4	17.	-2.5		989.6*	18.07.1954
Temperatur (stündl. Ablesung 01-24 MEZ)	°C	1955-1984	18.0	17.9	17.3		-0.6			
Temperatur (Klimamittel)	°C	1909-1969	18.3	17.9	17.3		-0.6			
Höchste Temperatur	°C	1909-1969	31.5	30.9	28.9	09.	-2.0		37.8	11.07.1959
Mittlere Maximum-Temperatur	°C	1909-1969	23.5	23.1	21.4		-1.7			
Tiefste Temperatur	°C	1909-1969	8.3	8.0	8.7	01.	+0.7		5.4	09.07.1948
Mittlere Minimum-Temperatur	°C	1909-1969	13.2	12.9	13.2		+0.3			
Tiefste Temperatur am Erdboden	°C	1951-1980	6.6	6.7	5.5	01.	-1.2		4.6*	07.07.1964
Mittlere Min.-Temperatur am Erdboden	°C	1951-1980	11.6	*11.6*	11.3		*-0.3*			
Heiße Tage (Max.: ≥ 30,0°C)	Tage	1909-1969	2.4	2.5	0		-3			
Sommertage (Max.: ≥ 25,0°C)	Tage	1909-1969	10.7	10.3	8		-2			
(Max.: ≥ 20,0°C)	Tage			22.6	19		-4			
Frosttage (Min.: < 0,0°C)	Tage	1909-1969	0	0	0		0			
(Min.: ≤ -10,0°C)	Tage			0	0		0			
Eistage (Max.: < 0,0°C)	Tage	1909-1969	0	0	0		0			
Zahl d. Tage Min-Temp. am Erdb. < 0,0°C	Tage	1951-1980	0	0	0		0			
Sonnenscheindauer	h	1951-1980	220.6	218.0	166.8		-51.2	77		
in % vom astronomisch möglichen	%		43.7	43.2	33.1		-10.1			
Zahl der sonnenscheinlosen Tage	Tage	1951-1980	1.1	0.8	8		+7			
Bewölkung (in Achtel)	/8	1909-1969	5.0	4.8	5.8		+1.0			
Heitere Tage (< 1,6 Achtel Bewölkung)	Tage	1909-1969	2.1	3.1	0		-3			
Trübe Tage (> 6,4 Achtel Bewölkung)	Tage	1909-1969	9.1	8.4	12		+4			
Dampfdruck	hPa	1909-1969	15.0	14.4	14.1		-0.3			
Relative Luftfeuchtigkeit	%	1909-1969	72	70	74		+4			
Niederschlagshöhe	mm	1909-1969	72.1	53.1	202.0		+148.9	380		
Maximale Tagesmenge	mm	1909-1969	21.3	15.6	52.9	29.	+37.3		65.3	26.07.1930
Zahl der Tage mit Sturzregen	Tage			0.2	-		-			
Zahl der Tage mit ≥ 10,0 mm Niederschlag	Tage	1909-1969	1.9	1.2	8		+7			
Zahl der Tage mit ≥ 5,0 mm Niederschlag	Tage			3.5	11		+7			
Zahl der Tage mit ≥ 2,5 mm Niederschlag	Tage	1909-1969	7.3	5.9	12		+6			
Zahl der Tage mit ≥ 1,0 mm Niederschlag	Tage	1909-1969	10.4	9.0	14		+5			
Zahl der Tage mit ≥ 0,1 mm Niederschlag	Tage	1909-1969	14.6	13.9	17		+3			
Z.d.T.m. gefall. flüss. Niederschl. ≥ 0,1 mm	Tage			13.6	17		+3			
Z.d.T.m. flüss. u. fest. Niederschl. ≥ 0,1 mm	Tage			0.2	0		-0			
Z.d.T.m. gefall. fest. Niederschl. ≥ 0,1 mm	Tage			0	0		0			
Z.d.T.m. abgesetzt. Niederschl. ≥ 0,1 mm	Tage			0.1	-		-			
Z.d.T.m. Hagel ≥ 0,0 mm	Tage			0.2	0		-0			
Z.d.T.m. Graupel, Griesel o. Eisk. ≥ 0,0 mm	Tage			0.0	0		-0			
Zahl der Tage mit Reif oder Rauhreif	Tage	1964-1980	0	0	-		-			
Zahl der Tage mit Tau	Tage	1964-1980	27.7	26.1	-		-			
Zahl der Tage mit Nebel	Tage	1951-1980	0.6	0.3	2		+2			
Zahl der Tage mit Gewitter	Tage	1951-1980	5.9	5.3	2		-3			
Zahl der Tage mit Wetterleuchten	Tage	1951-1980	0.8	0.6	3		+2			
Z.d.T.m. Schneedecke ≥ 0 cm um 7:30 Uhr	Tage	1951-1980	0	0	0		0			
Z.d.T.m. Schneedecke ≥ 1 cm um 7:30 Uhr	Tage	1951-1980	0	0	0		0			
Z.d.T.m. Schneedecke ≥ 5 cm um 7:30 Uhr	Tage			0	0		0			
Z.d.T.m. Neuschnee ≥ 1 cm um 7:30 Uhr	Tage		0	0	0		0			
Summe der um 7:30 gem. Neuschneemenge	cm				.					
Max. Höhe der Schneedecke um 7:30 Uhr	cm									
Z.d.T.m. Glatteis durch gefrierenden Regen	Tage	1951-1980	0	0	0		0			
Z.d.T.m. Böen Windst. 6 Bft. (≥ 10,8 m/s)	Tage	1952-1980		12.0	19		+7			
Z.d.T.m. Böen Windst. 8 Bft. (≥ 17,2 m/s)	Tage	1952-1980		0.7	3		+2			
Maximale Windspitze	m/s			18.1	19.8	22.	+1.7		33.2*	10.7.2002
Heizgradsumme										
Kältesumme		1909-1969		0	0		0			

*) 1951-2010 +) 1881-2010

Manfred Wegener

Beiträge zur Berliner Wetterkarte

Klimatologische Mittelwerte von Berlin-Dahlem

48/11 ISSN 0177-3984
KBD VIII/11 01.09.2011

Element	Maß-einheit	Beob-achtungs-periode	Vieljähr. Durch-schnitts-wert	Durch-schnitts-wert 1961-90	**AUGUST 2011**	Datum	Abweichung vom Durchschnitt 1961-90	in %	Bisherige Extreme 1909-2011	Datum
Luftdruck auf NN reduziert	hPa	1881-1970	1014.4	1015.5	1013.4		-2.1			
Höchster Luftdruck	hPa	1951-1980	1025.8	1026.4	1022.0	20.	-4.4		1031.5+	04.08.1981
Tiefster Luftdruck	hPa	1951-1980	1000.9	1001.6	998.3	24.	-3.3		984.2+	25.08.1956
Temperatur (stündl. Ablesung 01-24 MEZ)	°C	1955-1984	17.3	17.3	18.1		+0.8			
Temperatur (Klimamittel)	°C	1909-1969	17.3	17.2	18.1		+0.9			
Höchste Temperatur	°C	1909-1969	30.4	30.9	31.0	26.	+0.1		37.7	01.08.1994
Mittlere Maximum-Temperatur	°C	1909-1969	22.7	22.8	23.1		+0.3			
Tiefste Temperatur	°C	1909-1969	7.6	7.0	7.7	31.	+0.7		4.7	21.08.1964
Mittlere Minimum-Temperatur	°C	1909-1969	12.5	12.4	13.4		+1.0			
Tiefste Temperatur am Erdboden	°C	1951-1980	5.8	*5.8*	4.8	31.	-1.0		3.2*	21.08.1964
Mittlere Min.-Temperatur am Erdboden	°C	1951-1980	11.1	*11.2*	10.8		*-0.4*			
Heiße Tage (Max.: ≥ 30,0°C)	Tage	1909-1969	1.6	1.6	1		-1			
Sommertage (Max.: ≥ 25,0°C)	Tage	1909-1969	8.6	9.1	8		-1			
(Max.: ≥ 20,0°C)	Tage			23.0	26		+3			
Frosttage (Min.: < 0,0°C)	Tage	1909-1969	0	0	0		0			
(Min.: ≤ -10,0°C)	Tage			0	0		0			
Eistage (Max.: < 0,0°C)	Tage	1909-1969	0	0	0		0			
Zahl d. Tage Min-Temp. am Erdb. < 0,0°C	Tage	1951-1980	0	0	0		0			
Sonnenscheindauer	h	1951-1980	209.1	210.2	184.3		-25.9	88		
in % vom astronomisch möglichen	%		45.9	46.2	40.5		-5.7			
Zahl der sonnenscheinlosen Tage	Tage	1951-1980	1.1	1.2	1		-0			
Bewölkung (in Achtel)	/8	1909-1969	4.8	4.5	5.1		+0.6			
Heitere Tage (< 1,6 Achtel Bewölkung)	Tage	1909-1969	2.9	4.3	2		-2			
Trübe Tage (> 6,4 Achtel Bewölkung)	Tage	1909-1969	8.2	7.0	6		-1			
Dampfdruck	hPa	1909-1969	14.7	14.4	15.3		+0.9			
Relative Luftfeuchtigkeit	%	1909-1969	75	73	75		+2			
Niederschlagshöhe	mm	1909-1969	66.0	65.3	90.1		+24.8	138		
Maximale Tagesmenge	mm	1909-1969	21.0	24.6	20.9	14.	-3.7		124.7	14.08.1948
Zahl der Tage mit Sturzregen	Tage			0.4	-		-			
Zahl der Tage mit ≥ 10,0 mm Niederschlag	Tage	1909-1969	1.9	1.9	3		+1			
Zahl der Tage mit ≥ 5,0 mm Niederschlag	Tage			3.6	7		+3			
Zahl der Tage mit ≥ 2,5 mm Niederschlag	Tage	1909-1969	6.4	6.2	7		+1			
Zahl der Tage mit ≥ 1,0 mm Niederschlag	Tage	1909-1969	9.8	9.0	10		+1			
Zahl der Tage mit ≥ 0,1 mm Niederschlag	Tage	1909-1969	14.3	13.4	17		+4			
Z.d.T.m. gefall. flüss. Niederschl. ≥ 0,1 mm	Tage			12.6	16		+3			
Z.d.T.m. flüss. u. fest. Niederschl. ≥ 0,1 mm	Tage			0.4	1		+1			
Z.d.T.m. gefall. fest. Niederschl. ≥ 0,1 mm	Tage			0	0		0			
Z.d.T.m. abgesetzt. Niederschl. ≥ 0,1 mm	Tage			0.4	-		-			
Z.d.T.m. Hagel ≥ 0,0 mm	Tage			0.4	0		-0			
Z.d.T.m. Graupel, Griesel o. Eisk. ≥ 0,0 mm	Tage			0.0	1		+1			
Zahl der Tage mit Reif oder Rauhreif	Tage	1964-1980	0	0	-		-			
Zahl der Tage mit Tau	Tage	1964-1980	27.2	27.0	-		-			
Zahl der Tage mit Nebel	Tage	1951-1980	1.3	1.0	1		-0			
Zahl der Tage mit Gewitter	Tage	1951-1980	5.3	4.8	7		+2			
Zahl der Tage mit Wetterleuchten	Tage	1951-1980	1.6	1.3	9		+8			
Z.d.T.m. Schneedecke ≥ 0 cm um 7:30 Uhr	Tage	1951-1980	0	0	0		0			
Z.d.T.m. Schneedecke ≥ 1 cm um 7:30 Uhr	Tage	1951-1980	0	0	0		0			
Z.d.T.m. Schneedecke ≥ 5 cm um 7:30 Uhr	Tage			0	0		0			
Z.d.T.m. Neuschnee ≥ 1 cm um 7:30 Uhr	Tage			0	0		0			
Summe der um 7:30 gem. Neuschneemenge	cm			.	0					
Max. Höhe der Schneedecke um 7:30 Uhr	cm			.						
Z.d.T.m. Glatteis durch gefrierenden Regen	Tage	1951-1980	0	0	0		0			
Z.d.T.m. Böen Windst. 6 Bft. (≥ 10,8 m/s)	Tage	1952-1980		9.5	14		+4			
Z.d.T.m. Böen Windst. 8 Bft. (≥ 17,2 m/s)	Tage	1952-1980		0.7	4		+3			
Maximale Windspitze	m/s			17.7	22.3	08.	+4.6		26.3*	04.08.1974
Heizgradsumme										
Kältesumme		1909-1969	0	0	0		0			

*) 1951-2011 +) 1881-2011

Manfred Wegener

Beiträge zur Berliner Wetterkarte

Klimatologische Mittelwerte von Berlin-Dahlem

54/11
KBD IX/11

ISSN 0177-3984
05.10.2011

Element	Maßeinheit	Beobachtungsperiode	Vieljähr. Durchschnittswert	Durchschnittswert 1961-90	**September 2011**	Datum	Abweichung vom Durchschnitt 1961-90	in %	Bisherige Extreme 1909-2010	Datum
Luftdruck auf NN reduziert	hPa	1881-1970	1016.5	1016.7	1015.9		-0.8			
Höchster Luftdruck	hPa	1951-1980	1030.1	1030.0	1032.7	28.	+2.7		1038.5+	07.09.1953
Tiefster Luftdruck	hPa	1951-1980	1000.8	1000.4	1003.2	18.	+2.8		987.2+	21.09.1990
Temperatur (stündl. Ablesung 01-24 MEZ)	°C	1955-1984	13.7	13.6	15.3		+1.7			
Temperatur (Klimamittel)	°C	1909-1969	13.9	13.5	15.3		+1.8			
Höchste Temperatur	°C	1909-1969	27.0	26.1	28.4	04.	+2.3		34.2	03.09.1911<br>12.9.1919
Mittlere Maximum-Temperatur	°C	1909-1969	19.3	18.7	20.7		+2.0			
Tiefste Temperatur	°C	1909-1969	3.4	4.0	5.1	24.	+1.1		-0.5	21.09.1915
Mittlere Minimum-Temperatur	°C	1909-1969	9.4	9.4	10.2		+0.8			
Tiefste Temperatur am Erdboden	°C	1951-1980	1.8	2.6	2.8	24.	+0.2		-1.9*	21.09.1997
Mittlere Min.-Temperatur am Erdboden	°C	1951-1980	7.9	*8.3*	7.5		*-0.8*			
Heiße Tage (Max.: ≥ 30,0°C)	Tage	1909-1969	0.4	0.1	0		-0			
Sommertage (Max.: ≥ 25,0°C)	Tage	1909-1969	2.9	2.1	4		+2			
(Max.: ≥ 20,0°C)	Tage			10.8	16		+5			
Frosttage (Min.: < 0,0°C)	Tage	1909-1969	0	0	0		0			
(Min.: ≤ -10,0°C)	Tage			0	0		0			
Eistage (Max.: < 0,0°C)	Tage	1909-1969	0	0	0		0			
Zahl d. Tage Min-Temp. am Erdb. < 0,0°C	Tage	1951-1980	0.3	0.1	0		-0			
Sonnenscheindauer	h	1951-1980	171.5	156.3	202.8		+46.5	130		
in % vom astronomisch möglichen	%		45.0	41.0	53.2		+12.2			
Zahl der sonnenscheinlosen Tage	Tage	1951-1980	2.2	2.4	2		-0			
Bewölkung (in Achtel)	/8	1909-1969	4.6	4.7	4.5		-0.2			
Heitere Tage (< 1,6 Achtel Bewölkung)	Tage	1909-1969	4.3	3.7	3		-1			
Trübe Tage (> 6,4 Achtel Bewölkung)	Tage	1909-1969	7.4	8.4	6		-2			
Dampfdruck	hPa	1909-1969	12.5	12.6	13.1		+0.5			
Relative Luftfeuchtigkeit	%	1909-1969	79	80	77		-3			
Niederschlagshöhe	mm	1909-1969	45.7	45.5	58.3		+12.8	128		
Maximale Tagesmenge	mm	1909-1969	14.2	15.4	16.6	11.	+1.2		40.0	05.09.1931
Zahl der Tage mit Sturzregen	Tage			0.1	-		-			
Zahl der Tage mit ≥ 10,0 mm Niederschlag	Tage	1909-1969	1.0	1.0	2		+1			
Zahl der Tage mit ≥ 5,0 mm Niederschlag	Tage			2.7	3		+0			
Zahl der Tage mit ≥ 2,5 mm Niederschlag	Tage	1909-1969	5.5	5.2	9		+4			
Zahl der Tage mit ≥ 1,0 mm Niederschlag	Tage	1909-1969	8.4	8.6	9		+0			
Zahl der Tage mit ≥ 0,1 mm Niederschlag	Tage	1909-1969	12.6	14.4	10		-4			
Z.d.T.m. gefall. flüss. Niederschl. ≥ 0,1 mm	Tage			14.0	10		-4			
Z.d.T.m. flüss. u. fest. Niederschl. ≥ 0,1 mm	Tage			0.2	0		-0			
Z.d.T.m. gefall. fest. Niederschl. ≥ 0,1 mm	Tage			0.0	0		-0			
Z.d.T.m. abgesetzt. Niederschl. ≥ 0,1 mm	Tage			0.2	-		-			
Z.d.T.m. Hagel ≥ 0,0 mm	Tage			0.1	0		-0			
Z.d.T.m. Graupel, Griesel o. Eisk. ≥ 0,0 mm	Tage			0.1	0		-0			
Zahl der Tage mit Reif oder Rauhreif	Tage	1964-1980	0.2	0.1	-		-			
Zahl der Tage mit Tau	Tage	1964-1980	27.4	27.2	-		-			
Zahl der Tage mit Nebel	Tage	1951-1980	2.4	1.8	0		-2			
Zahl der Tage mit Gewitter	Tage	1951-1980	2.4	2.4	2		-0			
Zahl der Tage mit Wetterleuchten	Tage	1951-1980	0.6	0.5	1		+0			
Z.d.T.m. Schneedecke ≥ 0 cm um 7:30 Uhr	Tage	1951-1980	0	0	0		0			
Z.d.T.m. Schneedecke ≥ 1 cm um 7:30 Uhr	Tage	1951-1980	0	0	0		0			
Z.d.T.m. Schneedecke ≥ 5 cm um 7:30 Uhr	Tage			0	0		0			
Z.d.T.m. Neuschnee ≥ 1 cm um 7:30 Uhr	Tage			0	0		0			
Summe der um 7:30 gem. Neuschneemenge	cm			.	0					
Max. Höhe der Schneedecke um 7:30 Uhr	cm			.						
Z.d.T.m. Glatteis durch gefrierenden Regen	Tage	1951-1980	0	0	0		0			
Z.d.T.m. Böen Windst. 6 Bft. (≥ 10,8 m/s)	Tage	1952-1980		9.8	10		+0			
Z.d.T.m. Böen Windst. 8 Bft. (≥ 17,2 m/s)	Tage	1952-1980		1.2	1		-0			
Maximale Windspitze	m/s			18.5	17.9	07.	-0.6		24.7*	9.9.1997
Heizgradsumme					23.5					
Kältesumme		1909-1969	0	0	0		0			

*) 1951-20010 +) 1881-2010

Manfred Wegener

Beiträge zur Berliner Wetterkarte

Klimatologische Mittelwerte von Berlin-Dahlem

58/11 ISSN 0177-3984
KBD XI/11 02.11.2011

Element	Maß-einheit	Beob-achtungs-periode	Vieljähr. Durch-schnitts-wert	Durch-schnitts-wert 1961-90	**Oktober 2011**	Datum	Abweichung vom Durchschnitt 1961-90	in %	Bisherige Extreme 1909-2010	Datum
Luftdruck auf NN reduziert	hPa	1881-1970	1015.9	1017.3	1019.8		+2.5			
Höchster Luftdruck	hPa	1951-1980	1032.5	1033.6	1036.7	14.	+3.1		1042.1+	18.10.1993
Tiefster Luftdruck	hPa	1951-1980	998.1	996.0	1001.5	06.	+5.5		979.3+	22.10.1974
Temperatur (stündl. Ablesung 01-24 MEZ)	°C	1955-1984	9.1	9.3	9.7		+0.4			
Temperatur (Klimamittel)	°C	1909-1969	9.0	9.3	9.7		+0.4			
Höchste Temperatur	°C	1909-1969	20.5	21.3	26.5	01.	+5.2		27.5	04.10.1985
Mittlere Maximum-Temperatur	°C	1909-1969	13.1	13.3	14.9		+1.6			
Tiefste Temperatur	°C	1909-1969	-1.1	-0.4	-1.2	15.	-0.8		-9.6	29.10.1915
Mittlere Minimum-Temperatur	°C	1909-1969	5.4	5.9	5.3		-0.6			
Tiefste Temperatur am Erdboden	°C	1951-1980	-2.0	*-2.1*	-3.3	22.	*-1.2*		-8.8*	28.10.1997
Mittlere Min.-Temperatur am Erdboden	°C	1951-1980	4.5	*4.6*	2.8		*-1.8*			
Heiße Tage (Max.: ≥ 30,0°C)	Tage	1909-1969	0	0	0		0			
Sommertage (Max.: ≥ 25,0°C)	Tage	1909-1969	0.1	0.1	2		+2			
(Max.: ≥ 20,0°C)	Tage			2.0	4		+2			
Frosttage (Min.: < 0,0°C)	Tage	1909-1969	2.3	1.3	4		+3			
(Min.: ≤ -10,0°C)	Tage			0	0		0			
Eistage (Max.: < 0,0°C)	Tage	1909-1969	0.0	0	0		0			
Zahl d. Tage Min-Temp. am Erdb. < 0,0°C	Tage	1951-1980	3.7	3.6	10		+6			
Sonnenscheindauer	h	1951-1980	112.2	110.8	167.1		+56.3	151		
in % vom astronomisch möglichen	%		33.9	33.5	50.5		+17.0			
Zahl der sonnenscheinlosen Tage	Tage	1951-1980	6.6	6.4	3		-3			
Bewölkung (in Achtel)	/8	1909-1969	5.2	5.2	4.2		-1.0			
Heitere Tage (< 1,6 Achtel Bewölkung)	Tage	1909-1969	3.3	3.0	6		+3			
Trübe Tage (> 6,4 Achtel Bewölkung)	Tage	1909-1969	11.6	11.3	6		-5			
Dampfdruck	hPa	1909-1969	9.7	10.0	9.9		-0.1			
Relative Luftfeuchtigkeit	%	1909-1969	83	83	81		-2			
Niederschlagshöhe	mm	1909-1969	44.2	35.8	37.5		+1.7	105		
Maximale Tagesmenge	mm	1909-1969	11.8	9.4	13.8	11.	+4.4		36.3	19.10.1941
Zahl der Tage mit Sturzregen	Tage			0	-		-			
Zahl der Tage mit ≥ 10,0 mm Niederschlag	Tage	1909-1969	0.8	0.5	1		+0			
Zahl der Tage mit ≥ 5,0 mm Niederschlag	Tage			2.4	3		+1			
Zahl der Tage mit ≥ 2,5 mm Niederschlag	Tage	1909-1969	5.5	4.8	5		+0			
Zahl der Tage mit ≥ 1,0 mm Niederschlag	Tage	1909-1969	8.9	7.9	7		-1			
Zahl der Tage mit ≥ 0,1 mm Niederschlag	Tage	1909-1969	13.6	14.3	10		-4			
Z.d.T.m. gefall. flüss. Niederschl. ≥ 0,1 mm	Tage			13.1	10		-3			
Z.d.T.m. flüss. u. fest. Niederschl. ≥ 0,1 mm	Tage			0.6	0		-1			
Z.d.T.m. gefall. fest. Niederschl. ≥ 0,1 mm	Tage			0	0		0			
Z.d.T.m. abgesetzt. Niederschl. ≥ 0,1 mm	Tage			0.6	-		-			
Z.d.T.m. Hagel ≥ 0,0 mm	Tage			0.2	0		-0			
Z.d.T.m. Graupel, Griesel o. Eisk. ≥ 0,0 mm	Tage			0.4	0		-0			
Zahl der Tage mit Reif oder Rauhreif	Tage	1964-1980	3.8	3.3	-		-			
Zahl der Tage mit Tau	Tage	1964-1980	23.8	24.5	-		-			
Zahl der Tage mit Nebel	Tage	1951-1980	6.6	5.9	2		-4			
Zahl der Tage mit Gewitter	Tage	1951-1980	0.3	0.4	0		-0			
Zahl der Tage mit Wetterleuchten	Tage	1951-1980	0.0	0.0	1		+1			
Z.d.T.m. Schneedecke ≥ 0 cm um 7:30 Uhr	Tage	1951-1980	0	0	0		0			
Z.d.T.m. Schneedecke ≥ 1 cm um 7:30 Uhr	Tage	1951-1980	0	0	0		0			
Z.d.T.m. Schneedecke ≥ 5 cm um 7:30 Uhr	Tage			0	0		0			
Z.d.T.m. Neuschnee ≥ 1 cm um 7:30 Uhr	Tage			0	0		0			
Summe der um 7:30 gem. Neuschneemenge	cm									
Max. Höhe der Schneedecke um 7:30 Uhr	cm								0 Fl	14.10.2002
Z.d.T.m. Glatteis durch gefrierenden Regen	Tage	1951-1980	0	0.0	0		-0			
Z.d.T.m. Böen Windst. 6 Bft. (≥ 10,8 m/s)	Tage	1952-1980		10.5	14		+3			
Z.d.T.m. Böen Windst. 8 Bft. (≥ 17,2 m/s)	Tage	1952-1980		1.3	3		+2			
Maximale Windspitze	m/s			19.8	20.8	06.	+1.0		29.7*	27.10.2002
Heizgradsumme					327.8					
Kältesumme		1909-1969	0.4	0	0		0			

*) 1951-2010 +) 1881-2010

Manfred Wegener

Beiträge zur Berliner Wetterkarte

Klimatologische Mittelwerte von Berlin-Dahlem

66/11 ISSN 0177-3984
KBD XI/11 01.12.2011

Element	Maß-einheit	Beob-achtungs-periode	Vieljähr. Durch-schnitts-wert	Durch-schnitts-wert 1961-90	**November 2011**	Datum	Abweichung vom Durchschnitt 1961-90	in %	Bisherige Extreme 1909-2010	Datum
Luftdruck auf NN reduziert	hPa	1881-1970	1015.4	1014.8	1023.1		+8.3			
Höchster Luftdruck	hPa	1951-1980	1032.9	1034.0	1037.2	12.	+3.2		1043.3+	21.11.1998
Tiefster Luftdruck	hPa	1951-1980	991.0	988.2	1008.7	04.	+20.5		966.8+	27.11.1983
Temperatur (stündl. Ablesung 01-24 MEZ)	°C	1955-1984	4.5	4.5	4.4		-0.1			
Temperatur (Klimamittel)	°C	1909-1969	4.2	4.6	4.4		-0.2			
Höchste Temperatur	°C	1909-1969	13.5	14.1	15.2	06.	+1.1		19.5	01.11.1968
Mittlere Maximum-Temperatur	°C	1909-1969	6.7	7.0	8.5		+1.5			
Tiefste Temperatur	°C	1909-1969	-4.7	-4.8	-3.2	14.	+1.6		-16.1	23.11.1965
Mittlere Minimum-Temperatur	°C	1909-1969	1.7	2.1	1.1		-1.0			
Tiefste Temperatur am Erdboden	°C	1951-1980	-6.4	*-6.7*	-5.2	12./13./14.	*+1.5*		-24.2*	23.11.1965
Mittlere Min.-Temperatur am Erdboden	°C	1951-1980	1.0	*1.1*	-1.3		*-2.4*			
Heiße Tage (Max.: ≥ 30,0°C)	Tage	1909-1969	0	0	0		0			
Sommertage (Max.: ≥ 25,0°C)	Tage	1909-1969	0	0	0		0			
(Max.: ≥ 20,0°C)	Tage			0	0		0			
Frosttage (Min.: < 0,0°C)	Tage	1909-1969	9.5	8.4	15		+7			
(Min.: ≤ -10,0°C)	Tage			0.1	0		-0			
Eistage (Max.: < 0,0°C)	Tage	1909-1969	1.3	1.3	0		-1			
Zahl d. Tage Min-Temp. am Erdb. < 0,0°C	Tage	1951-1980	11.2	11.5	19		+7			
Sonnenscheindauer	h	1951-1980	47.7	52.4	138.9		+86.5	265		
in % vom astronomisch möglichen	%		18.0	19.8	52.5		+32.7			
Zahl der sonnenscheinlosen Tage	Tage	1951-1980	13.2	12.0	7		-5			
Bewölkung (in Achtel)	/8	1909-1969	6.2	6.0	4.0		-2.0			
Heitere Tage (< 1,6 Achtel Bewölkung)	Tage	1909-1969	1.3	1.3	6		+5			
Trübe Tage (> 6,4 Achtel Bewölkung)	Tage	1909-1969	16.3	16.1	6		-10			
Dampfdruck	hPa	1909-1969	7.3	7.4	7.2		-0.2			
Relative Luftfeuchtigkeit	%	1909-1969	87	85	86		+1			
Niederschlagshöhe	mm	1909-1969	47.7	49.5	0.4		-49.1	1		
Maximale Tagesmenge	mm	1909-1969	12.7	14.0	0.4	25.	-13.6		47.5	27.11.1926
Zahl der Tage mit Sturzregen	Tage			0	-		-			
Zahl der Tage mit ≥ 10,0 mm Niederschlag	Tage	1909-1969	1.0	0.9	0		-1			
Zahl der Tage mit ≥ 5,0 mm Niederschlag	Tage			3.1	0		-3			
Zahl der Tage mit ≥ 2,5 mm Niederschlag	Tage	1909-1969	5.7	6.3	0		-6			
Zahl der Tage mit ≥ 1,0 mm Niederschlag	Tage	1909-1969	9.8	10.1	0		-10			
Zahl der Tage mit ≥ 0,1 mm Niederschlag	Tage	1909-1969	16.1	17.0	1		-16			
Z.d.T.m. gefall. flüss. Niederschl. ≥ 0,1 mm	Tage			12.2	1		-11			
Z.d.T.m. flüss. u. fest. Niederschl. ≥ 0,1 mm	Tage			3.7	0		-4			
Z.d.T.m. gefall. fest. Niederschl. ≥ 0,1 mm	Tage			1.0	0		-1			
Z.d.T.m. abgesetzt. Niederschl. ≥ 0,1 mm	Tage			0.1	-		-			
Z.d.T.m. Hagel ≥ 0,0 mm	Tage			0.2	0		-0			
Z.d.T.m. Graupel, Griesel o. Eisk. ≥ 0,0 mm	Tage			2.5	0		-3			
Zahl der Tage mit Reif oder Rauhreif	Tage	1964-1980	9.5	9.7	-		-			
Zahl der Tage mit Tau	Tage	1964-1980	12.1	13.6	-		-			
Zahl der Tage mit Nebel	Tage	1951-1980	6.1	5.4	8		+3			
Zahl der Tage mit Gewitter	Tage	1951-1980	0.4	0.5	0		-1			
Zahl der Tage mit Wetterleuchten	Tage	1951-1980	0.0	0.1	0		-0			
Z.d.T.m. Schneedecke ≥ 0 cm um 7:30 Uhr	Tage	1951-1980	1.8	2.6	0		-3			
Z.d.T.m. Schneedecke ≥ 1 cm um 7:30 Uhr	Tage	1951-1980	1.6	2.2	0		-2			
Z.d.T.m. Schneedecke ≥ 5 cm um 7:30 Uhr	Tage			1.0	0		-1			
Z.d.T.m. Neuschnee ≥ 1 cm um 7:30 Uhr	Tage			1.2	0		-1			
Summe der um 7:30 gem. Neuschneemenge	cm									
Max. Höhe der Schneedecke um 7:30 Uhr	cm			3.4			-3		27	17./18.11.1919
Z.d.T.m. Glatteis durch gefrierenden Regen	Tage	1951-1980	0.5	0.5	0		-1			
Z.d.T.m. Böen Windst. 6 Bft. (≥ 10,8 m/s)	Tage	1952-1980		13.3	8		-5			
Z.d.T.m. Böen Windst. 8 Bft. (≥ 17,2 m/s)	Tage	1952-1980		2.7	1		-2			
Maximale Windspitze	m/s			22.1	22.6	27.	+0.5		36.5*	13.11.1972
Heizgradsumme					473.6					
Kältesumme		1909-1969	7.3	6.5	0.5		-6.0			

*) 1951-2010 +) 1881-2010

Manfred Wegener

Beiträge zur Berliner Wetterkarte

Klimatologische Mittelwerte von Berlin-Dahlem

01/12 ISSN 0177-3984
KBD XII/11 03.01.2012

Element	Maßeinheit	Beobachtungsperiode	Vieljähr. Durchschnittswert	Durchschnittswert 1961-90	**Dezember 2011**	Datum	Abweichung vom Durchschnitt 1961-90	in %	Bisherige Extreme 1909-2010	Datum
Luftdruck auf NN reduziert	hPa	1881-1970	1015.2	1015.1	1009.0		-6.1			
Höchster Luftdruck	hPa	1951-1980	1034.8	1036.6	1033.7	27.	-2.9		1049.4+	23.12.1963
Tiefster Luftdruck	hPa	1951-1980	987.7	988.3	966.0	16.	-22.3		970.2+	16.12.1962
Temperatur (stündl. Ablesung 01-24 MEZ)	°C	1955-1984	0.9	1.1	4.4		+3.3			
Temperatur (Klimamittel)	°C	1909-1969	0.9	1.2	4.4		+3.2			
Höchste Temperatur	°C	1909-1969	9.9	10.5	12.2	02.	+1.7		15.7	24.12.1977
Mittlere Maximum-Temperatur	°C	1909-1969	3.0	3.2	6.5		+3.3			
Tiefste Temperatur	°C	1909-1969	-9.6	-9.2	-1.6	01./12.	+7.6		-20.2	21.12.1969
Mittlere Minimum-Temperatur	°C	1909-1969	-1.5	-1.1	2.0		+3.1			
Tiefste Temperatur am Erdboden	°C	1951-1980	-11.3	*-11.9*	-4.9	01.	*+7.0*		-24.0*	21.12.1969
Mittlere Min.-Temperatur am Erdboden	°C	1951-1980	-1.9	*-2.1*	0.2		*+2.3*			
Heiße Tage (Max.: ≥ 30,0°C)	Tage	1909-1969	0	0	0		0			
Sommertage (Max.: ≥ 25,0°C)	Tage	1909-1969	0	0	0		0			
(Max.: ≥ 20,0°C)	Tage			0	0		0			
Frosttage (Min.: < 0,0°C)	Tage	1909-1969	17.4	17.0	4		-13			
(Min.: ≤ -10,0°C)	Tage			1.6	0		-2			
Eistage (Max.: < 0,0°C)	Tage	1909-1969	6.3	7.2	0		-7			
Zahl d. Tage Min-Temp. am Erdb. < 0,0°C	Tage	1951-1980	19.4	19.9	15		-5			
Sonnenscheindauer	h	1951-1980	35.7	37.4	25.7		-11.7	69		
in % vom astronomisch möglichen	%		14.9	15.6	10.7		-4.9			
Zahl der sonnenscheinlosen Tage	Tage	1951-1980	16.5	16.3	8		-8			
Bewölkung (in Achtel)	/8	1909-1969	6.2	6.2	6.5		+0.3			
Heitere Tage (< 1,6 Achtel Bewölkung)	Tage	1909-1969	1.5	2.0	0		-2			
Trübe Tage (> 6,4 Achtel Bewölkung)	Tage	1909-1969	17.7	18.4	18		-0			
Dampfdruck	hPa	1909-1969	6.0	6.1	6.9		+0.8			
Relative Luftfeuchtigkeit	%	1909-1969	88	86	82		-4			
Niederschlagshöhe	mm	1909-1969	47.4	54.5	78.6		+24.1	144		
Maximale Tagesmenge	mm	1909-1969	10.3	10.8	22.4	16.	+11.6		23.3	10.12.1990
Zahl der Tage mit Sturzregen	Tage			0	-		-			
Zahl der Tage mit ≥ 10,0 mm Niederschlag	Tage	1909-1969	0.9	0.9	2		+1			
Zahl der Tage mit ≥ 5,0 mm Niederschlag	Tage			4.1	4		-0			
Zahl der Tage mit ≥ 2,5 mm Niederschlag	Tage	1909-1969	6.5	7.2	10		+3			
Zahl der Tage mit ≥ 1,0 mm Niederschlag	Tage	1909-1969	10.1	11.2	16		+5			
Zahl der Tage mit ≥ 0,1 mm Niederschlag	Tage	1909-1969	16.0	18.2	25		+7			
Z.d.T.m. gefall. flüss. Niederschl. ≥ 0,1 mm	Tage			9.0	15		+6			
Z.d.T.m. flüss. u. fest. Niederschl. ≥ 0,1 mm	Tage			5.3	10		+5			
Z.d.T.m. gefall. fest. Niederschl. ≥ 0,1 mm	Tage			3.7	0		-4			
Z.d.T.m. abgesetzt. Niederschl. ≥ 0,1 mm	Tage			0.2	-		-			
Z.d.T.m. Hagel ≥ 0,0 mm	Tage			0.1	0		-0			
Z.d.T.m. Graupel, Griesel o. Eisk. ≥ 0,0 mm	Tage			5.9	6		+0			
Zahl der Tage mit Reif oder Rauhreif	Tage	1964-1980	13.1	14.0	-		-			
Zahl der Tage mit Tau	Tage	1964-1980	6.3	6.8	-		-			
Zahl der Tage mit Nebel	Tage	1951-1980	6.3	4.9	2		-3			
Zahl der Tage mit Gewitter	Tage	1951-1980	0.3	0.3	0		-0			
Zahl der Tage mit Wetterleuchten	Tage	1951-1980	0.0	0.0	0		-0			
Z.d.T.m. Schneedecke ≥ 0 cm um 7:30 Uhr	Tage	1951-1980	8.8	9.4	0		-9			
Z.d.T.m. Schneedecke ≥ 1 cm um 7:30 Uhr	Tage	1951-1980	7.5	8.2	0		-8			
Z.d.T.m. Schneedecke ≥ 5 cm um 7:30 Uhr	Tage			4.3	0		-4			
Z.d.T.m. Neuschnee ≥ 1 cm um 7:30 Uhr	Tage			3.7	0		-4			
Summe der um 7:30 gem. Neuschneemenge	cm									
Max. Höhe der Schneedecke um 7:30 Uhr	cm			6.7	.		-7		43	28.12.2010
Z.d.T.m. Glatteis durch gefrierenden Regen	Tage	1951-1980	1.5	1.7	1		-1			
Z.d.T.m. Böen Windst. 6 Bft. (≥ 10,8 m/s)	Tage	1952-1980		13.8	26		+12			
Z.d.T.m. Böen Windst. 8 Bft. (≥ 17,2 m/s)	Tage	1952-1980		2.3	8		+6			
Maximale Windspitze	m/s			21.2	22.5	29.	+1.3		33.0*	9.12.1993
Heizgradsumme					522.6					
Kältesumme		1909-1969	39.9	40.4	0.0		-40.4			

*) 1951-2010 +) 1881-2010

Manfred Wegener

Beiträge zur Berliner Wetterkarte

Klimatologische Mittelwerte von Berlin-Dahlem

10/12 ISSN 0177-3984
KBD 01/12 01.02.2012

Element	Maß-einheit	Beob-achtungs-periode	Vieljähr. Durch-schnitts-wert	Durch-schnitts-wert 1961-90	**JANUAR 2012**	Datum	Abweichung vom Durchschnitt 1961-90	in %	Bisherige Extreme 1909-2011	Datum
Luftdruck auf NN reduziert	hPa	1881-1970	1016.8	1016.2	1016.7		+0.5			
Höchster Luftdruck	hPa	1951-1980	1036.8	1036.7	1035.7	31.	-1.0		1057.8+	23.01.1907
Tiefster Luftdruck	hPa	1951-1980	989.7	989.9	976.3	05.	-13.6		966.6+	17.01.1955
Temperatur (stündl. Ablesung 01-24 MEZ)	°C	1955-1984	-0.5	-0.5	1.7		+2.2			
Temperatur (Klimamittel)	°C	1909-1969	-0.4	-0.4	1.7		+2.1			
Höchste Temperatur	°C	1909-1969	8.8	8.3	12.2	02.	+3.9		15.2	10.01.1991<br>28.01.2002
Mittlere Maximum-Temperatur	°C	1909-1969	1.9	1.8	3.9		+2.1			
Tiefste Temperatur	°C	1909-1969	-11.8	-11.4	-10.8	31.	+0.6		-21.0	26.01.1942
Mittlere Minimum-Temperatur	°C	1909-1969	-3.1	-2.9	-0.5		+2.4			
Tiefste Temperatur am Erdboden	°C	1951-1980	-14.5	*-14.0*	-12.1	31.	*+1.9*		-26.6*	31.01.1963
Mittlere Min.-Temperatur am Erdboden	°C	1951-1980	-4.2	*-4.1*	-2.0		*+2.1*			
Heiße Tage (Max.: ≥ 30,0°C)	Tage	1909-1969	0	0	0		0			
Sommertage (Max.: ≥ 25,0°C)	Tage	1909-1969	0	0	0		0			
(Max.: ≥ 20,0°C)	Tage			0	0		0			
Frosttage (Min.: < 0,0°C)	Tage	1909-1969	20.5	19.9	14		-6			
(Min.: ≤ -10,0°C)	Tage			4.0	1		-3			
Eistage (Max.: < 0,0°C)	Tage	1909-1969	9.4	9.2	5		-4			
Zahl d. Tage Min-Temp. am Erdb. < 0,0°C	Tage	1951-1980	24.1	23.1	17		-6			
Sonnenscheindauer	h	1951-1980	48.0	45.4	59.1		+13.7	130		
in % vom astronomisch möglichen	%		18.7	17.7	23.0		+5.3			
Zahl der sonnenscheinlosen Tage	Tage	1951-1980	14.4	13.9	15		+1			
Bewölkung (in Achtel)	/8	1909-1969	6.0	6.0	6.0		0.0			
Heitere Tage (< 1,6 Achtel Bewölkung)	Tage	1909-1969	2.1	1.9	2		+0			
Trübe Tage (> 6,4 Achtel Bewölkung)	Tage	1909-1969	17.4	17.4	19		+2			
Dampfdruck	hPa	1909-1969	5.3	5.4	5.9		+0.5			
Relative Luftfeuchtigkeit	%	1909-1969	85	85	81		-4			
Niederschlagshöhe	mm	1909-1969	46.6	43.2	57.2		+14.0	132		
Maximale Tagesmenge	mm	1909-1969	11.2	9.3	9.4	19.	+0.1		45.0	18.01.2007
Zahl der Tage mit Sturzregen	Tage			0	-		-			
Zahl der Tage mit ≥ 10,0 mm Niederschlag	Tage	1909-1969	0.8	0.4	0		-0			
Zahl der Tage mit ≥ 5,0 mm Niederschlag	Tage			2.7	4		+1			
Zahl der Tage mit ≥ 2,5 mm Niederschlag	Tage	1909-1969	6.4	6.4	10		+4			
Zahl der Tage mit ≥ 1,0 mm Niederschlag	Tage	1909-1969	10.6	10.3	14		+4			
Zahl der Tage mit ≥ 0,1 mm Niederschlag	Tage	1909-1969	17.5	17.8	18		+0			
Z.d.T.m. gefall. flüss. Niederschl. ≥ 0,1 mm	Tage			6.9	11		+4			
Z.d.T.m. flüss. u. fest. Niederschl. ≥ 0,1 mm	Tage			6.4	6		-0			
Z.d.T.m. gefall. fest. Niederschl. ≥ 0,1 mm	Tage			4.5	1		-4			
Z.d.T.m. abgesetzt. Niederschl. ≥ 0,1 mm	Tage			0.1	-		-			
Z.d.T.m. Hagel ≥ 0,0 mm	Tage			0.1	0		-0			
Z.d.T.m. Graupel, Griesel o. Eisk. ≥ 0,0 mm	Tage		5.0	6.3	6		-0			
Zahl der Tage mit Reif oder Rauhreif	Tage	1964-1980	15.1	14.4	-		-			
Zahl der Tage mit Tau	Tage	1964-1980	4.8	5.1	-		-			
Zahl der Tage mit Nebel	Tage	1951-1980	5.4	5.3	3		-2			
Zahl der Tage mit Gewitter	Tage	1951-1980	0.3	0.5	1		+0			
Zahl der Tage mit Wetterleuchten	Tage	1951-1980	0.1	0.1	0		-0			
Z.d.T.m. Schneedecke ≥ 0 cm um 7:30 Uhr	Tage	1951-1980	16.1	16.3	4		-12			
Z.d.T.m. Schneedecke ≥ 1 cm um 7:30 Uhr	Tage	1951-1980	15.1	15.3	3		-12			
Z.d.T.m. Schneedecke ≥ 5 cm um 7:30 Uhr	Tage			9.4	0		-9			
Z.d.T.m. Neuschnee ≥ 1 cm um 7:30 Uhr	Tage			4.8	1		-4			
Summe der um 7:30 gem. Neuschneemenge	cm				1					
Max. Höhe der Schneedecke um 7:30 Uhr	cm			9.5	1	9./30./31	-9		32	01.01.2011
Z.d.T.m. Glatteis durch gefrierenden Regen	Tage	1951-1980	2.9	2.8	0		-3			
Z.d.T.m. Böen Windst. 6 Bft. (≥ 10,8 m/s)	Tage	1952-1980		12.0	23		+11			
Z.d.T.m. Böen Windst. 8 Bft. (≥ 17,2 m/s)	Tage	1952-1980		3.0	8		+5			
Maximale Windspitze	m/s			21.2	23.3	12.	+2.1		34.5*	18.1.2007
Heizgradsumme					602.6					
Kältesumme		1909-1969	66.2	66.6	34.8		-31.8			

*) 1951-2011 +) 1881-2011

Manfred Wegener

Beiträge zur Berliner Wetterkarte

Klimatologische Mittelwerte von Berlin-Dahlem

18/12 ISSN 0177-3984
KBD II/12 01.03.2012

Element	Maß-einheit	Beob-achtungs-periode	Vieljähr. Durch-schnitts-wert	Durch-schnitts-wert 1961-90	**Februar 2012**	Datum	Abweichung vom Durchschnitt 1961-90	in %	Bisherige Extreme 1909-2011	Datum
Luftdruck auf NN reduziert	hPa	1881-1970	1016.1	1016.2	1025.7		+9.5			
Höchster Luftdruck	hPa	1951-1980	1035.7	1036.2	1043.3	10.	+7.1		1048.0+	16.02.1959
Tiefster Luftdruck	hPa	1951-1980	990.1	989.6	990.9	15.	+1.3		965.2+	26.02.1989
Temperatur (stündl. Ablesung 01-24 MEZ)	°C	1955-1984	0.2	0.5	-1.8		-2.3			
Temperatur (Klimamittel)	°C	1909-1969	0.1	0.6	-1.8		-2.4			
Höchste Temperatur	°C	1909-1969	10.4	10.0	11.4	24.	+1.4		18.6	21.02.1990
Mittlere Maximum-Temperatur	°C	1909-1969	3.1	3.5	1.3		-2.2			
Tiefste Temperatur	°C	1909-1969	-11.4	-9.4	-19.3	06.	-9.9		-26.0	11.02.1929
Mittlere Minimum-Temperatur	°C	1909-1969	-2.9	-2.2	-5.4		-3.2			
Tiefste Temperatur am Erdboden	°C	1951-1980	-14.1	*-12.3*	-24.0	06.	*-11.7*		-29.2*	09.02.1956
Mittlere Min.-Temperatur am Erdboden	°C	1951-1980	-4.1	*-3.6*	-8.2		*-4.6*			
Heiße Tage (Max.: ≥ 30,0°C)	Tage	1909-1969	0	0	0		0			
Sommertage (Max.: ≥ 25,0°C)	Tage	1909-1969	0	0	0		0			
(Max.: ≥ 20,0°C)	Tage			0	0		0			
Frosttage (Min.: < 0,0°C)	Tage	1909-1969	18.5	17.6	19		+1			
(Min.: ≤ -10,0°C)	Tage			2.1	11		+9			
Eistage (Max.: < 0,0°C)	Tage	1909-1969	6.8	5.9	13		+7			
Zahl d. Tage Min-Temp. am Erdb. < 0,0°C	Tage	1951-1980	21.5	21.1	22		+1			
Sonnenscheindauer	h	1951-1980	69.3	72.3	105.7		+33.4	146		
in % vom astronomisch möglichen	%		25.1	26.2	38.3		+12.1			
Zahl der sonnenscheinlosen Tage	Tage	1951-1980	10.0	10.0	5		-5			
Bewölkung (in Achtel)	/8	1909-1969	5.8	5.6	5.4		-0.2			
Heitere Tage (< 1,6 Achtel Bewölkung)	Tage	1909-1969	2.2	3.1	2		-1			
Trübe Tage (> 6,4 Achtel Bewölkung)	Tage	1909-1969	14.3	14.1	13		-1			
Dampfdruck	hPa	1909-1969	5.3	5.5	4.8		-0.7			
Relative Luftfeuchtigkeit	%	1909-1969	82	82	77		-5			
Niederschlagshöhe	mm	1909-1969	36.8	36.6	35.4		-1.2	97		
Maximale Tagesmenge	mm	1909-1969	9.6	9.9	7.8	16.	-2.1		24.2	06.02.1974
Zahl der Tage mit Sturzregen	Tage			0	-		-			
Zahl der Tage mit ≥ 10,0 mm Niederschlag	Tage	1909-1969	0.7	0.6	0		-1			
Zahl der Tage mit ≥ 5,0 mm Niederschlag	Tage			2.1	2		-0			
Zahl der Tage mit ≥ 2,5 mm Niederschlag	Tage	1909-1969	4.6	4.7	7		+2			
Zahl der Tage mit ≥ 1,0 mm Niederschlag	Tage	1909-1969	8.5	8.5	9		+0			
Zahl der Tage mit ≥ 0,1 mm Niederschlag	Tage	1909-1969	14.1	14.3	18		+4			
Z.d.T.m. gefall. flüss. Niederschl. ≥ 0,1 mm	Tage			4.8	9		+4			
Z.d.T.m. flüss. u. fest. Niederschl. ≥ 0,1 mm	Tage			5.8	4		-2			
Z.d.T.m. gefall. fest. Niederschl. ≥ 0,1 mm	Tage			3.6	3		-1			
Z.d.T.m. abgesetzt. Niederschl. ≥ 0,1 mm	Tage			0.0	-		-			
Z.d.T.m. Hagel ≥ 0,0 mm	Tage			0.2	0		-0			
Z.d.T.m. Graupel, Griesel o. Eisk. ≥ 0,0 mm	Tage			6.1	3		-3			
Zahl der Tage mit Reif oder Rauhreif	Tage	1964-1980	13.8	13.6	-		-			
Zahl der Tage mit Tau	Tage	1964-1980	5.4	5.6	-		-			
Zahl der Tage mit Nebel	Tage	1951-1980	4.8	4.2	1		-3			
Zahl der Tage mit Gewitter	Tage	1951-1980	0.4	0.7	0		-1			
Zahl der Tage mit Wetterleuchten	Tage	1951-1980	0.1	0.1	0		-0			
Z.d.T.m. Schneedecke ≥ 0 cm um 7:30 Uhr	Tage	1951-1980	13.0	12.1	17		+5			
Z.d.T.m. Schneedecke ≥ 1 cm um 7:30 Uhr	Tage	1951-1980	12.0	10.8	17		+6			
Z.d.T.m. Schneedecke ≥ 5 cm um 7:30 Uhr	Tage			6.5	3		-4			
Z.d.T.m. Neuschnee ≥ 1 cm um 7:30 Uhr	Tage			3.7	2		-2			
Summe der um 7:30 gem. Neuschneemenge	cm				4					
Max. Höhe der Schneedecke um 7:30 Uhr	cm			9.9	5	11./13./14	-5		44	16.02.1940
Z.d.T.m. Glatteis durch gefrierenden Regen	Tage	1951-1980	1.1	1.3	1		-0			
Z.d.T.m. Böen Windst. 6 Bft. (≥ 10,8 m/s)	Tage	1952-1980		11.0	15		+4			
Z.d.T.m. Böen Windst. 8 Bft. (≥ 17,2 m/s)	Tage	1952-1980		2.1	5		+3			
Maximale Windspitze	m/s			20.2	21.2	15.	+1.0		35.5*	21.2.1967
Heizgradsumme					650.2					
Kältesumme		1909-1969	52.1	38.7	120.1		+81.4			

*) 1951-2011 +) 1881-2011

Manfred Wegener

Beiträge zur Berliner Wetterkarte

Klimatologische Mittelwerte von Berlin-Dahlem

23/12 ISSN 0177-3984
KBD Iii/12 03.04.2012

Element	Maß-einheit	Beob-achtungs-periode	Vieljähr. Durch-schnitts-wert	Durch-schnitts-wert 1961-90	**Maerz 2012**	Datum	Abweichung vom Durchschnitt 1961-90	in %	Bisherige Extreme 1909-2011	Datum
Luftdruck auf NN reduziert	hPa	1881-1970	1014.6	1015.1	1024.8		+9.7			
Höchster Luftdruck	hPa	1951-1980	1033.3	1033.2	1036.1	09.	+2.9		1044.7*	15.03.2003
Tiefster Luftdruck	hPa	1951-1980	993.6	990.6	999.6	31.	+9.0		972.7*	01.03.1990
Temperatur (stündl. Ablesung 01-24 MEZ)	°C	1955-1984	3.8	4.0	7.4		+3.4			
Temperatur (Klimamittel)	°C	1909-1969	3.7	4.0	7.4		+3.4			
Höchste Temperatur	°C	1909-1969	16.4	16.6	20.2	16.	+3.6		25.1	30.03.1968
Mittlere Maximum-Temperatur	°C	1909-1969	7.8	7.9	12.1		+4.2			
Tiefste Temperatur	°C	1909-1969	-7.0	-6.0	-4.2	07.	+1.8		-16.5	06.03.1942
Mittlere Minimum-Temperatur	°C	1909-1969	-0.2	0.5	3.0		+2.5			
Tiefste Temperatur am Erdboden	°C	1951-1980	-9.3	-8.8	-6.7	07.	+2.1		-21.6*	08.03.1965
Mittlere Min.-Temperatur am Erdboden	°C	1951-1980	-1.5	*-1.1*	0.0		*+1.1*			
Heiße Tage (Max.: ≥ 30,0°C)	Tage	1909-1969	0	0	0		0			
Sommertage (Max.: ≥ 25,0°C)	Tage	1909-1969	0.0	0.0	0		-0			
(Max.: ≥ 20,0°C)	Tage			0.3	1		+1			
Frosttage (Min.: < 0,0°C)	Tage	1909-1969	15.1	12.6	7		-6			
(Min.: ≤ -10,0°C)	Tage			0.3	0		-0			
Eistage (Max.: < 0,0°C)	Tage	1909-1969	1.7	1.3	0		-1			
Zahl d. Tage Min-Temp. am Erdb. < 0,0°C	Tage	1951-1980	19.5	18.1	17		-1			
Sonnenscheindauer	h	1951-1980	134.2	122.1	175.7		+53.6	144		
in % vom astronomisch möglichen	%		36.6	33.3	47.9		+14.6			
Zahl der sonnenscheinlosen Tage	Tage	1951-1980	5.8	5.9	5		-1			
Bewölkung (in Achtel)	/8	1909-1969	5.1	5.3	4.7		-0.6			
Heitere Tage (< 1,6 Achtel Bewölkung)	Tage	1909-1969	3.8	3.3	5		+2			
Trübe Tage (> 6,4 Achtel Bewölkung)	Tage	1909-1969	11.7	13.0	10		-3			
Dampfdruck	hPa	1909-1969	6.1	6.3	7.1		+0.8			
Relative Luftfeuchtigkeit	%	1909-1969	76	75	69		-6			
Niederschlagshöhe	mm	1909-1969	33.9	37.5	7.8		-29.7	21		
Maximale Tagesmenge	mm	1909-1969	10.2	9.4	2.7	31.	-6.7		30.7	06.03.1915
Zahl der Tage mit Sturzregen	Tage			0	-		-			
Zahl der Tage mit ≥ 10,0 mm Niederschlag	Tage	1909-1969	0.5	0.6	0		-1			
Zahl der Tage mit ≥ 5,0 mm Niederschlag	Tage			2.1	0		-2			
Zahl der Tage mit ≥ 2,5 mm Niederschlag	Tage	1909-1969	4.4	5.2	1		-4			
Zahl der Tage mit ≥ 1,0 mm Niederschlag	Tage	1909-1969	7.8	8.4	3		-5			
Zahl der Tage mit ≥ 0,1 mm Niederschlag	Tage	1909-1969	13.2	15.5	9		-7			
Z.d.T.m. gefall. flüss. Niederschl. ≥ 0,1 mm	Tage			7.7	8		+0			
Z.d.T.m. flüss. u. fest. Niederschl. ≥ 0,1 mm	Tage			5.1	0		-5			
Z.d.T.m. gefall. fest. Niederschl. ≥ 0,1 mm	Tage			2.5	0		-3			
Z.d.T.m. abgesetzt. Niederschl. ≥ 0,1 mm	Tage			0.1	-		-			
Z.d.T.m. Hagel ≥ 0,0 mm	Tage			0.2	0		-0			
Z.d.T.m. Graupel, Griesel o. Eisk. ≥ 0,0 mm	Tage			4.5	1		-4			
Zahl der Tage mit Reif oder Rauhreif	Tage	1964-1980	12.0	11.2	-		-			
Zahl der Tage mit Tau	Tage	1964-1980	10.1	10.5	-		-			
Zahl der Tage mit Nebel	Tage	1951-1980	2.0	1.6	1		-1			
Zahl der Tage mit Gewitter	Tage	1951-1980	0.9	0.9	1		+0			
Zahl der Tage mit Wetterleuchten	Tage	1951-1980	0.1	0.0	0		-0			
Z.d.T.m. Schneedecke ≥ 0 cm um 7:30 Uhr	Tage	1951-1980	6.9	6.8	0		-7			
Z.d.T.m. Schneedecke ≥ 1 cm um 7:30 Uhr	Tage	1951-1980	5.7	5.0	0		-5			
Z.d.T.m. Schneedecke ≥ 5 cm um 7:30 Uhr	Tage			3.1	0		-3			
Z.d.T.m. Neuschnee ≥ 1 cm um 7:30 Uhr	Tage			1.9	0		-2			
Summe der um 7:30 gem. Neuschneemenge	cm									
Max. Höhe der Schneedecke um 7:30 Uhr	cm			7.3			-7		49	06.03.1970
Z.d.T.m. Glatteis durch gefrierenden Regen	Tage	1951-1980	0.3	0.1	0		-0			
Z.d.T.m. Böen Windst. 6 Bft. (≥ 10,8 m/s)	Tage	1952-1980		15.4	19		+4			
Z.d.T.m. Böen Windst. 8 Bft. (≥ 17,2 m/s)	Tage	1952-1980		3.4	4		+1			
Maximale Windspitze	m/s			21.6	24.0	31.	+2.4		30.9*	28.3.1997
Heizgradsumme					410.5					
Kältesumme		1909-1969	12.1	10.3	0.0		-10.3			

*) 1951-2011 +) 1881-2011

Manfred Wegener

Beiträge zur Berliner Wetterkarte

Klimatologische Mittelwerte von Berlin-Dahlem

29/12
KBD 04/12

ISSN 0177-3984
02.05.2012

Element	Maß-einheit	Beob-achtungs-periode	Vieljähr. Durch-schnitts-wert	Durch-schnitts-wert 1961-90	**April 2012**	Datum	Abweichung vom Durchschnitt 1961-90	in %	Bisherige Extreme 1909-2011	Datum
Luftdruck auf NN reduziert	hPa	1881-1970	1013.5	1013.8	1006.6		-7.2			
Höchster Luftdruck	hPa	1951-1980	1030.0	1029.7	1020.0	05.	-9.7		1037.5*	21.04.2002
Tiefster Luftdruck	hPa	1951-1980	995.4	995.6	994.1	19./20.	-1.5		983.5*	07.04.1959
Temperatur (stündl. Ablesung 01-24 MEZ)	°C	1955-1984	8.1	8.3	9.4		+1.1			
Temperatur (Klimamittel)	°C	1909-1969	8.6	8.4	9.4		+1.0			
Höchste Temperatur	°C	1909-1969	22.6	22.6	30.7	28.	+8.1		30.9	22.04.1968
Mittlere Maximum-Temperatur	°C	1909-1969	13.4	13.1	14.6		+1.5			
Tiefste Temperatur	°C	1909-1969	-2.3	-2.2	-2.8	06.	-0.6		-6.7	04.04.1929
Mittlere Minimum-Temperatur	°C	1909-1969	3.8	3.9	4.0		+0.1			
Tiefste Temperatur am Erdboden	°C	1951-1980	-4.5	-4.1	-5.6	08./09.	-1.5		-8.1*	08.04.1956
Mittlere Min.-Temperatur am Erdboden	°C	1951-1980	2.0	*2.2*	0.6		*-1.6*			
Heiße Tage (Max.: ≥ 30,0°C)	Tage	1909-1969	0.1	0.1	1		+1			
Sommertage (Max.: ≥ 25,0°C)	Tage	1909-1969	0.6	0.4	2		+2			
(Max.: ≥ 20,0°C)	Tage			2.6	5		+2			
Frosttage (Min.: < 0,0°C)	Tage	1909-1969	4.1	3.4	7		+4			
(Min.: ≤ -10,0°C)	Tage			0	0		0			
Eistage (Max.: < 0,0°C)	Tage	1909-1969	0	0	0		0			
Zahl d. Tage Min-Temp. am Erdb. < 0,0°C	Tage	1951-1980	9.2	8.8	14		+5			
Sonnenscheindauer	h	1951-1980	165.6	157.7	171.1		+13.4	109		
in % vom astronomisch möglichen	%		39.8	37.9	41.1		+3.2			
Zahl der sonnenscheinlosen Tage	Tage	1951-1980	3.5	3.8	1		-3			
Bewölkung (in Achtel)	/8	1909-1969	5.0	5.0	5.3		+0.3			
Heitere Tage (< 1,6 Achtel Bewölkung)	Tage	1909-1969	3.2	3.2	0		-3			
Trübe Tage (> 6,4 Achtel Bewölkung)	Tage	1909-1969	9.4	10.3	9		-1			
Dampfdruck	hPa	1909-1969	7.7	7.5	7.7		+0.2			
Relative Luftfeuchtigkeit	%	1909-1969	70	69	66		-3			
Niederschlagshöhe	mm	1909-1969	42.8	42.2	28.2		-14.0	67		
Maximale Tagesmenge	mm	1909-1969	10.7	10.4	11.3	24.	+0.9		31.9	12.04.1949
Zahl der Tage mit Sturzregen	Tage			0	-		-			
Zahl der Tage mit ≥ 10,0 mm Niederschlag	Tage	1909-1969	0.8	0.9	1		+0			
Zahl der Tage mit ≥ 5,0 mm Niederschlag	Tage			2.7	2		-1			
Zahl der Tage mit ≥ 2,5 mm Niederschlag	Tage	1909-1969	5.8	5.4	3		-2			
Zahl der Tage mit ≥ 1,0 mm Niederschlag	Tage	1909-1969	9.1	8.9	7		-2			
Zahl der Tage mit ≥ 0,1 mm Niederschlag	Tage	1909-1969	13.8	14.2	11		-3			
Z.d.T.m. gefall. flüss. Niederschl. ≥ 0,1 mm	Tage			10.2	9		-1			
Z.d.T.m. flüss. u. fest. Niederschl. ≥ 0,1 mm	Tage			3.7	2		-2			
Z.d.T.m. gefall. fest. Niederschl. ≥ 0,1 mm	Tage			0.3	0		-0			
Z.d.T.m. abgesetzt. Niederschl. ≥ 0,1 mm	Tage			0.0	-		-			
Z.d.T.m. Hagel ≥ 0,0 mm	Tage			0.3	0		-0			
Z.d.T.m. Graupel, Griesel o. Eisk. ≥ 0,0 mm	Tage			2.5	2		-1			
Zahl der Tage mit Reif oder Rauhreif	Tage	1964-1980	6.0	5.1	-		-			
Zahl der Tage mit Tau	Tage	1964-1980	19.9	19.3	-		-			
Zahl der Tage mit Nebel	Tage	1951-1980	1.4	1.0	0		-1			
Zahl der Tage mit Gewitter	Tage	1951-1980	1.9	2.2	1		-1			
Zahl der Tage mit Wetterleuchten	Tage	1951-1980	0.3	0.3	0		-0			
Z.d.T.m. Schneedecke ≥ 0 cm um 7:30 Uhr	Tage	1951-1980	0.5	0.4	0		-0			
Z.d.T.m. Schneedecke ≥ 1 cm um 7:30 Uhr	Tage	1951-1980	0.4	0.3	0		-0			
Z.d.T.m. Schneedecke ≥ 5 cm um 7:30 Uhr	Tage			0.1	0		-0			
Z.d.T.m. Neuschnee ≥ 1 cm um 7:30 Uhr	Tage			0.3	0		-0			
Summe der um 7:30 gem. Neuschneemenge	cm									
Max. Höhe der Schneedecke um 7:30 Uhr	cm			1.1			-1		14	11.04.1977
Z.d.T.m. Glatteis durch gefrierenden Regen	Tage	1951-1980	0	0	0		0			
Z.d.T.m. Böen Windst. 6 Bft. (≥ 10,8 m/s)	Tage	1952-1980		13.4	20		+7			
Z.d.T.m. Böen Windst. 8 Bft. (≥ 17,2 m/s)	Tage	1952-1980		1.9	3		+1			
Maximale Windspitze	m/s			20.0	20.3	01.	+0.3		25.8*	19.4.1980
Heizgradsumme					331.0					
Kältesumme		1909-1969	0.3	0.1	0.0		-0.1			

*) 1951-2011 +) 1881-2011

Manfred Wegener

Beiträge zur Berliner Wetterkarte

Klimatologische Mittelwerte von Berlin-Dahlem

34/12
KBD V/12

ISSN 0177-3984
01.06.2012

Element	Maß-einheit	Beob-achtungs-periode	Vieljähr. Durch-schnitts-wert	Durch-schnitts-wert 1961-90	**MAI 2012**	Datum	Abweichung vom Durchschnitt 1961-90	in %	Bisherige Extreme 1909-2011	Datum
Luftdruck auf NN reduziert	hPa	1881-1970	1015.1	1015.2	1015.9		+0.7			
Höchster Luftdruck	hPa	1951-1980	1028.3	1028.1	1030.9	12.	+2.8		1037.5*	02.05.1990
Tiefster Luftdruck	hPa	1951-1980	1000.7	999.5	1001.7	04.	+2.2		984.4*	06.05.1997
Temperatur (stündl. Ablesung 01-24 MEZ)	°C	1955-1984	13.1	13.4	15.4		+2.0			
Temperatur (Klimamittel)	°C	1909-1969	13.6	13.5	15.4		+1.9			
Höchste Temperatur	°C	1909-1969	27.1	27.0	30.5	22.	+3.5		33.3	28.05.2005
Mittlere Maximum-Temperatur	°C	1909-1969	18.7	18.6	21.0		+2.4			
Tiefste Temperatur	°C	1909-1969	1.1	1.7	1.2	14.	-0.5		-2.9	09.05.1941
Mittlere Minimum-Temperatur	°C	1909-1969	8.0	8.2	9.2		+1.0			
Tiefste Temperatur am Erdboden	°C	1951-1980	-0.4	0.8	-1.9	14.	-2.7		-4.1*	12.05.1978
Mittlere Min.-Temperatur am Erdboden	°C	1951-1980	6.4	*6.7*	5.7		*-1.0*			
Heiße Tage (Max.: ≥ 30,0°C)	Tage	1909-1969	0.4	0.2	2		+2			
Sommertage (Max.: ≥ 25,0°C)	Tage	1909-1969	3.4	3.3	9		+6			
(Max.: ≥ 20,0°C)	Tage			11.9	18		+6			
Frosttage (Min.: < 0,0°C)	Tage	1909-1969	0.3	0.2	0		-0			
(Min.: ≤ -10,0°C)	Tage			0	0		0			
Eistage (Max.: < 0,0°C)	Tage	1909-1969	0	0	0		0			
Zahl d. Tage Min-Temp. am Erdb. < 0,0°C	Tage	1951-1980	1.6	1.0	4		+3			
Sonnenscheindauer	h	1951-1980	221.4	221.6	250.1		+28.5	113		
in % vom astronomisch möglichen	%		45.4	45.5	51.4		+5.9			
Zahl der sonnenscheinlosen Tage	Tage	1951-1980	2.0	2.1	1		-1			
Bewölkung (in Achtel)	/8	1909-1969	4.9	4.7	4.6		-0.1			
Heitere Tage (< 1,6 Achtel Bewölkung)	Tage	1909-1969	3.6	3.6	3		-1			
Trübe Tage (> 6,4 Achtel Bewölkung)	Tage	1909-1969	8.8	8.3	7		-1			
Dampfdruck	hPa	1909-1969	10.4	10.3	10.8		+0.5			
Relative Luftfeuchtigkeit	%	1909-1969	67	67	63		-4			
Niederschlagshöhe	mm	1909-1969	49.0	55.3	44.3		-11.0	80		
Maximale Tagesmenge	mm	1909-1969	15.9	15.3	16.2	31.	+0.9		50.3	03.05.1996
Zahl der Tage mit Sturzregen	Tage			0.1	-		-			
Zahl der Tage mit ≥ 10,0 mm Niederschlag	Tage	1909-1969	1.3	1.4	2		+1			
Zahl der Tage mit ≥ 5,0 mm Niederschlag	Tage			4.0	3		-1			
Zahl der Tage mit ≥ 2,5 mm Niederschlag	Tage	1909-1969	5.4	6.4	5		-1			
Zahl der Tage mit ≥ 1,0 mm Niederschlag	Tage	1909-1969	8.5	9.6	7		-3			
Zahl der Tage mit ≥ 0,1 mm Niederschlag	Tage	1909-1969	12.6	14.0	11		-3			
Z.d.T.m. gefall. flüss. Niederschl. ≥ 0,1 mm	Tage			13.0	11		-2			
Z.d.T.m. flüss. u. fest. Niederschl. ≥ 0,1 mm	Tage			1.0	0		-1			
Z.d.T.m. gefall. fest. Niederschl. ≥ 0,1 mm	Tage			0	0		0			
Z.d.T.m. abgesetzt. Niederschl. ≥ 0,1 mm	Tage			0.0	-		-			
Z.d.T.m. Hagel ≥ 0,0 mm	Tage			0.4	0		-0			
Z.d.T.m. Graupel, Griesel o. Eisk. ≥ 0,0 mm	Tage			0.5	0		-1			
Zahl der Tage mit Reif oder Rauhreif	Tage	1964-1980	0	0	-		-			
Zahl der Tage mit Tau	Tage	1964-1980	23.2	23.3	-		-			
Zahl der Tage mit Nebel	Tage	1951-1980	0.7	0.6	0		-1			
Zahl der Tage mit Gewitter	Tage	1951-1980	4.4	5.0	3		-2			
Zahl der Tage mit Wetterleuchten	Tage	1951-1980	0.8	1.0	5		+4			
Z.d.T.m. Schneedecke ≥ 0 cm um 7:30 Uhr	Tage	1951-1980	0	0	0		0			
Z.d.T.m. Schneedecke ≥ 1 cm um 7:30 Uhr	Tage	1951-1980	0	0	0		0			
Z.d.T.m. Schneedecke ≥ 5 cm um 7:30 Uhr	Tage			0	0		0			
Z.d.T.m. Neuschnee ≥ 1 cm um 7:30 Uhr	Tage			0	0		0			
Summe der um 7:30 gem. Neuschneemenge	cm									
Max. Höhe der Schneedecke um 7:30 Uhr	cm			0.0			0		0 dbr	01.05.1970
Z.d.T.m. Glatteis durch gefrierenden Regen	Tage	1951-1980	0	0	0		0			
Z.d.T.m. Böen Windst. 6 Bft. (≥ 10,8 m/s)	Tage	1952-1980		13.0	18		+5			
Z.d.T.m. Böen Windst. 8 Bft. (≥ 17,2 m/s)	Tage	1952-1980		1.2	0		-1			
Maximale Windspitze	m/s			19.2	16.7	12.	-2.5		28.4*	16.5.1983
Heizgradsumme					55.9					
Kältesumme		1909-1969	0	0	0		0			

*) 1951-2011 +) 1881-2011

Manfred Wegener

Beiträge zur Berliner Wetterkarte

Klimatologische Mittelwerte von Berlin-Dahlem

39/12 ISSN 0177-3984
KBD VI/12 03.07.2012

Element	Maß-einheit	Beob-achtungs-periode	Vieljähr. Durch-schnitts-wert	Durch-schnitts-wert 1961-90	Juni 2012	Datum	Abweichung vom Durchschnitt 1961-90	in %	Bisherige Extreme 1909-2011	Datum
Luftdruck auf NN reduziert	hPa	1881-1970	1015.1	1015.3	1013.0		-2.3			
Höchster Luftdruck	hPa	1951-1980	1026.9	1026.4	1022.6	19.	-3.8		1035.0*	13.06.1957
Tiefster Luftdruck	hPa	1951-1980	1001.6	1001.3	1001.9	12.	+0.6		993.9*	06.06.1986
Temperatur (stündl. Ablesung 01-24 MEZ)	°C	1955-1984	16.7	16.6	15.9		-0.7			
Temperatur (Klimamittel)	°C	1909-1969	16.7	16.7	15.9		-0.8			
Höchste Temperatur	°C	1909-1969	30.0	30.4	31.9	18.	+1.5		36.1	20.06.2000
Mittlere Maximum-Temperatur	°C	1909-1969	21.8	21.8	20.7		-1.1			
Tiefste Temperatur	°C	1909-1969	5.3	5.4	3.7	06.	-1.7		0.8	01.06.1977
Mittlere Minimum-Temperatur	°C	1909-1969	11.0	11.4	10.7		-0.7			
Tiefste Temperatur am Erdboden	°C	1951-1980	4.1	3.9	0.4	06.	-3.5		-0.6*	01.06.1977
Mittlere Min.-Temperatur am Erdboden	°C	1951-1980	10.0	*10.1*	7.5		*-2.6*			
Heiße Tage (Max.: ≥ 30,0°C)	Tage	1909-1969	1.4	1.2	2		+1			
Sommertage (Max.: ≥ 25,0°C)	Tage	1909-1969	7.7	7.8	3		-5			
(Max.: ≥ 20,0°C)	Tage			19.4	18		-1			
Frosttage (Min.: < 0,0°C)	Tage	1909-1969	0	0	0		0			
(Min.: ≤ -10,0°C)	Tage			0	0		0			
Eistage (Max.: < 0,0°C)	Tage	1909-1969	0	0	0		0			
Zahl d. Tage Min-Temp. am Erdb. < 0,0°C	Tage	1951-1980	0.1	0.0	0		-0			
Sonnenscheindauer	h	1951-1980	236.2	220.9	172.3		-48.6	78		
in % vom astronomisch möglichen	%		47.0	44.0	34.3		-9.7			
Zahl der sonnenscheinlosen Tage	Tage	1951-1980	1.3	1.3	2		+1			
Bewölkung (in Achtel)	/8	1909-1969	4.9	4.8	5.7		+0.9			
Heitere Tage (< 1,6 Achtel Bewölkung)	Tage	1909-1969	2.8	2.8	0		-3			
Trübe Tage (> 6,4 Achtel Bewölkung)	Tage	1909-1969	8.2	8.0	9		+1			
Dampfdruck	hPa	1909-1969	12.8	13.1	12.5		-0.6			
Relative Luftfeuchtigkeit	%	1909-1969	68	69	70		+1			
Niederschlagshöhe	mm	1909-1969	64.2	70.7	92.3		+21.6	131		
Maximale Tagesmenge	mm	1909-1969	19.6	22.1	21.7	30.	-0.4		57.9	28.06.1964
Zahl der Tage mit Sturzregen	Tage			0.2	-		-			
Zahl der Tage mit ≥ 10,0 mm Niederschlag	Tage	1909-1969	1.7	2.0	2		-0			
Zahl der Tage mit ≥ 5,0 mm Niederschlag	Tage			4.2	7		+3			
Zahl der Tage mit ≥ 2,5 mm Niederschlag	Tage	1909-1969	6.4	7.0	10		+3			
Zahl der Tage mit ≥ 1,0 mm Niederschlag	Tage	1909-1969	9.5	10.2	12		+2			
Zahl der Tage mit ≥ 0,1 mm Niederschlag	Tage	1909-1969	13.4	14.9	18		+3			
Z.d.T.m. gefall. flüss. Niederschl. ≥ 0,1 mm	Tage			14.6	18		+3			
Z.d.T.m. flüss. u. fest. Niederschl. ≥ 0,1 mm	Tage			0.3	0		-0			
Z.d.T.m. gefall. fest. Niederschl. ≥ 0,1 mm	Tage			0	0		0			
Z.d.T.m. abgesetzt. Niederschl. ≥ 0,1 mm	Tage			0.1	-		-			
Z.d.T.m. Hagel ≥ 0,0 mm	Tage			0.2	0		-0			
Z.d.T.m. Graupel, Griesel o. Eisk. ≥ 0,0 mm	Tage			0.0	0		-0			
Zahl der Tage mit Reif oder Rauhreif	Tage	1964-1980	0	0	-		-			
Zahl der Tage mit Tau	Tage	1964-1980	25.4	24.3	-		-			
Zahl der Tage mit Nebel	Tage	1951-1980	0.3	0.3	1		+1			
Zahl der Tage mit Gewitter	Tage	1951-1980	7.4	6.4	4		-2			
Zahl der Tage mit Wetterleuchten	Tage	1951-1980	1.0	0.9	2		+1			
Z.d.T.m. Schneedecke ≥ 0 cm um 7:30 Uhr	Tage	1951-1980	0	0	0		0			
Z.d.T.m. Schneedecke ≥ 1 cm um 7:30 Uhr	Tage	1951-1980	0	0	0		0			
Z.d.T.m. Schneedecke ≥ 5 cm um 7:30 Uhr	Tage			0	0		0			
Z.d.T.m. Neuschnee ≥ 1 cm um 7:30 Uhr	Tage			0	0		0			
Summe der um 7:30 gem. Neuschneemenge	cm									
Max. Höhe der Schneedecke um 7:30 Uhr	cm									
Z.d.T.m. Glatteis durch gefrierenden Regen	Tage	1951-1980	0	0	0		0			
Z.d.T.m. Böen Windst. 6 Bft. (≥ 10,8 m/s)	Tage	1952-1980		12.4	16		+4			
Z.d.T.m. Böen Windst. 8 Bft. (≥ 17,2 m/s)	Tage	1952-1980		0.9	3		+2			
Maximale Windspitze	m/s			18.7	18.9	18.	+0.2		32.2*	23.6.2003
Heizgradsumme					35.6					
Kältesumme		1909-1969	0	0	0		0			

*) 1951-2011 +) 1881-2011

Manfred Wegener

Beiträge zur Berliner Wetterkarte

Klimatologische Mittelwerte von Berlin-Dahlem

44/12 ISSN 0177-3984
KBD VII/11 01.08.2012

Element	Maßeinheit	Beobachtungsperiode	Vieljähr. Durchschnittswert	Durchschnittswert 1961-90	**JULI 2012**	Datum	Abweichung vom Durchschnitt 1961-90	in %	Bisherige Extreme 1909-2011	Datum
Luftdruck auf NN reduziert	hPa	1881-1970	1014.2	1015.5	1014.2		-1.3			
Höchster Luftdruck	hPa	1951-1980	1024.9	1025.4	1030.2	23.	+4.8		1032.4*	16.07.2006
Tiefster Luftdruck	hPa	1951-1980	1001.7	1001.9	999.2	14.	-2.7		989.6*	18.07.1954
Temperatur (stündl. Ablesung 01-24 MEZ)	°C	1955-1984	18.0	17.9	18.4		+0.5			
Temperatur (Klimamittel)	°C	1909-1969	18.3	17.9	18.4		+0.5			
Höchste Temperatur	°C	1909-1969	31.5	30.9	32.0	25.	+1.1		37.8	11.07.1959
Mittlere Maximum-Temperatur	°C	1909-1969	23.5	23.1	23.5		+0.4			
Tiefste Temperatur	°C	1909-1969	8.3	8.0	8.6	23.	+0.6		5.4	09.07.1948
Mittlere Minimum-Temperatur	°C	1909-1969	13.2	12.9	13.4		+0.5			
Tiefste Temperatur am Erdboden	°C	1951-1980	6.6	6.7	6.2	23.	-0.5		4.6*	07.07.1964
Mittlere Min.-Temperatur am Erdboden	°C	1951-1980	11.6	*11.6*	11.2		*-0.4*			
Heiße Tage (Max.: ≥ 30,0°C)	Tage	1909-1969	2.4	2.5	3		+0			
Sommertage (Max.: ≥ 25,0°C)	Tage	1909-1969	10.7	10.3	9		-1			
(Max.: ≥ 20,0°C)	Tage			22.6	26		+3			
Frosttage (Min.: < 0,0°C)	Tage	1909-1969	0	0	0		0			
(Min.: ≤ -10,0°C)	Tage			0	0		0			
Eistage (Max.: < 0,0°C)	Tage	1909-1969	0	0	0		0			
Zahl d. Tage Min-Temp. am Erdb. < 0,0°C	Tage	1951-1980	0	0	0		0			
Sonnenscheindauer	h	1951-1980	220.6	218.0	220.3		+2.3	101		
in % vom astronomisch möglichen	%		43.7	43.2	43.7		+0.5			
Zahl der sonnenscheinlosen Tage	Tage	1951-1980	1.1	0.8	0		-1			
Bewölkung (in Achtel)	/8	1909-1969	5.0	4.8	4.9		+0.1			
Heitere Tage (< 1,6 Achtel Bewölkung)	Tage	1909-1969	2.1	3.1	1		-2			
Trübe Tage (> 6,4 Achtel Bewölkung)	Tage	1909-1969	9.1	8.4	4		-4			
Dampfdruck	hPa	1909-1969	15.0	14.4	15.1		+0.7			
Relative Luftfeuchtigkeit	%	1909-1969	72	70	73		+3			
Niederschlagshöhe	mm	1909-1969	72.1	53.1	121.8		+68.7	229		
Maximale Tagesmenge	mm	1909-1969	21.3	15.6	29.7	05.	+14.1		65.3	26.07.1930
Zahl der Tage mit Sturzregen	Tage			0.2	-		-			
Zahl der Tage mit ≥ 10,0 mm Niederschlag	Tage	1909-1969	1.9	1.2	5		+4			
Zahl der Tage mit ≥ 5,0 mm Niederschlag	Tage			3.5	8		+4			
Zahl der Tage mit ≥ 2,5 mm Niederschlag	Tage	1909-1969	7.3	5.9	12		+6			
Zahl der Tage mit ≥ 1,0 mm Niederschlag	Tage	1909-1969	10.4	9.0	15		+6			
Zahl der Tage mit ≥ 0,1 mm Niederschlag	Tage	1909-1969	14.6	13.9	20		+6			
Z.d.T.m. gefall. flüss. Niederschl. ≥ 0,1 mm	Tage			13.6	18		+4			
Z.d.T.m. flüss. u. fest. Niederschl. ≥ 0,1 mm	Tage			0.2	2		+2			
Z.d.T.m. gefall. fest. Niederschl. ≥ 0,1 mm	Tage			0	0		0			
Z.d.T.m. abgesetzt. Niederschl. ≥ 0,1 mm	Tage			0.1	-		-			
Z.d.T.m. Hagel ≥ 0,0 mm	Tage			0.2	2		+2			
Z.d.T.m. Graupel, Griesel o. Eisk. ≥ 0,0 mm	Tage			0.0	0		-0			
Zahl der Tage mit Reif oder Rauhreif	Tage	1964-1980	0	0	-		-			
Zahl der Tage mit Tau	Tage	1964-1980	27.7	26.1	-		-			
Zahl der Tage mit Nebel	Tage	1951-1980	0.6	0.3	0		-0			
Zahl der Tage mit Gewitter	Tage	1951-1980	5.9	5.3	7		+2			
Zahl der Tage mit Wetterleuchten	Tage	1951-1980	0.8	0.6	5		+4			
Z.d.T.m. Schneedecke ≥ 0 cm um 7:30 Uhr	Tage	1951-1980	0	0	0		0			
Z.d.T.m. Schneedecke ≥ 1 cm um 7:30 Uhr	Tage	1951-1980	0	0	0		0			
Z.d.T.m. Schneedecke ≥ 5 cm um 7:30 Uhr	Tage			0	0		0			
Z.d.T.m. Neuschnee ≥ 1 cm um 7:30 Uhr	Tage		0	0	0		0			
Summe der um 7:30 gem. Neuschneemenge	cm			.						
Max. Höhe der Schneedecke um 7:30 Uhr	cm									
Z.d.T.m. Glatteis durch gefrierenden Regen	Tage	1951-1980	0	0	0		0			
Z.d.T.m. Böen Windst. 6 Bft. (≥ 10,8 m/s)	Tage	1952-1980		12.0	19		+7			
Z.d.T.m. Böen Windst. 8 Bft. (≥ 17,2 m/s)	Tage	1952-1980		0.7	3		+2			
Maximale Windspitze	m/s			18.1	18.6	19.	+0.5		33.2*	10.7.2002
Heizgradsumme										
Kältesumme		1909-1969		0	0		0			

*) 1951-2011 +) 1881-2011

Manfred Wegener

Beiträge zur Berliner Wetterkarte

Klimatologische Mittelwerte von Berlin-Dahlem

53/12 ISSN 0177-3984
KBD VIII/12 03.09.2012

Element	Maß-einheit	Beob-achtungs-periode	Vieljähr. Durch-schnitts-wert	Durch-schnitts-wert 1961-90	**August 2012**	Datum	Abweichung vom Durchschnitt 1961-90	in %	Bisherige Extreme 1909-2011	Datum
Luftdruck auf NN reduziert	hPa	1881-1970	1014.4	1015.5	1016.5		+1.0			
Höchster Luftdruck	hPa	1951-1980	1025.8	1026.4	1027.6	21.	+1.2		1031.5+	04.08.1981
Tiefster Luftdruck	hPa	1951-1980	1000.9	1001.6	987.2	15.	-14.4		984.2+	25.08.1956
Temperatur (stündl. Ablesung 01-24 MEZ)	°C	1955-1984	17.3	17.3	18.5		+1.2			
Temperatur (Klimamittel)	°C	1909-1969	17.3	17.2	18.5		+1.3			
Höchste Temperatur	°C	1909-1969	30.4	30.9	35.1	19.	+4.2		37.7	01.08.1994
Mittlere Maximum-Temperatur	°C	1909-1969	22.7	22.8	24.0		+1.2			
Tiefste Temperatur	°C	1909-1969	7.6	7.0	8.6	28.	+1.6		4.7	21.08.1964
Mittlere Minimum-Temperatur	°C	1909-1969	12.5	12.4	13.3		+0.9			
Tiefste Temperatur am Erdboden	°C	1951-1980	5.8	*5.8*	5.2	28.	-0.6		3.2*	21.08.1964
Mittlere Min.-Temperatur am Erdboden	°C	1951-1980	11.1	*11.2*	9.8		*-1.4*			
Heiße Tage (Max.: ≥ 30,0°C)	Tage	1909-1969	1.6	1.6	3		+1			
Sommertage (Max.: ≥ 25,0°C)	Tage	1909-1969	8.6	9.1	8		-1			
(Max.: ≥ 20,0°C)	Tage			23.0	28		+5			
Frosttage (Min.: < 0,0°C)	Tage	1909-1969	0	0	0		0			
(Min.: ≤ -10,0°C)	Tage			0	0		0			
Eistage (Max.: < 0,0°C)	Tage	1909-1969	0	0	0		0			
Zahl d. Tage Min-Temp. am Erdb. < 0,0°C	Tage	1951-1980	0	0	0		0			
Sonnenscheindauer	h	1951-1980	209.1	210.2	251.6		+41.4	120		
in % vom astronomisch möglichen	%		45.9	46.2	55.3		+9.1			
Zahl der sonnenscheinlosen Tage	Tage	1951-1980	1.1	1.2	1		-0			
Bewölkung (in Achtel)	/8	1909-1969	4.8	4.5	4.3		-0.2			
Heitere Tage (< 1,6 Achtel Bewölkung)	Tage	1909-1969	2.9	4.3	2		-2			
Trübe Tage (> 6,4 Achtel Bewölkung)	Tage	1909-1969	8.2	7.0	3		-4			
Dampfdruck	hPa	1909-1969	14.7	14.4	14.9		+0.5			
Relative Luftfeuchtigkeit	%	1909-1969	75	73	72		-1			
Niederschlagshöhe	mm	1909-1969	66.0	65.3	39.5		-25.8	61		
Maximale Tagesmenge	mm	1909-1969	21.0	24.6	18.3	21.	-6.3		124.7	14.08.1948
Zahl der Tage mit Sturzregen	Tage			0.4	-		-			
Zahl der Tage mit ≥ 10,0 mm Niederschlag	Tage	1909-1969	1.9	1.9	1		-1			
Zahl der Tage mit ≥ 5,0 mm Niederschlag	Tage			3.6	2		-2			
Zahl der Tage mit ≥ 2,5 mm Niederschlag	Tage	1909-1969	6.4	6.2	3		-3			
Zahl der Tage mit ≥ 1,0 mm Niederschlag	Tage	1909-1969	9.8	9.0	7		-2			
Zahl der Tage mit ≥ 0,1 mm Niederschlag	Tage	1909-1969	14.3	13.4	14		+1			
Z.d.T.m. gefall. flüss. Niederschl. ≥ 0,1 mm	Tage			12.6	14		+1			
Z.d.T.m. flüss. u. fest. Niederschl. ≥ 0,1 mm	Tage			0.4	0		-0			
Z.d.T.m. gefall. fest. Niederschl. ≥ 0,1 mm	Tage			0	0		0			
Z.d.T.m. abgesetzt. Niederschl. ≥ 0,1 mm	Tage			0.4	-		-			
Z.d.T.m. Hagel ≥ 0,0 mm	Tage			0.4	0		-0			
Z.d.T.m. Graupel, Griesel o. Eisk. ≥ 0,0 mm	Tage			0.0	0		-0			
Zahl der Tage mit Reif oder Rauhreif	Tage	1964-1980	0	0	-		-			
Zahl der Tage mit Tau	Tage	1964-1980	27.2	27.0	-		-			
Zahl der Tage mit Nebel	Tage	1951-1980	1.3	1.0	1		-0			
Zahl der Tage mit Gewitter	Tage	1951-1980	5.3	4.8	3		-2			
Zahl der Tage mit Wetterleuchten	Tage	1951-1980	1.6	1.3	2		+1			
Z.d.T.m. Schneedecke ≥ 0 cm um 7:30 Uhr	Tage	1951-1980	0	0	0		0			
Z.d.T.m. Schneedecke ≥ 1 cm um 7:30 Uhr	Tage	1951-1980	0	0	0		0			
Z.d.T.m. Schneedecke ≥ 5 cm um 7:30 Uhr	Tage			0	0		0			
Z.d.T.m. Neuschnee ≥ 1 cm um 7:30 Uhr	Tage			0	0		0			
Summe der um 7:30 gem. Neuschneemenge	cm				.					
Max. Höhe der Schneedecke um 7:30 Uhr	cm				.					
Z.d.T.m. Glatteis durch gefrierenden Regen	Tage	1951-1980	0	0	0		0			
Z.d.T.m. Böen Windst. 6 Bft. (≥ 10,8 m/s)	Tage	1952-1980		9.5	10		+1			
Z.d.T.m. Böen Windst. 8 Bft. (≥ 17,2 m/s)	Tage	1952-1980		0.7	0		-1			
Maximale Windspitze	m/s			17.7	16.5	26.	-1.2		26.3*	04.08.1974
Heizgradsumme										
Kältesumme		1909-1969	0	0	0		0			

*) 1951-2011 +) 1881-2011

Manfred Wegener

Beiträge zur Berliner Wetterkarte

Klimatologische Mittelwerte von Berlin-Dahlem

58/12
KBD IX/12

ISSN 0177-3984
02.10.2012

Element	Maßeinheit	Beobachtungsperiode	Vieljähr. Durchschnittswert	Durchschnittswert 1961-90	**September 2012**	Datum	Abweichung vom Durchschnitt 1961-90	in %	Bisherige Extreme 1909-2011	Datum
Luftdruck auf NN reduziert	hPa	1881-1970	1016.5	1016.7	1015.4		-1.3			
Höchster Luftdruck	hPa	1951-1980	1030.1	1030.0	1026.9	02.	-3.1		1038.5+	07.09.1953
Tiefster Luftdruck	hPa	1951-1980	1000.8	1000.4	998.8	24.	-1.6		987.2+	21.09.1990
Temperatur (stündl. Ablesung 01-24 MEZ)	°C	1955-1984	13.7	13.6	14.4		+0.8			
Temperatur (Klimamittel)	°C	1909-1969	13.9	13.5	14.4		+0.9			
Höchste Temperatur	°C	1909-1969	27.0	26.1	28.8	11.	+2.7		34.2	03.09.1911<br>12.9.1919
Mittlere Maximum-Temperatur	°C	1909-1969	19.3	18.7	20.0		+1.3			
Tiefste Temperatur	°C	1909-1969	3.4	4.0	4.2	21.	+0.2		-0.5	21.09.1915
Mittlere Minimum-Temperatur	°C	1909-1969	9.4	9.4	9.2		-0.2			
Tiefste Temperatur am Erdboden	°C	1951-1980	1.8	2.6	-0.1	23.	-2.7		-1.9*	21.09.1997
Mittlere Min.-Temperatur am Erdboden	°C	1951-1980	7.9	*8.3*	5.6		*-2.7*			
Heiße Tage (Max.: ≥ 30,0°C)	Tage	1909-1969	0.4	0.1	0		-0			
Sommertage (Max.: ≥ 25,0°C)	Tage	1909-1969	2.9	2.1	2		-0			
(Max.: ≥ 20,0°C)	Tage			10.8	13		+2			
Frosttage (Min.: < 0,0°C)	Tage	1909-1969	0	0	0		0			
(Min.: ≤ -10,0°C)	Tage			0	0		0			
Eistage (Max.: < 0,0°C)	Tage	1909-1969	0	0	0		0			
Zahl d. Tage Min-Temp. am Erdb. < 0,0°C	Tage	1951-1980	0.3	0.1	1		+1			
Sonnenscheindauer	h	1951-1980	171.5	156.3	206.0		+49.7	132		
in % vom astronomisch möglichen	%		45.0	41.0	54.0		+13.0			
Zahl der sonnenscheinlosen Tage	Tage	1951-1980	2.2	2.4	1		-1			
Bewölkung (in Achtel)	/8	1909-1969	4.6	4.7	4.5		-0.2			
Heitere Tage (< 1,6 Achtel Bewölkung)	Tage	1909-1969	4.3	3.7	2		-2			
Trübe Tage (> 6,4 Achtel Bewölkung)	Tage	1909-1969	7.4	8.4	6		-2			
Dampfdruck	hPa	1909-1969	12.5	12.6	11.7		-0.9			
Relative Luftfeuchtigkeit	%	1909-1969	79	80	73		-7			
Niederschlagshöhe	mm	1909-1969	45.7	45.5	26.9		-18.6	59		
Maximale Tagesmenge	mm	1909-1969	14.2	15.4	5.2	27.	-10.2		40.0	05.09.1931
Zahl der Tage mit Sturzregen	Tage			0.1	-		-			
Zahl der Tage mit ≥ 10,0 mm Niederschlag	Tage	1909-1969	1.0	1.0	0		-1			
Zahl der Tage mit ≥ 5,0 mm Niederschlag	Tage			2.7	1		-2			
Zahl der Tage mit ≥ 2,5 mm Niederschlag	Tage	1909-1969	5.5	5.2	6		+1			
Zahl der Tage mit ≥ 1,0 mm Niederschlag	Tage	1909-1969	8.4	8.6	7		-2			
Zahl der Tage mit ≥ 0,1 mm Niederschlag	Tage	1909-1969	12.6	14.4	10		-4			
Z.d.T.m. gefall. flüss. Niederschl. ≥ 0,1 mm	Tage			14.0	10		-4			
Z.d.T.m. flüss. u. fest. Niederschl. ≥ 0,1 mm	Tage			0.2	0		-0			
Z.d.T.m. gefall. fest. Niederschl. ≥ 0,1 mm	Tage			0.0	0		-0			
Z.d.T.m. abgesetzt. Niederschl. ≥ 0,1 mm	Tage			0.2	-		-			
Z.d.T.m. Hagel ≥ 0,0 mm	Tage			0.1	0		-0			
Z.d.T.m. Graupel, Griesel o. Eisk. ≥ 0,0 mm	Tage			0.1	0		-0			
Zahl der Tage mit Reif oder Rauhreif	Tage	1964-1980	0.2	0.1	-		-			
Zahl der Tage mit Tau	Tage	1964-1980	27.4	27.2	-		-			
Zahl der Tage mit Nebel	Tage	1951-1980	2.4	1.8	0		-2			
Zahl der Tage mit Gewitter	Tage	1951-1980	2.4	2.4	1		-1			
Zahl der Tage mit Wetterleuchten	Tage	1951-1980	0.6	0.5	1		+0			
Z.d.T.m. Schneedecke ≥ 0 cm um 7:30 Uhr	Tage	1951-1980	0	0	0		0			
Z.d.T.m. Schneedecke ≥ 1 cm um 7:30 Uhr	Tage	1951-1980	0	0	0		0			
Z.d.T.m. Schneedecke ≥ 5 cm um 7:30 Uhr	Tage			0	0		0			
Z.d.T.m. Neuschnee ≥ 1 cm um 7:30 Uhr	Tage			0	0		0			
Summe der um 7:30 gem. Neuschneemenge	cm			.						
Max. Höhe der Schneedecke um 7:30 Uhr	cm			.						
Z.d.T.m. Glatteis durch gefrierenden Regen	Tage	1951-1980	0	0	0		0			
Z.d.T.m. Böen Windst. 6 Bft. (≥ 10,8 m/s)	Tage	1952-1980		9.8	16		+6			
Z.d.T.m. Böen Windst. 8 Bft. (≥ 17,2 m/s)	Tage	1952-1980		1.2	1		-0			
Maximale Windspitze	m/s			18.5	19.4	22.	+0.9		24.7*	9.9.1997
Heizgradsumme										
Kältesumme		1909-1969	0	0	0		0			

*) 1951-2011 +) 1881-2011

Diana Schmiedel

Beiträge zur Berliner Wetterkarte

Klimatologische Mittelwerte von Berlin-Dahlem

64/12
KBD 10/12

ISSN 0177-3984
01.11.2012

Element	Maß-einheit	Beob-achtungs-periode	Vieljähr. Durch-schnitts-wert	Durch-schnitts-wert 1961-90	**Oktober 2012**	Datum	Abweichung vom Durchschnitt 1961-90	in %	Bisherige Extreme 1909-2011	Datum
Luftdruck auf NN reduziert	hPa	1881-1970	1015.9	1017.3	1013.0		-4.3			
Höchster Luftdruck	hPa	1951-1980	1032.5	1033.6	1028.0	23.	-5.6		1042.1+	18.10.1993
Tiefster Luftdruck	hPa	1951-1980	998.1	996.0	998.5	15.	+2.5		979.3+	22.10.1974
Temperatur (stündl. Ablesung 01-24 MEZ)	°C	1955-1984	9.1	9.3	9.0		-0.3			
Temperatur (Klimamittel)	°C	1909-1969	9.0	9.3	9.0		-0.3			
Höchste Temperatur	°C	1909-1969	20.5	21.3	22.5	19.	+1.2		27.5	04.10.1985
Mittlere Maximum-Temperatur	°C	1909-1969	13.1	13.3	13.9		+0.6			
Tiefste Temperatur	°C	1909-1969	-1.1	-0.4	-5.0	29.	-4.6		-9.6	29.10.1915
Mittlere Minimum-Temperatur	°C	1909-1969	5.4	5.9	4.6		-1.3			
Tiefste Temperatur am Erdboden	°C	1951-1980	-2.0	*-2.1*	-7.2	29.	*-5.1*		-8.8*	28.10.1997
Mittlere Min.-Temperatur am Erdboden	°C	1951-1980	4.5	*4.6*	1.7		*-2.9*			
Heiße Tage (Max.: ≥ 30,0°C)	Tage	1909-1969	0	0	0		0			
Sommertage (Max.: ≥ 25,0°C)	Tage	1909-1969	0.1	0.1	0		-0			
(Max.: ≥ 20,0°C)	Tage			2.0	4		+2			
Frosttage (Min.: < 0,0°C)	Tage	1909-1969	2.3	1.3	2		+1			
(Min.: ≤ -10,0°C)	Tage			0	0		0			
Eistage (Max.: < 0,0°C)	Tage	1909-1969	0.0	0	0		0			
Zahl d. Tage Min-Temp. am Erdb. < 0,0°C	Tage	1951-1980	3.7	3.6	12		+8			
Sonnenscheindauer	h	1951-1980	112.2	110.8	147.9		+37.1	133		
in % vom astronomisch möglichen	%		33.9	33.5	44.7		+11.2			
Zahl der sonnenscheinlosen Tage	Tage	1951-1980	6.6	6.4	3		-3			
Bewölkung (in Achtel)	/8	1909-1969	5.2	5.2	4.8		-0.4			
Heitere Tage (< 1,6 Achtel Bewölkung)	Tage	1909-1969	3.3	3.0	1		-2			
Trübe Tage (> 6,4 Achtel Bewölkung)	Tage	1909-1969	11.6	11.3	7		-4			
Dampfdruck	hPa	1909-1969	9.7	10.0	9.3		-0.7			
Relative Luftfeuchtigkeit	%	1909-1969	83	83	81		-2			
Niederschlagshöhe	mm	1909-1969	44.2	35.8	35.3		-0.5	99		
Maximale Tagesmenge	mm	1909-1969	11.8	9.4	9.7	06.	+0.3		36.3	19.10.1941
Zahl der Tage mit Sturzregen	Tage			0	-		-			
Zahl der Tage mit ≥ 10,0 mm Niederschlag	Tage	1909-1969	0.8	0.5	0		-1			
Zahl der Tage mit ≥ 5,0 mm Niederschlag	Tage			2.4	3		+1			
Zahl der Tage mit ≥ 2,5 mm Niederschlag	Tage	1909-1969	5.5	4.8	5		+0			
Zahl der Tage mit ≥ 1,0 mm Niederschlag	Tage	1909-1969	8.9	7.9	8		+0			
Zahl der Tage mit ≥ 0,1 mm Niederschlag	Tage	1909-1969	13.6	14.3	14		-0			
Z.d.T.m. gefall. flüss. Niederschl. ≥ 0,1 mm	Tage			13.1	12		-1			
Z.d.T.m. flüss. u. fest. Niederschl. ≥ 0,1 mm	Tage			0.6	2		+1			
Z.d.T.m. gefall. fest. Niederschl. ≥ 0,1 mm	Tage			0	0		0			
Z.d.T.m. abgesetzt. Niederschl. ≥ 0,1 mm	Tage			0.6	-		-			
Z.d.T.m. Hagel ≥ 0,0 mm	Tage			0.2	0		-0			
Z.d.T.m. Graupel, Griesel o. Eisk. ≥ 0,0 mm	Tage			0.4	1		+1			
Zahl der Tage mit Reif oder Rauhreif	Tage	1964-1980	3.8	3.3	-		-			
Zahl der Tage mit Tau	Tage	1964-1980	23.8	24.5	-		-			
Zahl der Tage mit Nebel	Tage	1951-1980	6.6	5.9	4		-2			
Zahl der Tage mit Gewitter	Tage	1951-1980	0.3	0.4	1		+1			
Zahl der Tage mit Wetterleuchten	Tage	1951-1980	0.0	0.0	0		-0			
Z.d.T.m. Schneedecke ≥ 0 cm um 7:30 Uhr	Tage	1951-1980	0	0	0		0			
Z.d.T.m. Schneedecke ≥ 1 cm um 7:30 Uhr	Tage	1951-1980	0	0	0		0			
Z.d.T.m. Schneedecke ≥ 5 cm um 7:30 Uhr	Tage			0	0		0			
Z.d.T.m. Neuschnee ≥ 1 cm um 7:30 Uhr	Tage			0	0		0			
Summe der um 7:30 gem. Neuschneemenge	cm									
Max. Höhe der Schneedecke um 7:30 Uhr	cm								0 Fl	14.10.2002
Z.d.T.m. Glatteis durch gefrierenden Regen	Tage	1951-1980	0	0.0	0		-0			
Z.d.T.m. Böen Windst. 6 Bft. (≥ 10,8 m/s)	Tage	1952-1980		10.5	15		+4			
Z.d.T.m. Böen Windst. 8 Bft. (≥ 17,2 m/s)	Tage	1952-1980		1.3	4		+3			
Maximale Windspitze	m/s			19.8	28.0	04.	+8.2		29.7*	27.10.2002
Heizgradsumme					347.6					
Kältesumme		1909-1969	0.4	0	0		0			

*) 1951-2011 +) 1881-2011

Diana Schmiedel

Beiträge zur Berliner Wetterkarte

Klimatologische Mittelwerte von Berlin-Dahlem

69/12 ISSN 0177-3984
KBD11/12 04.12.2012

Element	Maßeinheit	Beobachtungsperiode	Vieljähr. Durchschnittswert	Durchschnittswert 1961-90	**November 2012**	Datum	Abweichung vom Durchschnitt 1961-90	in %	Bisherige Extreme 1909-2011	Datum
Luftdruck auf NN reduziert	hPa	1881-1970	1015.4	1014.8	1013.8		-1.0			
Höchster Luftdruck	hPa	1951-1980	1032.9	1034.0	1034.6	13.	+0.6		1043.3+	21.11.1998
Tiefster Luftdruck	hPa	1951-1980	991.0	988.2	989.6	01.	+1.4		966.8+	27.11.1983
Temperatur (stündl. Ablesung 01-24 MEZ)	°C	1955-1984	4.5	4.5	5.3		+0.8			
Temperatur (Klimamittel)	°C	1909-1969	4.2	4.6	5.3		+0.7			
Höchste Temperatur	°C	1909-1969	13.5	14.1	11.6	11.	-2.5		19.5	01.11.1968
Mittlere Maximum-Temperatur	°C	1909-1969	6.7	7.0	7.7		+0.7			
Tiefste Temperatur	°C	1909-1969	-4.7	-4.8	-3.2	15.	+1.6		-16.1	23.11.1965
Mittlere Minimum-Temperatur	°C	1909-1969	1.7	2.1	2.7		+0.6			
Tiefste Temperatur am Erdboden	°C	1951-1980	-6.4	*-6.7*	-6.2	15.	*+0.5*		-24.2*	23.11.1965
Mittlere Min.-Temperatur am Erdboden	°C	1951-1980	1.0	*1.1*	0.6		*-0.5*			
Heiße Tage (Max.: ≥ 30,0°C)	Tage	1909-1969	0	0	0		0			
Sommertage (Max.: ≥ 25,0°C)	Tage	1909-1969	0	0	0		0			
(Max.: ≥ 20,0°C)	Tage			0	0		0			
Frosttage (Min.: < 0,0°C)	Tage	1909-1969	9.5	8.4	8		-0			
(Min.: ≤ -10,0°C)	Tage			0.1	0		-0			
Eistage (Max.: < 0,0°C)	Tage	1909-1969	1.3	1.3	1		-0			
Zahl d. Tage Min-Temp. am Erdb. < 0,0°C	Tage	1951-1980	11.2	11.5	13		+1			
Sonnenscheindauer	h	1951-1980	47.7	52.4	45.5		-6.9	87		
in % vom astronomisch möglichen	%		18.0	19.8	17.2		-2.6			
Zahl der sonnenscheinlosen Tage	Tage	1951-1980	13.2	12.0	13		+1			
Bewölkung (in Achtel)	/8	1909-1969	6.2	6.0	6.2		+0.2			
Heitere Tage (< 1,6 Achtel Bewölkung)	Tage	1909-1969	1.3	1.3	2		+1			
Trübe Tage (> 6,4 Achtel Bewölkung)	Tage	1909-1969	16.3	16.1	17		+1			
Dampfdruck	hPa	1909-1969	7.3	7.4	7.8		+0.4			
Relative Luftfeuchtigkeit	%	1909-1969	87	85	87		+2			
Niederschlagshöhe	mm	1909-1969	47.7	49.5	45.4		-4.1	92		
Maximale Tagesmenge	mm	1909-1969	12.7	14.0	14.7	29.	+0.7		47.5	27.11.1926
Zahl der Tage mit Sturzregen	Tage			0	-		-			
Zahl der Tage mit ≥ 10,0 mm Niederschlag	Tage	1909-1969	1.0	0.9	1		+0			
Zahl der Tage mit ≥ 5,0 mm Niederschlag	Tage			3.1	1		-2			
Zahl der Tage mit ≥ 2,5 mm Niederschlag	Tage	1909-1969	5.7	6.3	7		+1			
Zahl der Tage mit ≥ 1,0 mm Niederschlag	Tage	1909-1969	9.8	10.1	10		-0			
Zahl der Tage mit ≥ 0,1 mm Niederschlag	Tage	1909-1969	16.1	17.0	17		-0			
Z.d.T.m. gefall. flüss. Niederschl. ≥ 0,1 mm	Tage			12.2	16		+4			
Z.d.T.m. flüss. u. fest. Niederschl. ≥ 0,1 mm	Tage			3.7	1		-3			
Z.d.T.m. gefall. fest. Niederschl. ≥ 0,1 mm	Tage			1.0	0		-1			
Z.d.T.m. abgesetzt. Niederschl. ≥ 0,1 mm	Tage			0.1	-		-			
Z.d.T.m. Hagel ≥ 0,0 mm	Tage			0.2	0		-0			
Z.d.T.m. Graupel, Griesel o. Eisk. ≥ 0,0 mm	Tage			2.5	2		-1			
Zahl der Tage mit Reif oder Rauhreif	Tage	1964-1980	9.5	9.7	-		-			
Zahl der Tage mit Tau	Tage	1964-1980	12.1	13.6	-		-			
Zahl der Tage mit Nebel	Tage	1951-1980	6.1	5.4	8		+3			
Zahl der Tage mit Gewitter	Tage	1951-1980	0.4	0.5	0		-1			
Zahl der Tage mit Wetterleuchten	Tage	1951-1980	0.0	0.1	0		-0			
Z.d.T.m. Schneedecke ≥ 0 cm um 7:30 Uhr	Tage	1951-1980	1.8	2.6	0		-3			
Z.d.T.m. Schneedecke ≥ 1 cm um 7:30 Uhr	Tage	1951-1980	1.6	2.2	0		-2			
Z.d.T.m. Schneedecke ≥ 5 cm um 7:30 Uhr	Tage			1.0	0		-1			
Z.d.T.m. Neuschnee ≥ 1 cm um 7:30 Uhr	Tage			1.2	0		-1			
Summe der um 7:30 gem. Neuschneemenge	cm									
Max. Höhe der Schneedecke um 7:30 Uhr	cm			3.4			-3		27	17./18.11.1919
Z.d.T.m. Glatteis durch gefrierenden Regen	Tage	1951-1980	0.5	0.5	0		-1			
Z.d.T.m. Böen Windst. 6 Bft. (≥ 10,8 m/s)	Tage	1952-1980		13.3	9		-4			
Z.d.T.m. Böen Windst. 8 Bft. (≥ 17,2 m/s)	Tage	1952-1980		2.7	0		-3			
Maximale Windspitze	m/s			22.1	16.2	07.	-5.9		36.5*	13.11.1972
Heizgradsumme					446.1					
Kältesumme		1909-1969	7.3	6.5	2.0		-4.5			

*) 1951-2011 +) 1881-2011

Diana Schmiedel

Beiträge zur Berliner Wetterkarte

Klimatologische Mittelwerte von Berlin-Dahlem

01/13 ISSN 0177-3984
KBD 12/12 03.01.2013

Element	Maß-einheit	Beob-achtungs-periode	Vieljähr. Durch-schnitts-wert	Durch-schnitts-wert 1961-90	**Dezember 2012**	Datum	Abweichung vom Durchschnitt 1961-90	in %	Bisherige Extreme 1909-2011	Datum
Luftdruck auf NN reduziert	hPa	1881-1970	1015.2	1015.1	1011.3		-3.8			
Höchster Luftdruck	hPa	1951-1980	1034.8	1036.6	1029.2	28.	-7.4		1049.4+	23.12.1963
Tiefster Luftdruck	hPa	1951-1980	987.7	988.3	991.6	04.	+3.3		970.2+	16.12.1962
Temperatur (stündl. Ablesung 01-24 MEZ)	°C	1955-1984	0.9	1.1	0.6		-0.5			
Temperatur (Klimamittel)	°C	1909-1969	0.9	1.2	0.6		-0.6			
Höchste Temperatur	°C	1909-1969	9.9	10.5	12.2	25.	+1.7		15.7	24.12.1977
Mittlere Maximum-Temperatur	°C	1909-1969	3.0	3.2	2.8		-0.4			
Tiefste Temperatur	°C	1909-1969	-9.6	-9.2	-11.0	08.	-1.8		-20.2	21.12.1969
Mittlere Minimum-Temperatur	°C	1909-1969	-1.5	-1.1	-1.9		-0.8			
Tiefste Temperatur am Erdboden	°C	1951-1980	-11.3	*-11.9*	-15.4	08.	*-3.5*		-24.0*	21.12.1969
Mittlere Min.-Temperatur am Erdboden	°C	1951-1980	-1.9	*-2.1*	-3.7		*-1.6*			
Heiße Tage (Max.: ≥ 30,0°C)	Tage	1909-1969	0	0	0		0			
Sommertage (Max.: ≥ 25,0°C)	Tage	1909-1969	0	0	0		0			
(Max.: ≥ 20,0°C)	Tage			0	0		0			
Frosttage (Min.: < 0,0°C)	Tage	1909-1969	17.4	17.0	20		+3			
(Min.: ≤ -10,0°C)	Tage			1.6	1		-1			
Eistage (Max.: < 0,0°C)	Tage	1909-1969	6.3	7.2	9		+2			
Zahl d. Tage Min-Temp. am Erdb. < 0,0°C	Tage	1951-1980	19.4	19.9	25		+5			
Sonnenscheindauer	h	1951-1980	35.7	37.4	36.1		-1.3	97		
in % vom astronomisch möglichen	%		14.9	15.6	15.1		-0.5			
Zahl der sonnenscheinlosen Tage	Tage	1951-1980	16.5	16.3	15		-1			
Bewölkung (in Achtel)	/8	1909-1969	6.2	6.2	6.5		+0.3			
Heitere Tage (< 1,6 Achtel Bewölkung)	Tage	1909-1969	1.5	2.0	1		-1			
Trübe Tage (> 6,4 Achtel Bewölkung)	Tage	1909-1969	17.7	18.4	19		+1			
Dampfdruck	hPa	1909-1969	6.0	6.1	5.6		-0.5			
Relative Luftfeuchtigkeit	%	1909-1969	88	86	86		0			
Niederschlagshöhe	mm	1909-1969	47.4	54.5	41.0		-13.5	75		
Maximale Tagesmenge	mm	1909-1969	10.3	10.8	8.4	23.	-2.4		23.3	10.12.1990
Zahl der Tage mit Sturzregen	Tage			0	-		-			
Zahl der Tage mit ≥ 10,0 mm Niederschlag	Tage	1909-1969	0.9	0.9	0		-1			
Zahl der Tage mit ≥ 5,0 mm Niederschlag	Tage			4.1	3		-1			
Zahl der Tage mit ≥ 2,5 mm Niederschlag	Tage	1909-1969	6.5	7.2	4		-3			
Zahl der Tage mit ≥ 1,0 mm Niederschlag	Tage	1909-1969	10.1	11.2	13		+2			
Zahl der Tage mit ≥ 0,1 mm Niederschlag	Tage	1909-1969	16.0	18.2	21		+3			
Z.d.T.m. gefall. flüss. Niederschl. ≥ 0,1 mm	Tage			9.0	8		-1			
Z.d.T.m. flüss. u. fest. Niederschl. ≥ 0,1 mm	Tage			5.3	5		-0			
Z.d.T.m. gefall. fest. Niederschl. ≥ 0,1 mm	Tage			3.7	8		+4			
Z.d.T.m. abgesetzt. Niederschl. ≥ 0,1 mm	Tage			0.2	-		-			
Z.d.T.m. Hagel ≥ 0,0 mm	Tage			0.1	0		-0			
Z.d.T.m. Graupel, Griesel o. Eisk. ≥ 0,0 mm	Tage			5.9	8		+2			
Zahl der Tage mit Reif oder Rauhreif	Tage	1964-1980	13.1	14.0	-		-			
Zahl der Tage mit Tau	Tage	1964-1980	6.3	6.8	-		-			
Zahl der Tage mit Nebel	Tage	1951-1980	6.3	4.9	5		+0			
Zahl der Tage mit Gewitter	Tage	1951-1980	0.3	0.3	0		-0			
Zahl der Tage mit Wetterleuchten	Tage	1951-1980	0.0	0.0	0		-0			
Z.d.T.m. Schneedecke ≥ 0 cm um 7:30 Uhr	Tage	1951-1980	8.8	9.4	18		+9			
Z.d.T.m. Schneedecke ≥ 1 cm um 7:30 Uhr	Tage	1951-1980	7.5	8.2	16		+8			
Z.d.T.m. Schneedecke ≥ 5 cm um 7:30 Uhr	Tage			4.3	8		+4			
Z.d.T.m. Neuschnee ≥ 1 cm um 7:30 Uhr	Tage			3.7	8		+4			
Summe der um 7:30 gem. Neuschneemenge	cm				23					
Max. Höhe der Schneedecke um 7:30 Uhr	cm			6.7	14	12.	+7		43	28.12.2010
Z.d.T.m. Glatteis durch gefrierenden Regen	Tage	1951-1980	1.5	1.7	3		+1			
Z.d.T.m. Böen Windst. 6 Bft. (≥ 10,8 m/s)	Tage	1952-1980		13.8	14		+0			
Z.d.T.m. Böen Windst. 8 Bft. (≥ 17,2 m/s)	Tage	1952-1980		2.3	0		-2			
Maximale Windspitze	m/s			21.2	15.9	27.	-5.3		33.0*	9.12.1993
Heizgradsumme					615.1					
Kältesumme		1909-1969	39.9	40.4	44.0		+3.6			

*) 1951-2011 +) 1881-2011

Diana Schmiedel
